# Stereoselective Generalizations over Diverse Sets of Chiral Acids Enabled by Buried Volume

**DOI:** 10.1021/jacs.5c20342

**Published:** 2026-01-06

**Authors:** Andrew L. Smith, F. Dean Toste

**Affiliations:** † Department of Chemistry, 1438 University of California, Berkeley, California 94720, United States; ‡ Chemical Sciences Division, Lawrence Berkeley National Laboratory, Berkeley, California 94720, United States

## Abstract

Stereoselective catalysis is a paradigm of complexity, navigating arrays of specific, yet weak, noncovalent interactions to achieve asymmetric induction. Accordingly, structure–activity relationships modeling stereoselectivity have required bespoke representations, high dimensionality, and/or large data sets to furnish productive trends, hampering their subsequent interpretability and utility. Here, we report that active site-based buried volume is a uniquely advantageous descriptor for the construction of models to stereoselectivity by Brønsted acid organocatalysts. Through statistical analyses of 50+ data sets across nearly 200 distinct catalysts, we realize active site-based buried volume is an exceptional representation, accommodating both extensive structural diversity (within data sets) and functional diversity (across data sets). We show that the descriptor’s value likely relates to its surprising capacity to generalize beyond simple steric interactions and account for diverse stereoelectronic effects consequential to reported stereoselectivity. As such, we anticipate future modeling of stereoselectivity by Brønsted acid is greatly simplified via the concise representation, active site-based buried volume.

## Graphical abstract

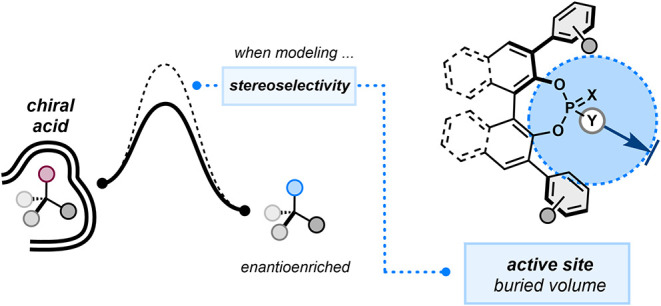

## Introduction

In chemistry, structure informs function is a paradigm invoked to justify the emergence of structure–activity relationships (SARs). In theory, chemical properties are paired with structural changes made and inferences are drawn to rationalize the emergent patterns with reaction outcomes. To build quantitative relationships, molecular representations are used as surrogates for chemical properties (Figure 1A).^1^ While many traditional SARs are constructed with representations rooted in specific experimental phenomena (e.g., Hammett,^2^ Taft,^3^ Charton^4^), such representations display narrow domains of applicability^5^—culminating in critical discontinuity failures where certain molecules of interest cannot be described by the model altogether. To address this, modern SARs are typically constructed from calculated representations (e.g., sterimol,^6^ NBO partial charge,^7^ etc.) that are intrinsically more generalizable.^8–10^ But, as molecular representations become more abstract, their subsequent interpretations become increasingly unintuitive, limiting the production of useful and general chemical rationale.^11,12^

**Figure 1:**
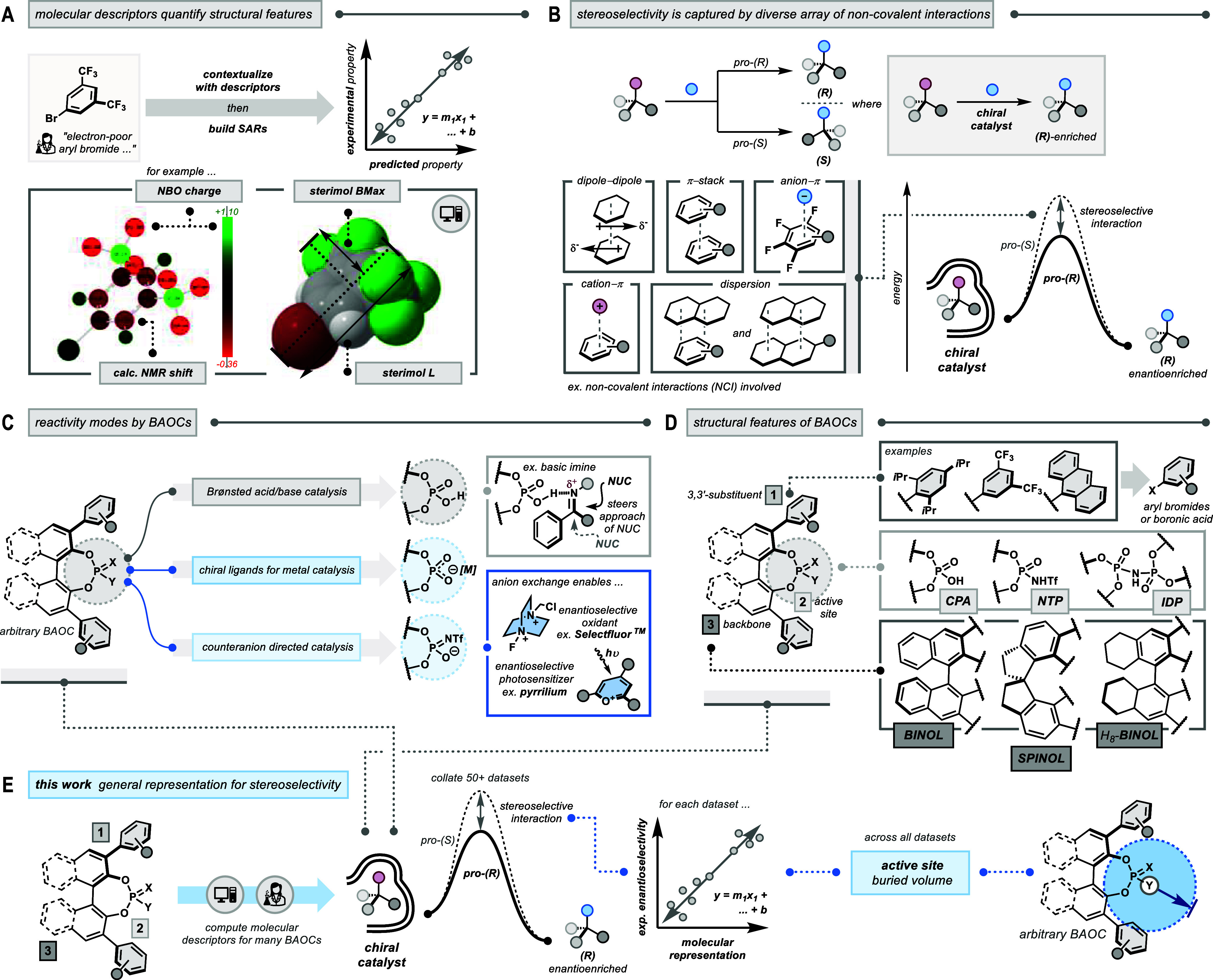
Bronsted acid organocatalysts (BAOCs) navigate an array of noncovalent interactions (NCIs) to furnish stereoselective outcomes across manifold reaction paradigms. (A) Molecular descriptors are quantitative representations of chemical structures and are readily deployed to construct structure–activity relationships (SARs). (B) Stereoselective catalysts favor the formation of one stereoisomer over another via the collective impact of many diastereoselective NCIs. (C) Access a wide range of reactivity modes. (D) BAOCs boast enormous structural tunability over the chiral scaffold surrounding the Brønsted acid active site. (E) This work—active site-based buried volume emerges as a powerful representation for stereoselectivity by BAOCs from statistical analyses of univariate SARs across many reaction data sets.

The construction of intuitive SARs is essential to understanding complex chemical phenomena. In synthetic chemistry, stereoselective catalysis is a paradigm of complexity. Here, a chiral catalyst specifically engages in an array of weak diastereoselective interactions to kinetically steer reactive intermediates toward one stereoisomer over another (Figure 1B).^1,13,14^ Notably, Brønsted acid organocatalysts (BAOCs) are one versatile tool popularized over the past two decades, enabling a wide array of applications, including: Brønsted acid/base catalysis,^15,16^ cooperative transition metal catalysis,^17^ and counteranion directed catalysis,^18^ among others (Figure 1C). Their functional diversity arises from the pronounced structural modularity of BAOC scaffolds (Figure 1D).^19,20^ To navigate this structural and functional dimensionality, synthetic chemists have made considerable progress engineering stereoselective BAOCs from two criteria—acidity^15,21^ and confinement.^22,23^ These properties are routinely qualitatively invoked, but their quantitative molecular representations have lacked general coherence and interpretability.

Seminal reports by Cavallo,^24,25^ Denmark,^26^ List,^27^ Reid,^28^ and Sigman^29^ have demonstrated that calculated representations of confinement by BAOCs can help to construct SARs for stereoselectivity. However, these models have relied on unintuitive, bespoke representations for confinement, are complicated by excessive dimensionality (e.g., too many other descriptors in the model), or are evaluated on only a very narrow set(s) of reactions. Although confinement reflects a powerful qualitative criterion for the construction of stereoselective BAOCs, a precise molecular representation, relevant across the breadth of their utility, has yet to be realized.

We herein report a powerful representation for the stereoselectivity by BAOCs—active site-based buried volume—emerging from statistical analyses of over 50+ data sets (Figure 1E). Its superior performance was benchmarked against that of many familiar and meaningful molecular descriptors, highlighting its unique ability to correlate to stereoselectivity across many data sets, regardless of the underlying structural and functional diversity of the BAOCs considered. We show that the representation, while traditionally reflecting confinement, a steric phenomenon, likely generalizes across an array of stereoelectronic noncovalent interactions. As such, we assert the descriptor is a valuable representation for BAOCs in a broad range of contexts for future modeling endeavors.

## Results and Discussion

### Active Site-Based Buried Volume Emerges as an Advantageous Representation

Stereoselective catalysis relies on an array of weak, but specific, noncovalent interactions (NCIs) between a chiral catalyst and a substrate. Accordingly, a robust representation for stereoselective catalysts necessarily (1) accommodates the structural diversity of the deployed chiral catalysts, (2) generalizes across the accessible functional manifolds, and (3) mediates mechanistic differences observed from each transformation.

In the case of BAOCs, a range of molecular descriptors have emerged as promising candidates, as reported in SARs by Cavallo,^25^ Denmark, Reid,^28^ Sigman,^9^ and our own group.^29^ Qualitatively, these descriptors aim to reflect confinement of the BAOC’s active site (vide supra) through various geometric and steric representations, including: bond length, angle, sterimol,^30^ and buried volume^24^ (Figure 2A). Beyond representations of confinement, many reported models also invoke stereoelectronic contributions to stereoselectivity from adjacent modular substituents. We account for these expectations by considering an array of substituent-based features calculated from the analogous aryl bromide as a surrogate (Figure 2A).^31^ Finally, as the acidity of BAOCs is often invoked to contextualize the differences across a series of chiral acids, we include calculated p*K*_a_ to reflect this expectation (see Molecular Descriptor Calculation Workflow for full details; see data set_active-site.csv and data set_substituent.csv for full list of computed descriptors used in this work).^32^

**Figure 2:**
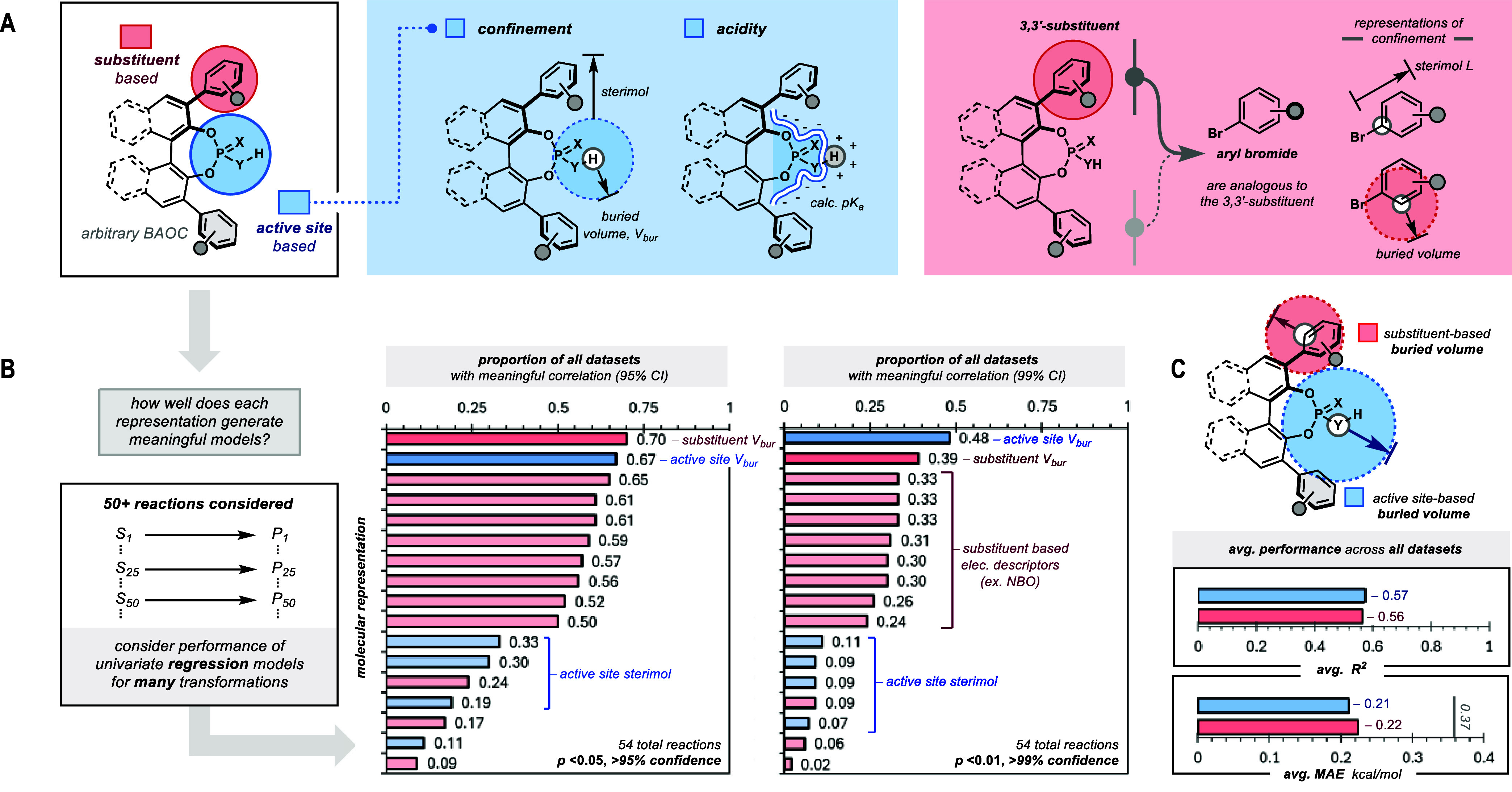
Collation of candidate molecular descriptors and detection of a general representation for stereoselectivity. (A) Active site-based representations reflect existing synthetic design criteria (e.g., acidity, confinement) without specific attention to the underlying scaffold, while substituent-based representations are derived from simplified analogs (aryl bromides). (B) Proportion of meaningful univariate regression models constructed by each descriptor at increasingly strict confidence intervals (up to 99% confidence) across the set of 50+ reaction data sets considered in this work. (C) On average, active site-based and substituent-based buried volumes construct the most *accurate* and *robust* univariate regression models (in comparison, dummy predictor of *mean* for each data set furnishes an average MAE of 0.37 kcal/mol).

Equipped with an array of candidates for general representations of BAOCs, we then collated a set of 50+ reaction data sets from the literature, culminating in nearly 200 unique BAOCs modeled (see data set_rxn.xlsx for a full list of curated data sets). To ensure the impact of any representation considered is clearly detected, univariate linear regression models trained on each reaction data set serve as the basis for analysis, avoiding data-intensive, high-dimensional models (e.g., neural networks, random forest, multivariate linear regression, etc.) that readily overfit on smaller data sets and become increasingly challenging to interpret (avg. data set size in this work—11).^12,33^ To compare the overall performance of each representation, we evaluated regression accuracy (*R* ^2^, MAE—mean absolute error) and robustness (*p*-value, LOO *R* ^2^—leave-one-out cross-validation) across each data set.^34^ For representations spanning multiple atoms, dimensions, and ensembles (e.g., partial charge at different atoms, buried volume across different radii), only the most robust regression model (largest LOO *R* ^2^ across the set) for a given data set was considered in further analyses (see data set_scores.xlsx for complete statistical analyses).

Across the 50+ reaction data sets analyzed, active site-based buried volume uniquely emerged as the most accurate and robust representation (67% of all models are statistically meaningful with >95% confidence, 48% with 99% confidence, avg. *R* ^2^ = 0.57, avg. MAE = 0.21; Figure 2B,C). At the strictest confidence shown, the average descriptor only constructed meaningful univariate models for one-fifth of the data sets (avg. 22% with >99% confidence). Surprisingly, other active site-based descriptors like sterimol L and BMin, which were expected to convey confinement like active site-based buried volume, are among the last-ranked descriptors with very sparse utility (avg. 9% with >99% confidence). Instead, the next highest ranked descriptor is substituent-based buried volume (39% with >99% confidence; avg. *R* ^2^ = 0.56, avg. MAE = 0.22). The descriptor has been deployed to represent confinement akin to its active site-based analog but apparently cannot correlate to enantioselectivity as generally across the data sets (Figure 2B; see Detailed Statistical Analyses; see Figures S21–S74 for a complete list of univariate regression models by active site-based buried volume).

### Active Site-Based Buried Volume Accommodates Structural Heterogeneity

Buried volume captures the largest proportion of meaningful univariate models relative to other descriptors, but its position—either active site-based or substituent-based—influences its utility. The juxtaposition between the two suggests that the better-performing active site-based representation conveys salient structural changes in a more general manner. It was anticipated that active site-based buried volume will advantageously correlate to stereoselectivity when the reaction data sets are constructed from structurally diverse sets of BAOCs, while substituent-based buried volume should generate robust correlations when the data sets are dominated by changes to the modular substituent. So, we organized the data sets by their corresponding structural diversity (Figure 3A; see Detailed Statistical Analyses for classification criteria). Twenty-seven of the 54 reactions considered can be classified as limited diversity; essentially, the data set has one or fewer examples of catalysts differing beyond the modular substituent (e.g., different chiral backbones and/or active sites). The remaining 27 reactions are classified as significant diversity.

**Figure 3:**
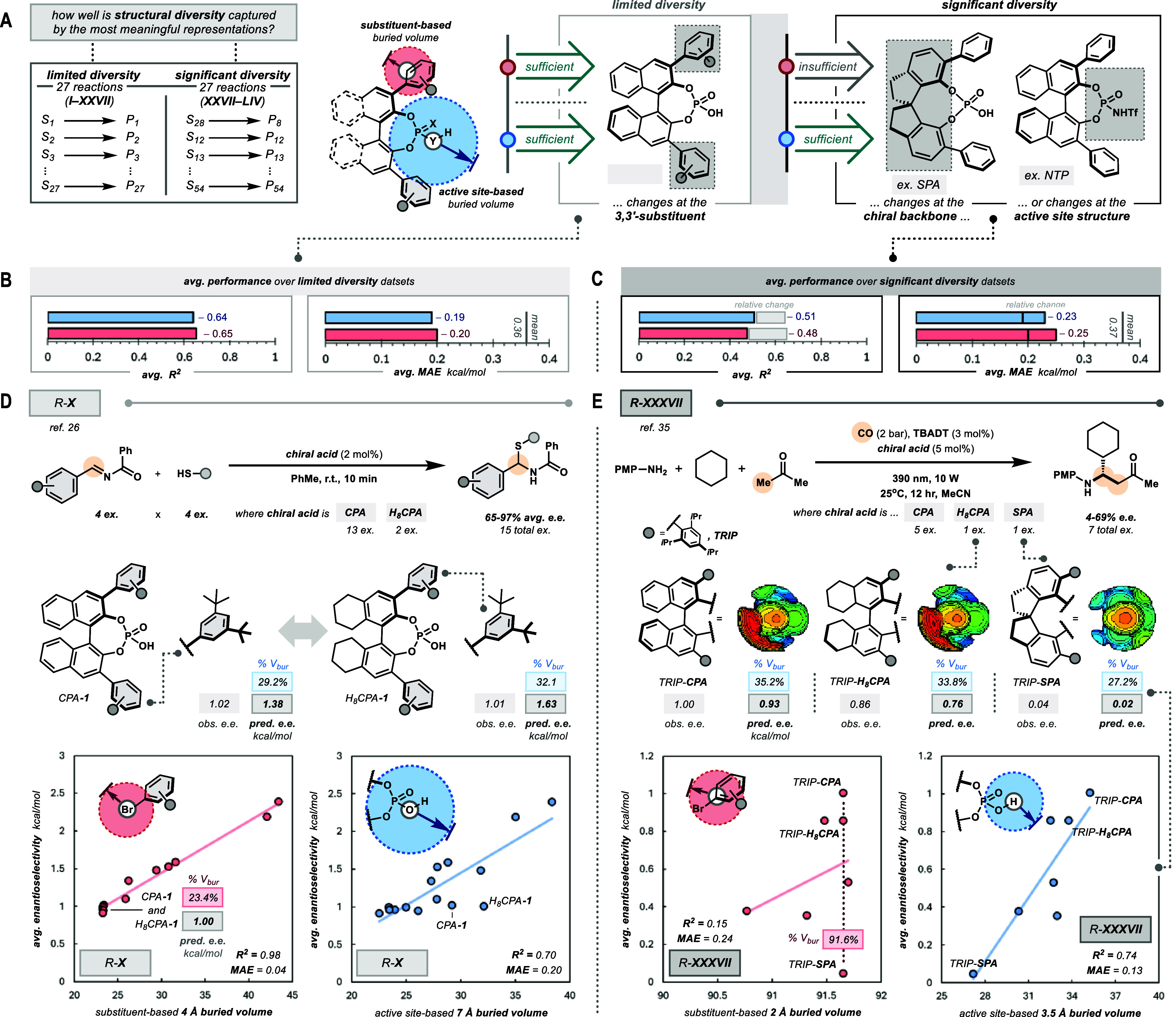
Active site-based buried volume accommodates substantial structural diversity within data sets. (A) Organizing the reaction data sets with respect to the structural diversity of the BAOCs will help to qualify the generality of candidate representations. (B) Limited diversity data sets showcase both active site-based and substituent-based buried volumes are comparable, while (C) significant diversity data sets showcase active site-based buried volume is comparatively more robust. (D) R-**X** from Zahrt et al.^26^ highlights how both active site-based and substituent-based buried volumes furnish strong trends in a limited diversity data set. (E) R-**XXXVII** from Ding et al.^35^ highlights how active site-based buried volume can advantageously furnish trends across diverse data sets.

Over the limited diversity data sets, active site-based and substituent-based buried volumes both perform well. As these data sets are populated with BAOCs varying primarily at the modular substituent, the substituent-based representation more precisely reflects the chemical changes (avg. *R* ^2^ = 0.65, avg. MAE = 0.19 kcal/mol; Figure 3B), while the active site-based representation performs at a similar caliber (avg. *R* ^2^ = 0.64, avg. MAE = 0.19 kcal/mol). On the other hand, across significant diversity data sets, active site-based buried volume outperforms its substituent-based analog. The heterogeneity of the data sets requires the descriptors to quantitatively reflect structural changes across the acids. As such, the utility of substituent-based buried volume substantially decreases (avg. *R* ^2^ = 0.47, avg. MAE = 0.25 kcal/mol; Figure 3C), while active site-based buried volume is relatively sustained (avg. *R* ^2^ = 0.51, avg. MAE = 0.23 kcal/mol).

To highlight the impact structural heterogeneity, we showcase the utility of active site-based and substituent-based buried volume in two reaction data sets. R-**X**, a seminal data set reported by Zahrt et al.^26^ describes the stereoselective addition of a thiol to an imine (Figure 3D). In this work, we model the average performance of a given BAOC across the 16 combinations of substrates reported by Zahrt et al. Across the BAOCs considered in the data set, only two examples are constructed from distinct chiral backbones (13% of the data set). Accordingly, as most structural changes are concentrated at the modular substituent, substituent-based buried volume perfectly recapitulates the reported stereoselectivity (*R* ^2^ = 0.98, MAE = 0.04 kcal/mol). Even though the active site-based buried volume cannot reflect the chemical changes with such precision, a useful univariate regression is still observed (*R* ^2^ = 0.70, MAE = 0.20 kcal/mol). Notably, other representations of confinement, such as sterimol, which have been included in multivariate SARs modeling this data set, fail to produce univariate relationships (e.g., with active site-based sterimol_L *R* ^2^ = 0.09, MAE = 0.33 kcal/mol).

Unlike the homogeneous data set of R-**X**, the data set R-**XXXVII** ^35^ describes the application of a small, but diverse array of BAOCs in a multicomponent photochemical transformation (Figure 3E). Critically, it was observed in the original report that the choice of chiral backbone significantly altered stereoselectivity (ref TRIP-**CPA**, TRIP-**H**_**8**_ **CPA**, TRIP-**SPA** in Figure 3E). The trend drawn by *active site-based* buried volume seamlessly captures the enantioinduction cooperatively afforded by the chiral backbone and the modular substituents (*R* ^2^ = 0.74, MAE = 0.13 kcal/mol). Of course, substituent-based buried volume cannot reflect such structural changes over the data set, so it fails to correlate (*R* ^2^ = 0.15, MAE = 0.24 kcal/mol; Figures S7–S9).

### Active Site-Based Buried Volume Retains Utility across Catalytic Manifolds

The success of active site-based buried volume across significant diversity data sets supports the expectation that the descriptor will convey the collective consequences of each individual structural feature on stereoinduction. However, it is unclear whether such performance is also evidence of the descriptor’s sustained utility across all BAOC reactivity modes. Traditionally, BAOCs were deployed in acid-catalyzed additions to π-bonds (e.g., imine additions).^16^ Yet, modern applications of BAOCs have expanded their functional arsenal beyond additions to include stereoselective eliminations and substitutions under a range of conditions (e.g., thermal, phase-transfer,^36^ and photochemical). If active site-based buried volume is truly general, then its utility must be demonstrated across the array of reaction manifolds accessible to BAOCs.

To investigate this, we partitioned the 50+ reaction data sets into three distinct classes corresponding to catalytic activity: (1) acid-mediated additions, (2) acid-mediated substitutions and eliminations, and (3) complex, multicomponent paradigms (Figure 4A). Indeed, across each of these categories, active site-based buried volume furnishes accurate and robust regressions (addition – avg. *R* ^2^ = 0.56, elimination and substitution – avg. *R* ^2^ = 0.63, and complex – avg. *R* ^2^ = 0.52; Figure 4B). Other alternative representations of confinement, like sterimol, which have previously reported utility for specific acid-mediated additions, fail to accommodate the remaining functional diversity available to the BAOCs (e.g., with sterimol_L addition – avg. *R* ^2^ = 0.23, elimination and substitution – avg. *R* ^2^ = 0.28, and complex – avg. *R* ^2^ = 0.21; Figures S10–S13 for full analyses to other representations of confinement).

**Figure 4:**
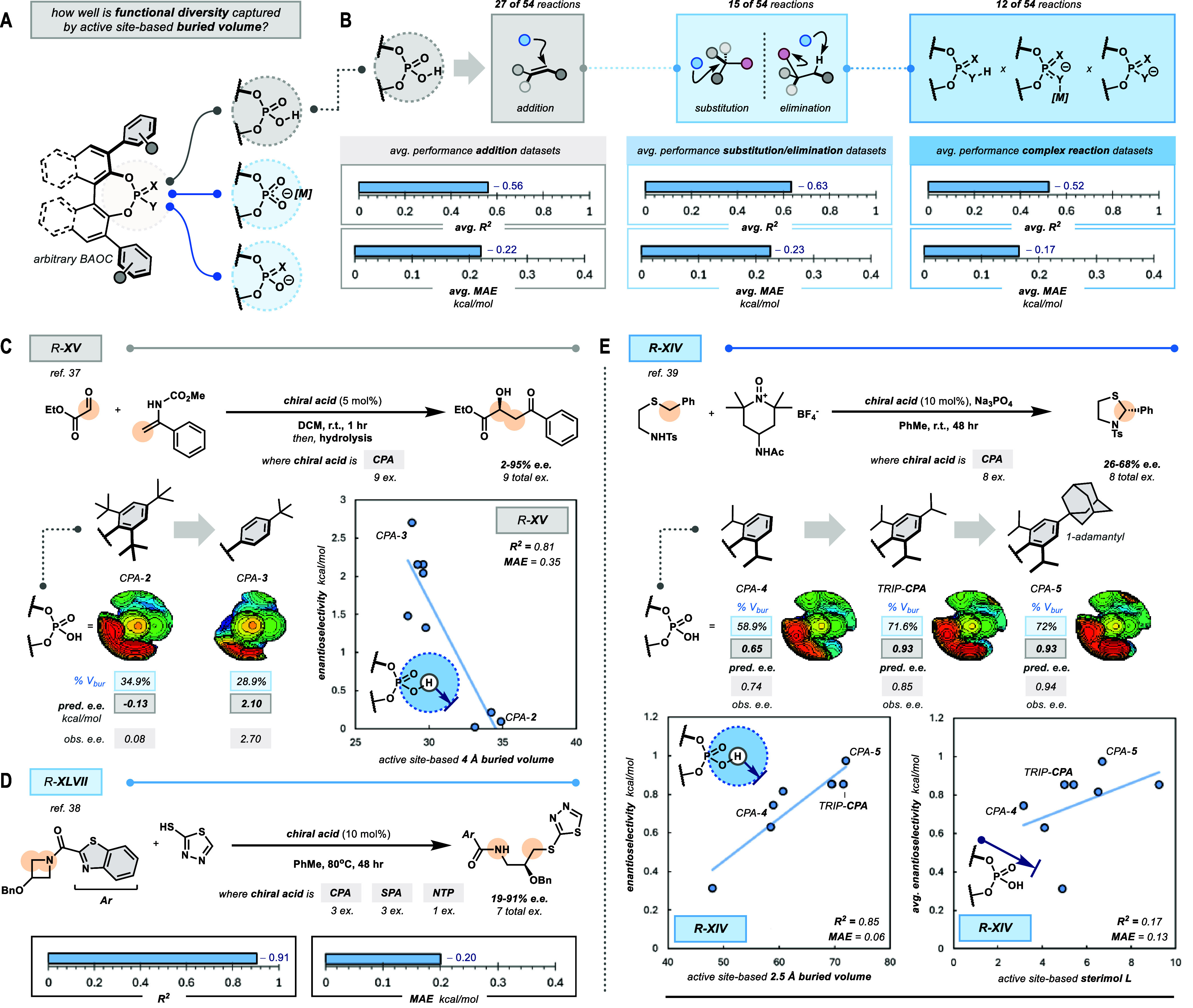
Active site-based buried volume retains utility across BAOC reaction manifolds. (A) BAOC reactivity can be organized into three categories reflecting the role of the acid, where (B) active site-based buried volume continues to furnish *accurate* trends across each category. (C) R-**XV** by Terada et al.^37^ highlights how active site-based buried volume can reflect counterintuitive relationships for stereoselective additions under confinement. (D) R-**XLVII** by Wang et al.^38^ highlights how active site-based buried volume can accommodate substantial structural diversity, even within a stereoselective substitution. (E) R-**XIV** by Toste et al.^39^ highlights how *active site-based* buried volume serves as a privileged representation for stereoselectivity by BAOCs, even within complex, multicomponent transformations like phase-transfer catalysis.

To highlight the consistent utility of the active site-based buried volume across reaction manifolds, we showcase one reaction from each category enumerated above. While many acid-mediated additions rely on basic functional groups (e.g., imines) to strongly bind to the chiral acid active site, stereoselective solutions have emerged for substrates lacking such motifs. One early example of this is R-**XV**, a stereoselective aldol-like addition between an enamide and a glyoxal reported by Terada and co-workers (Figure 4C).^37^ The report realized that traditional stereoselective intuition—increasing confinement—does not furnish the desired adduct with selectivity (ref CPA-**2** with 8% e.e.). Instead, reducing confinement at the active site was required to achieve the desired alcohol with high selectivity (ref CPA-**3** with 98% e.e.). Active site-based buried volume reflects Terada’s counterintuitive findings, furnishing a useful univariate regression model (*R* ^2^ = 0.81, MAE = 0.35 kcal/mol).

Beyond acid-mediated additions, many stereoselective eliminations (e.g., atroposelective condensations) and substitutions mediated by BAOCs have been reported. Here, we highlight R-**XLVII**, an intermolecular substitution of azetidine with thiol nucleophiles (Figure 4D).^38^ The authors screen a small (e.g., 7) but diverse (3 distinct scaffolds) set of BAOCs to realize a solution (up to 91% e.e.). Active site-based buried volume not only accommodates the structural heterogeneity of the data set, but also the BAOC-mediated substitution, yielding an excellent linear model (*R* ^2^ = 0.91, MAE = 0.20 kcal/mol).

Finally, though BAOCs are typically employed as chiral acids, their applications have evolved beyond the scope of simple acid/base chemistry to include applications as ligands in asymmetric transition metal catalysis and chiral counterions (e.g., asymmetric counterion-directed catalysis, ACDC). For years, our group has investigated stereoselective phase-transfer oxidations mediated by chiral phosphates. One notable example from our group describes a phase-transfer-catalyzed Pummerer-type cyclization that required remote structural changes to enhance the observed stereoselectivity (R-**XIV**, Figure 4E).^39^ In our original report, a multivariate regression from a collection of sterimol descriptors was required to reflect the impact of the distant structural changes to stereoselectivity (three terms, *R* ^2^ = 0.96). Here, we show that active site-based buried volume alone readily recapitulates the relationship (one term, *R* ^2^ = 0.85, MAE = 0.06, Figure 4E). In comparison, active site-based sterimol features are unsuitable for univariate relationships (e.g., with sterimol L alone, *R* ^2^ = 0.17, MAE = 0.13, Figure 4E).

### Active Site-Based Buried Volume Holistically Generalizes Stereoelectronic Effects

Across the 50+ reaction data sets considered herein, active site-based buried volume uniquely reflects confinement as a quantitative representation for stereoselectivity by BAOCs, regardless of the underlying structure and function. Yet, confinement is often considered a steric phenomenon, where models for stereoselectivity invoke steric repulsion as the primary destabilizing criterion. More recently, chemists prefer to invoke attractive noncovalent interactions as a primary design criterion for stereoselectivity. From this perspective, confinement qualitatively reflects a propensity for attractive dispersion forces.^22,40,41^ Still, a preponderance of other stereoelectronic noncovalent interactions (NCIs) have proven relevant for stereoselective catalysis (e.g., π–π, cation–π, etc., Figure 5A).^13,14,42,43^ Is active site-based buried volume capable of reflecting such diverse stereoelectronic NCIs?

**Figure 5:**
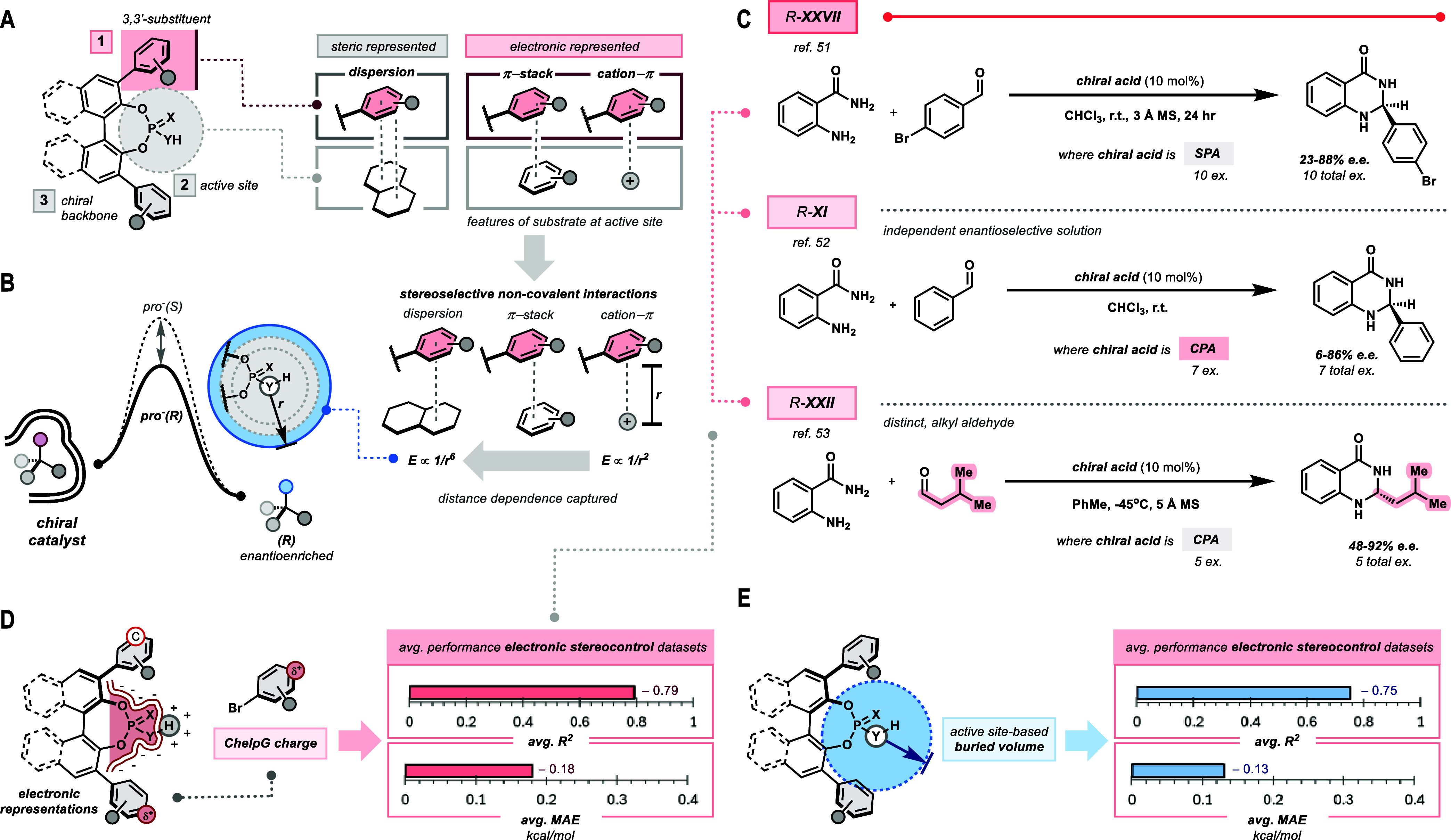
Active site-based buried volume holistically generalizes stereoelectronic effects across reaction data sets considered. (A) Stereoselective NCIs can be categorized as predominantly steric or electronic-mediated. (B) Interaction energies of various stereoselective NCIs relate to distance (denoted *r*) which is reflected by the *active site-based* buried volume. (C) R-**XXVII** is a data set reported by Wheeler et al.^51^ to operate via electronic stereocontrol. R-**XI** ^52^ and R-**XXII** ^53^ transformations likely operate similarly by analogy to R-**XXVII**. (D) Electronic representations like substituent-based ChelpG charge furnish excellent trends (averaged across R-**XXVII**, R-**XI**, and R-**XXII**). (E) Active site-based buried volume also furnishes excellent univariate trends (averaged across R-**XXVII**, R-**XI**, and R-**XXII**).

In theory, electronic contributions to NCIs (e.g., cation–π or π–π interactions) are defined from the interaction of charges across a distance (e.g., ion-dipole scales with 1/*r* ^2^, where *r* represents the distance between charges, van der Waals attraction scales with 1/*r* ^6^, Figure 5B).^1,44^ It follows that active site-based buried volume intimately relates percent steric occupancy across a specific distance (lit. volume) should formally capture such spatial interactions through this relationship. Additionally, it is generally observed that larger π-systems (readily reflected by buried volume) access stronger electrostatic NCIs (e.g., cation–π) as a consequence of their greater polarizability.^44–47^ In fact, our group has previously shown robust, univariate relationships exist between buried volume and the strength of corresponding, stereoselective cation–π NCIs in some putative, stereodetermining reaction complexes.^48,49^ As highlighted in R-**XV** above, active site-based buried volume reflects stereoselectivity for a phase-transfer catalyst where stereoinduction was attributed to strengthening C–H–π interactions in the major transition state.^39^ Thus, across the data sets considered, active site-based buried volume likely reflects an array of stereoselective interactions by BAOCs, including those that are principally electronic in nature.

To ensure that such examples are not merely instances of *cooperative* steric and electronic control, we turned to the literature to identify BAOC-mediated transformations where stereoselectivity was explicitly realized via *electronic* NCIs. Indeed, elucidated with extensive computational work, Wheeler and co-workers have realized that such electronic control was observed in data set R-**XXVII**, a stereoselective condensation furnishing cyclic aminals, whereby biased Coulombic interactions across the highly polarized active site drive stereoselectivity (Figure 5C).^50,51^ Notably, this condensation is analogous to two other condensations analyzed herein: R-**XI** and R-**XXII**. As expected, substituent-based electronic representations like ChelpG charge furnish meaningful univariate trends across the collection of transformation operating via electronic control (up to *R* ^2^ = 0.79, MAE = 0.18 kcal/mol; Figure 5D, see Figures S15–S20 for remaining electronic representations). Active site-based buried volume similarly recapitulates these trends (*R* ^2^ = 0.75, MAE = 0.13 kcal/mol; Figure 5E), supporting the expectation that it can reflect principally electronic, stereoselective NCIs.

## Conclusion

We have demonstrated that active site-based buried volume is a uniquely advantageous descriptor for the construction of simple and holistic relationships to stereoselectivity by BAOCs. These catalysts alter stereoinduction through an array of diverse NCIs collectively conferred by many distinct structural elements. As such, there is not one universally shared mechanistic attribute specifically exploited by the most selective catalysts. Still, active site-based buried volume navigates this stereoselective heterogeneity of each reaction data set by standardizing spatial interactions, even across structurally diverse BAOCs. Inspired by these findings, we are currently investigating the application of an active site-based buried volume to inform and guide the construction of stereoselective solutions in BAOC-mediated transformations. More broadly, intentional investigation of general chemical representation for complex functions (e.g., stereoselectivity) across any chemistry may reveal surprisingly simple and intuitive relationships.

## Supplementary Material











## Notes

Active site-based descriptors (CSV)
Reaction data sets (XLSX)
All regression analyses for each descriptor (XLSX)
Substituent-based descriptors (CSV)
General calculation details and statistical analyses (PDF)

## Abbreviations

BAOC: Brønsted acid organocatalyst
NCI: noncovalent interaction
SAR: structure–activity relationship
