# Supplementary material for: Stereoselective Generalizations over Diverse Sets of Chiral Acids Enabled by Buried Volume
Source: J Am Chem Soc. 2026 Jan 6;148(2):2792–800. doi: 10.1021/jacs.5c20342 (PMC12833812; doi:10.1021/jacs.5c20342)
Supplement: Supplementary file 5 [file ja5c20342_si_005.pdf]

Supporting Information

for

Stereoselective generalizations over diverse sets of chiral acids enabled by buried volume

Andrew L. Smith, F. Dean Toste

Department of Chemistry, University of California, Berkeley 94720

Corresponding author: fdtoste@berkeley.edu

This file includes:

Computational Methods

Descriptor Calculation Workflow

Detailed Statistical Analyses

Other supplementary materials:

Datasets for reported reaction datasets (stereoselectivity in kcal/mol) used in statistical analyses.

dataset_rxn.xlsx [1–51]

Descriptors calculated for chiral acid organocatalysts considered herein.

dataset_active-site.csv

Descriptors calculated for modular substituents, represented by corresponding aryl bromide.

dataset_substituent.csv

Complete statistical analyses (R^2, MAE, p-value, LOO-R^2) across all descriptors considered.

dataset_scores.xlsx

Table of Contents

Computational Methods **3**

Molecular Descriptor Calculation Workflow **4**

Substituent-based descriptor generation – *dataset_substituent.csv* 4

Active site-based descriptor generation – *dataset_active-site.csv* 5

Visualizations of Buried Volume with SambVca 2.1 11

Detailed Statistical Analyses **19**

Expanded Confidence Intervals for Meaningful Correlations 19

Regression Performance over Structurally Diverse Datasets 23

Regression Performance over Functionally Diverse Datasets 28

Regression Performance over Electronic-Controlled Datasets 32

Univariate Regression Models from Active Site-based Buried Volume 39

References **57**

Computational Methods

General Calculation Details

Calculations were remotely conducted using computing clusters at the Molecular Graphics and Computation Facility (MGCF) at the University of California, Berkeley (NIH S10OD034382). Semiempirical quantum mechanical method by Grimme's extended tight binding (xTB) package 3 was used (v6.6.1).[52] Conformational searching was conducted with Grimme's conformer-rotamer ensemble sampling tool, CREST (v2.12).[53] Higher level quantum mechanics-based descriptors were computed and extracted following Gaussian16 single point energy (B3LYP/6-31+(d) level of theory with the keywords: emp=gd3 nmr=giao pop=orbitals=1 pop=npa pop=(chelpg, readradii) pop=Hirshfeld scf=xqc).[54] These calculations were conducted on the geometries of conformers produced by CREST and xTB. In-house Python scripts were powered by a personal, RDKit conda environment (Python == 3.10.12, rdkit == 2023.09.2).[55] Plots of correlations and assorted visualizations were generated using Microsoft Excel. Visualizations of *active site-based* buried volume were generated using SambVca 2.1.[56]

General Statistical Methods

In this report, we compiled 54 reactions from literature reports (ref. *dataset_rxn.xlsx*). To accommodate comparisons between *substituent*-based (corresponding to the aryl bromide surrogates, *dataset_substituent.csv*) and *active site*-based (*dataset_active-site.csv*) descriptors, **all datasets were restricted to 3,3'-aryl substituted BAOCs**. Literature reported transformations including heteroatom or alkyl substituents were discarded from the analysis. Only the data tabulated in *dataset_rxn.xlsx* were considered in the analyses reported.

The small nature of each reaction dataset (majority are 10 or fewer data points) will necessarily result in overfitting of models with too high dimensionality. To avoid this altogether, **we exclusively deploy univariate linear regression models to build correlations** between reported enantioselectivity (in kcal/mol) and the corresponding molecular representation. We do not aim to draw conclusions with respect to the specific atomic identity of each descriptor nor to the specific ensemble. Therefore, we pool our analyses drawn across sets of atoms and ensembles that are shared by descriptors (ex. buried volumes from 2-7 Å are individually considered when building models, but the best performing across all calculated buried volumes is recorded for the specific dataset). This results in a collection of 20 *unique sets* of descriptors where 5 are *active site*-based and 15 are *substituent*-based. The full results of univariate linear regressions drawn by from each unique set of descriptors is tabulated in the *dataset_scores*.xlsx supplementary file. The univariate linear regressions are scored with respect to the correlation drawn from the full dataset, including: R^2, mean absolute error (MAE), p-value, and LOO-R^2. Most metrics were extracted using *sklearn's* built-in modules (ex. *sklearn.metrics.mean_absolute_error* and *sklearn.model_selection.KFold*).

Molecular Descriptor Calculation Workflow

The molecular descriptors reported in the Supplementary Materials (*dataset_substituent.csv* and *dataset_active-site.csv*, corresponding to the computed aryl bromides and chiral acid organocatalysts, respectively) were computed and collated in an analogous manner to that previously reported by our group in Treacy, S. et al. *J. Am. Chem. Soc.* **2024.**[57]

To highlight the key steps, the workflow generally operates by (1) generating an initial conformer for each molecule considered, (2) optimizing the structure to a local minimum with Grimme's xTB, (3) searching for accessible conformers with Grimme's CREST within a 1.8 kcal/mol window (corresponding to approximately 95% of accessible conformers at 298 K), (4) additional single-point calculations across the conformer ensemble, and (5) extraction and export of ensemble-level descriptors for each molecule into *csv* files (ref. above *datasets*).

Substituent-based descriptor generation – *dataset_substituent.csv*

The specific calculation workflow to generate aryl bromide (substituent-based) descriptors operates by (1) producing an initial geometry from SMILES strings with RDKit's *AllChem* module (random seed = *0xf00d*), (2) optimization of the initial geometry with Grimme's xTB, (3) searching for accessible conformers with Grimme's CREST within a 1.8 kcal/mol window, (4) additional single-point energy calculations with Gaussian16 at the B3LYP/6-31+(d) level of theory using the following additional keywords (emp = gd3 nmr = giao pop=orbitals=1 pop=npa pop=(chelpg, readradii) pop=Hirshfeld scf=xqc) where the van der Waals radii for each element is implemented as in DBSTEP package by Luchini, G. Paton, R. et al. **2022.**,[58] and (5) extraction and export of ensemble-level descriptors for each aryl bromide into *csv* files. A numerical index is assigned to each processed SMILES string and tabulated in *dataset_substituent.csv*.

#1 – Generating initial conformations for each aryl bromide

The aryl bromide geometries were instantiated as described above. The selected SMILES strings were inspired by the reported aryl bromide dataset by Kariofillis, S. Doyle, A. et al. *J. Am. Chem. Soc.* **2022.**[59] and augmented with additional aryl bromides as needed from the collated datasets (ref. *dataset_rxn.xlsx*)

#5 – Extracting and export ensemble-level descriptors

The calculated representations for each aryl bromide are shown in *dataset_substituent.csv*. The ensembles computed for each aryl bromide are tagged at the end of each descriptor, including: (1) xTB-based ground state (written as 'C_0'), (2) DFT-based ground state (written as 'GD'), (3) xTB-based Boltzmann average (written as 'AV_XTB'), (4) DFT-based Boltzmann average (written as 'AV_DFT'), (5) minimum value across all conformers (written as 'MIN'), and (6) maximum value across all conformers (written as 'MAX').

Active site-based descriptor generation – *dataset_active-site.csv*

The specific calculation workflow to generate BAOC (active site-based) descriptors operates by (1) producing an initial geometry from the preset 3D-configuration of the following chiral scaffolds, (2) optimization of the initial geometry with Grimme's xTB, (3) searching for accessible conformers with Grimme's CREST within a 1.8 kcal/mol window, and (4) extraction and export of ensemble-level descriptors for each BAOC into *csv* files, where **each molecule is labeled BAOC_NUM (BAOC – the scaffold used, NUM – the substituent analogous to the corresponding aryl bromide in *dataset_substituent.csv*).**

#1 – Generating initial conformations for each BAOC

The BAOC geometries were instantiated using an in-house Python script that substitutes a placeholder atom (labeled 'Bq') with the xTB-based ground state conformation of the corresponding aryl bromide to produce the desired 3,3'-substituted BAOC. It should be noted that instantiation by SMILES with RDKit's *AllChem* module does not conserve the axial chirality present in the chiral backbone of the BAOCs considered in this work.

The following coordinates (*templates*) were used as the initial geometries for each BAOC prior to substitution with the corresponding aryl bromide. Each *template* is labeled BAOC_H or BAOC_CB corresponding to the acid or conjugate base of the 'BAOC' scaffold.

CPA_H

C	0.88872103	0.21932295	-0.06941144	C	-1.75259838	0.44928712	5.90668080
C	0.84438745	-1.04602043	-0.56397060	C	-0.61070021	1.03556500	5.45993875
C	0.15635054	-2.05673797	0.12984923	H	-2.10526226	-2.21656835	2.20807746
C	-0.41251901	-1.79256914	1.37864309	C	-2.48717322	-1.94010194	3.16863953
C	-0.28528609	-0.51846150	1.93665607	C	-3.49669410	-1.22925601	5.66006057
C	0.25442852	0.51934442	1.14338424	H	-2.18183698	0.76055103	6.83607351
H	0.47488569	-3.55143998	-1.37579900	C	-4.08902661	-2.20949537	4.93147238
H	1.32972158	-1.27241451	-1.49031170	C	-3.57888180	-2.56879471	3.67246164
C	0.04129354	-3.33852158	-0.42104807	H	-3.89267319	-0.96039765	6.61700800
C	-1.08838666	-2.80835741	2.06365319	H	-4.95071379	-2.71207311	5.31853144
C	-1.19153804	-4.04337078	1.51153934	H	-4.05451921	-3.34294996	3.10737478
C	-0.62046008	-4.31131051	0.25600640	O	0.09825209	1.88279642	1.56881937
H	-1.52313530	-2.61148652	3.02131728	O	1.26734287	1.07578404	3.86519640
H	-1.71083123	-4.81831535	2.03566391	P	1.22400167	2.32929139	2.74037692
H	-0.70772198	-5.28923769	-0.16934927	O	0.77988403	3.66284025	3.40816851
C	-0.04547529	0.63091660	4.24351296	O	2.77592537	2.53955076	2.05377380
C	-0.74409242	-0.26246005	3.39973966	**Bq**	**0.16213058**	**2.30997111**	**6.41467517**
C	-1.86169898	-0.92950826	3.90723706	**Bq**	**1.74728441**	**1.48735299**	**-0.95703114**
C	-2.36772027	-0.57017082	5.15938874	H	3.44641870	2.33837582	2.71071165

CPA_CB

C	0.88872103	0.21932295	-0.06941144	H	1.32972158	-1.27241451	-1.49031170
C	0.84438745	-1.04602043	-0.56397060	C	0.04129354	-3.33852158	-0.42104807
C	0.15635054	-2.05673797	0.12984923	C	-1.08838666	-2.80835741	2.06365319
C	-0.41251901	-1.79256914	1.37864309	C	-1.19153804	-4.04337078	1.51153934
C	-0.28528609	-0.51846150	1.93665607	C	-0.62046008	-4.31131051	0.25600640
C	0.25442852	0.51934442	1.14338424	H	-1.52313530	-2.61148652	3.02131728
H	0.47488569	-3.55143998	-1.37579900	H	-1.71083123	-4.81831535	2.03566391

H	-0.70772198	-5.28923769	-0.16934927
C	-0.04547529	0.63091660	4.24351296
C	-0.74409242	-0.26246005	3.39973966
C	-1.86169898	-0.92950826	3.90723706
C	-2.36772027	-0.57017082	5.15938874
C	-1.75259838	0.44928712	5.90668080
C	-0.61070021	1.03556500	5.45993875
H	-2.10526226	-2.21656835	2.20807746
C	-2.48717322	-1.94010194	3.16863953
C	-3.49669410	-1.22925601	5.66006057
H	-2.18183698	0.76055103	6.83607351
C	-4.08902661	-2.20949537	4.93147238
C	-3.57888180	-2.56879471	3.67246164
H	-3.89267319	-0.96039765	6.61700800
H	-4.95071379	-2.71207311	5.31853144
H	-4.05451921	-3.34294996	3.10737478
O	0.09825209	1.88279642	1.56881937
O	1.26734287	1.07578404	3.86519640
P	1.22400167	2.32929139	2.74037692
O	0.77988403	3.66284025	3.40816851
O	2.77592537	2.53955076	2.05377380
**Bq**	**0.16213058**	**2.30997111**	**6.41467517**
**Bq**	**1.74728441**	**1.48735299**	**-0.95703114**

H8CPA_H

C	1.05675548	-0.32764938	-0.20390985
C	0.76294549	-1.47926168	-0.82687915
C	-0.15569110	-2.34191501	-0.16688326
C	-0.55989132	-2.18698660	1.14988397
C	-0.13598241	-0.99391124	1.85309706
C	0.51673754	-0.06302231	1.03233817
H	-0.26512416	-4.29669153	-0.25273780
H	1.17740304	-1.69952963	-1.79500815
C	-0.82954913	-3.47881900	-0.78116895
C	-1.49147072	-3.30669446	1.65652416
C	-2.64357698	-3.27016367	0.89572001
C	-2.37292832	-3.62145231	-0.54749713
H	-0.99251440	-4.33473126	1.73435166
H	-3.36436983	-3.99071962	1.28896536
H	-3.10210194	-3.05527600	-1.24887752
C	0.59356571	0.20673592	4.01461605
C	-0.41270430	-0.56364133	3.41023978
C	-1.58029944	-0.92787588	4.21886930
C	-1.63453958	-0.36359275	5.53994718
C	-0.70585928	0.59296811	6.00029896
C	0.37131349	0.88407670	5.25829992
H	-2.37093774	-2.58908791	3.18243573
C	-2.73568605	-1.87037185	3.72636221
C	-2.68839117	-0.72992458	6.51579344
H	-0.90661858	1.07051109	6.95692914
C	-3.93030588	-1.19095171	5.80042639
C	-3.55306900	-2.33788876	4.94966386
H	-2.29920680	-1.54374743	7.08033733
H	-4.65419109	-1.47692682	6.46226855
H	-4.42444156	-2.72765415	4.52161626
O	0.64667776	1.30490660	1.47632517
O	1.92796997	0.31573179	3.41333118
P	1.99806916	1.55701846	2.31533927
O	1.98615715	2.93854950	3.00813870
O	3.35091009	1.47270992	1.28512306
**Bq**	**1.57736296**	**2.11804104**	**5.77599746**
**Bq**	**2.08034395**	**0.89643042**	**-1.03486345**
H	4.14515012	1.45389442	1.78727855
H	-2.90030850	0.09953238	7.08895552
H	-4.27281971	-0.35456025	5.15285331
H	-2.98379683	-3.08094032	5.63985315
H	-3.46021300	-1.23841207	3.18164831
H	-1.77180955	-3.11452194	2.58348113
H	-2.97010404	-2.25874398	1.04353975
H	-2.50880093	-4.70743073	-0.65395651
H	-0.65132573	-3.55524044	-1.87310972

H8CPA_CB

C	1.05675548	-0.32764938	-0.20390985
C	0.76294549	-1.47926168	-0.82687915
C	-0.15569110	-2.34191501	-0.16688326
C	-0.55989132	-2.18698660	1.14988397
C	-0.13598241	-0.99391124	1.85309706
C	0.51673754	-0.06302231	1.03233817
H	-0.26512416	-4.29669153	-0.25273780
H	1.17740304	-1.69952963	-1.79500815
C	-0.82954913	-3.47881900	-0.78116895
C	-1.49147072	-3.30669446	1.65652416
C	-2.64357698	-3.27016367	0.89572001
C	-2.37292832	-3.62145231	-0.54749713
H	-0.99251440	-4.33473126	1.73435166
H	-3.36436983	-3.99071962	1.28896536
H	-3.10210194	-3.05527600	-1.24887752
C	0.59356571	0.20673592	4.01461605
C	-0.41270430	-0.56364133	3.41023978
C	-1.58029944	-0.92787588	4.21886930
C	-1.63453958	-0.36359275	5.53994718
C	-0.70585928	0.59296811	6.00029896
C	0.37131349	0.88407670	5.25829992
H	-2.37093774	-2.58908791	3.18243573
C	-2.73568605	-1.87037185	3.72636221
C	-2.68839117	-0.72992458	6.51579344
H	-0.90661858	1.07051109	6.95692914
C	-3.93030588	-1.19095171	5.80042639
C	-3.55306900	-2.33788876	4.94966386
H	-2.29920680	-1.54374743	7.08033733
H	-4.65419109	-1.47692682	6.46226855
H	-4.42444156	-2.72765415	4.52161626
O	0.64667776	1.30490660	1.47632517
O	1.92796997	0.31573179	3.41333118
P	1.99806916	1.55701846	2.31533927
O	1.98615715	2.93854950	3.00813870
O	3.35091009	1.47270992	1.28512306
**Bq**	**1.57736296**	**2.11804104**	**5.77599746**

**Bq**	**2.08034395**	**0.89643042**	**-1.03486345**
H	-2.90030850	0.09953238	7.08895552
H	-4.27281971	-0.35456025	5.15285331
H	-2.98379683	-3.08094032	5.63985315
H	-3.46021300	-1.23841207	3.18164831
H	-1.77180955	-3.11452194	2.58348113
H	-2.97010404	-2.25874398	1.04353975
H	-2.50880093	-4.70743073	-0.65395651
H	-0.65132573	-3.55524044	-1.87310972

SPA_H

C	0.86573410	-0.01036456	-0.71014664
C	0.10487565	-1.24513412	-0.95545406
C	-0.70368703	-1.83206320	0.12093243
C	-0.49966716	-1.36687077	1.32933725
C	0.21141474	-0.30054358	1.31385474
C	0.74464034	0.60910081	0.59005943
H	0.12550678	-1.70398870	-1.92185296
C	0.03971552	0.93081201	4.39580632
C	-0.46933428	0.04093369	3.63126643
C	-1.03938635	-1.10514976	3.56563073
C	-1.36473563	-1.55441775	4.75331014
C	-1.27945639	-0.59823783	5.86475679
C	-0.58290812	0.68360394	5.67649387
H	-1.70642798	-0.84133397	6.81528271
O	1.00056279	1.98567139	0.98967822
O	1.11349408	1.85057309	4.04785739
P	1.48985157	2.66760391	2.53593917
O	0.82296117	4.07281443	2.58163134
O	3.19476636	2.79890953	2.52478642
**Bq**	**-0.46758848**	**1.86190885**	**6.99224420**
**Bq**	**1.85607529**	**0.71982044**	**-1.98252796**
H	3.48662565	3.24452592	3.32343791
C	-1.84336661	-2.87421179	0.32109299
H	-1.44081348	-3.84940355	0.49956435
H	-2.52879775	-2.94870743	-0.49715792
C	-1.20772958	-1.99268820	2.43030199
C	-1.69690959	-3.05379402	4.49543800
H	-1.43625640	-3.71293808	5.29699212
H	-2.73934275	-3.18352095	4.29195820
C	-2.49231898	-2.24401820	1.63288756
H	-3.21844556	-2.85178577	2.13114329
H	-2.95887781	-1.31420646	1.38254181
C	-0.80166238	-3.25849468	3.19324390
H	-0.95724873	-4.17324920	2.66040523
H	0.23187620	-3.20924589	3.4657738

SPA_CB

C	0.86573410	-0.01036456	-0.71014664
C	0.10487565	-1.24513412	-0.95545406
C	-0.70368703	-1.83206320	0.12093243
C	-0.49966716	-1.36687077	1.32933725
C	0.21141474	-0.30054358	1.31385474
C	0.74464034	0.60910081	0.59005943
H	0.12550678	-1.70398870	-1.92185296
C	0.03971552	0.93081201	4.39580632
C	-0.46933428	0.04093369	3.63126643
C	-1.03938635	-1.10514976	3.56563073
C	-1.36473563	-1.55441775	4.75331014
C	-1.27945639	-0.59823783	5.86475679
C	-0.58290812	0.68360394	5.67649387
H	-1.70642798	-0.84133397	6.81528271
O	1.00056279	1.98567139	0.98967822
O	1.11349408	1.85057309	4.04785739
P	1.48985157	2.66760391	2.53593917
O	0.82296117	4.07281443	2.58163134
O	3.19476636	2.79890953	2.52478642
**Bq**	**-0.46758848**	**1.86190885**	**6.99224420**
**Bq**	**1.85607529**	**0.71982044**	**-1.98252796**
C	-1.84336661	-2.87421179	0.32109299
H	-1.44081348	-3.84940355	0.49956435
H	-2.52879775	-2.94870743	-0.49715792
C	-1.20772958	-1.99268820	2.43030199
C	-1.69690959	-3.05379402	4.49543800
H	-1.43625640	-3.71293808	5.29699212
H	-2.73934275	-3.18352095	4.29195820
C	-2.49231898	-2.24401820	1.63288756
H	-3.21844556	-2.85178577	2.13114329
H	-2.95887781	-1.31420646	1.38254181
C	-0.80166238	-3.25849468	3.19324390
H	-0.95724873	-4.17324920	2.66040523
H	0.23187620	-3.20924589	3.46577384

NTP_H

C	3.98429469	3.00088863	0.02996965
C	4.92294732	3.74336534	-0.61411701
C	5.74901175	3.15553864	-1.58786263
C	5.67506601	1.78283145	-1.83963015
C	4.77908089	0.99719130	-1.11069723
C	3.84274276	1.63941460	-0.26881273
H	6.72301518	4.99077212	-2.12227409
H	5.02882660	4.78292326	-0.38388050
C	6.65782195	3.93915065	-2.30866663
C	6.50824289	1.20736787	-2.80530576
C	7.38076394	1.98435638	-3.49519542
C	7.45632009	3.36485120	-3.24414698
H	6.45742697	0.15633404	-2.99933065
H	8.01551904	1.54304019	-4.23494272
H	8.14838613	3.96765691	-3.79421054
C	4.56003577	-1.39062882	-0.13570118
C	4.81765057	-0.55141710	-1.24354041
C	5.12145400	-1.13910605	-2.47386078

C	4.98756654	-2.52170290	-2.62774351
C	4.55793840	-3.31670433	-1.55094468
C	4.35257423	-2.76297714	-0.32685332
H	5.64275208	0.70247995	-3.44303408
C	5.54178973	-0.35682744	-3.55516990
C	5.27812030	-3.10743376	-3.86549947
H	4.39868379	-4.36506860	-1.69401930
C	5.68776518	-2.33380478	-4.90291544
C	5.82105970	-0.94378380	-4.74615370
H	5.17736564	-4.16492239	-3.99382016
H	5.91059779	-2.78566283	-5.84688046
H	6.14461231	-0.34435797	-5.57132186
O	2.72051327	0.89500778	0.23166492
O	4.56565474	-0.83044984	1.18724495
P	3.11906672	-0.05417778	1.56517456
O	1.98444905	-1.08594639	1.82888527
S	3.65711276	0.07530743	4.32612366
O	2.92805417	-1.19678200	4.42605519
O	5.10110833	-0.17840163	4.42727976
C	3.15039185	1.15497660	5.64746704
F	1.82391251	1.38803818	5.55454320
F	3.82011772	2.32353995	5.55566832
F	3.42283451	0.57220186	6.83433207
N	3.31201863	0.81348873	2.82276129
**Bq**	**2.94113477**	**3.75364254**	**1.24573233**
**Bq**	**3.82325063**	**-3.77517258**	**1.02524760**
H	2.00896555	-1.63580194	2.61543331

NTP_CB

C	4.79198602	1.88538068	-0.85935804
C	5.95704748	2.43169448	-1.29680640
C	7.15592486	1.70122679	-1.22460411
C	7.17843083	0.44124273	-0.62035785
C	6.00164625	-0.08415867	-0.08138252
C	4.78358441	0.59068031	-0.32400792
H	8.33244965	3.19735011	-2.21443472
H	5.96047500	3.42069424	-1.70517837
C	8.33900423	2.23289382	-1.75109774
C	8.37937863	-0.27302717	-0.54677580
C	9.51499157	0.25844100	-1.06530903
C	9.49452078	1.52482563	-1.67387367
H	8.40216332	-1.23669485	-0.08231321
H	10.43201509	-0.29013594	-1.01025181
H	10.39630494	1.93401388	-2.07915178
C	5.19989185	-1.58104496	1.87228181
C	6.04764808	-1.39269553	0.75703146
C	6.94677856	-2.40696428	0.41911679
C	6.86557130	-3.64252438	1.06717268
C	5.89180661	-3.86134602	2.05712356
C	5.07745123	-2.84888973	2.45580324
H	7.97868146	-1.26021735	-1.07173379
C	7.91470108	-2.20345228	-0.57062066
C	7.75767237	-4.66288577	0.71708661
H	5.79602725	-4.82859741	2.50450603
C	8.69252617	-4.44708920	-0.24313359
C	8.77195628	-3.20423073	-0.89389750
H	7.70269731	-5.61285206	1.20641442
H	9.37373093	-5.22906330	-0.50652797
H	9.51302949	-3.04612712	-1.64935356
O	3.54200852	-0.08426296	-0.06524292
O	4.50947008	-0.45112615	2.42961455
P	3.11906672	-0.05417778	1.56517456
O	1.98444905	-1.08594639	1.82888527
S	2.08109920	1.61756020	3.57937798
O	1.36674041	0.42664151	4.06013618
O	3.22962849	1.88691216	4.45576492
C	0.98077988	3.01663368	3.59642882
F	-0.07427896	2.76920229	2.79136452
F	1.63700219	4.11063225	3.15479612
F	0.54510454	3.23116153	4.85605765
N	2.63295455	1.34582487	1.98384811
**Bq**	**3.28594852**	**2.80917161**	**-0.96594761**
**Bq**	**3.86272420**	**-3.13195460**	**3.71167446**

IDP_H

C	-3.17854294	7.95331558	-1.94949586
C	-1.88642624	7.61978754	-2.18593766
C	-1.40628911	6.36669095	-1.79136500
C	-2.27049844	5.46234594	-1.16616982
C	-3.59865234	5.82135649	-0.93557283
C	-4.04457022	7.04564678	-1.31594688
H	0.56811569	6.69448921	-2.56112228
H	-3.54123945	8.91480066	-2.24865270
H	-1.23348840	8.31349842	-2.67402766
C	-0.06912299	6.01975624	-2.03073786
C	-1.79892347	4.20095130	-0.79467246
H	-4.26336481	5.13226142	-0.45997432
H	-5.06241408	7.32076179	-1.13280032
C	-0.41366849	3.94809302	-0.89387509
C	0.42136733	4.83592104	-1.58444590
C	-2.78866946	3.11320532	-0.27596171
C	-3.89045638	3.47689427	0.50570894
C	-2.60452084	1.74473004	-0.58937833
C	-4.20885396	4.82499955	0.71420991
C	-4.67367959	2.48115935	1.09887790
C	-3.34577540	0.76079882	0.08100478
C	-5.27803789	5.15854735	1.48035499
H	-3.60886349	5.59081730	0.27037325
C	-5.77246706	2.84806706	1.88636749
C	-4.35732529	1.12286196	0.91201998
C	-6.06953872	4.15929408	2.07183926
H	-5.52068019	6.18945978	1.63561929
H	-6.37909348	2.09483080	2.34261068
H	-4.92239703	0.37138505	1.42152797
H	-6.91264012	4.43281157	2.67214155
O	-1.69156509	1.38282233	-1.63660430
O	0.13816418	2.80021397	-0.23884333
P	-0.08051363	1.39359634	-1.13668075
P	1.52201532	-0.81135435	-0.52187867

O	2.60177182	-0.82339202	0.77564207
O	1.01821389	-2.40328222	-0.73087447
C	2.14799759	-1.62706065	1.88193523
C	2.12655088	-3.31712472	-0.72621603
C	2.32098782	-3.03116440	1.83719744
C	1.58990157	-1.02599590	3.01526710
C	2.67139001	-3.75161899	0.50447285
C	2.63701321	-3.81145498	-1.93119052
C	2.18271035	-3.76634285	3.02137171
C	1.36849512	-1.76361130	4.13615588
C	3.53314564	-4.85296955	0.50786516
C	3.54305659	-4.82415835	-1.92378426
C	2.49948744	-5.13073741	3.06417512
C	1.70595726	-3.12879531	4.17169018
H	0.94077777	-1.29896460	4.99982352
C	3.97348874	-5.38531889	-0.70751214
C	3.97397655	-5.41547126	1.71228329
H	3.92755953	-5.19979394	-2.84953120
C	2.35081105	-5.82572142	4.21981125
H	2.85945844	-5.62587640	2.18590147
C	1.56145887	-3.86485302	5.35524127
C	4.85348977	-6.47542764	-0.70434512
C	4.82649707	-6.47208739	1.69393072
H	3.64028863	-5.01221540	2.64508559
C	1.87858647	-5.18438503	5.37807142
H	2.59344859	-6.86822948	4.24948892
H	1.20130658	-3.38735105	6.24273697
C	5.27153520	-7.00725103	0.47327987
H	5.19746589	-6.88926706	-1.62950788
H	5.16228842	-6.89903359	2.61560317
H	1.76818520	-5.74064318	6.28493128
H	5.94538942	-7.83960016	0.47136302
N	0.26475667	0.12788300	-0.23226063
O	0.81516711	1.38520907	-2.34660952
O	2.22040513	-0.30268788	-1.75437799
**Bq**	**2.12078803**	**4.42446805**	**-1.85795242**
**Bq**	**-2.97704031**	**-0.95103567**	**-0.17428909**
**Bq**	**2.08478709**	**-3.12617068**	**-3.46672729**
**Bq**	**1.19528054**	**0.69952595**	**2.99261676**
H	-0.32761974	-0.08551477	0.54462519

IDP_CB

C	-3.17854294	7.95331558	-1.94949586
C	-1.88642624	7.61978754	-2.18593766
C	-1.40628911	6.36669095	-1.79136500
C	-2.27049844	5.46234594	-1.16616982
C	-3.59865234	5.82135649	-0.93557283
C	-4.04457022	7.04564678	-1.31594688
H	0.56811569	6.69448921	-2.56112228
H	-3.54123945	8.91480066	-2.24865270
H	-1.23348840	8.31349842	-2.67402766
C	-0.06912299	6.01975624	-2.03073786
C	-1.79892347	4.20095130	-0.79467246
H	-4.26336481	5.13226142	-0.45997432
H	-5.06241408	7.32076179	-1.13280032
C	-0.41366849	3.94809302	-0.89387509
C	0.42136733	4.83592104	-1.58444590
C	-2.78866946	3.11320532	-0.27596171
C	-3.89045638	3.47689427	0.50570894
C	-2.60452084	1.74473004	-0.58937833
C	-4.20885396	4.82499955	0.71420991
C	-4.67367959	2.48115935	1.09887790
C	-3.34577540	0.76079882	0.08100478
C	-5.27803789	5.15854735	1.48035499
H	-3.60886349	5.59081730	0.27037325
C	-5.77246706	2.84806706	1.88636749
C	-4.35732529	1.12286196	0.91201998
C	-6.06953872	4.15929408	2.07183926
H	-5.52068019	6.18945978	1.63561929
H	-6.37909348	2.09483080	2.34261068
H	-4.92239703	0.37138505	1.42152797
H	-6.91264012	4.43281157	2.67214155
O	-1.69156509	1.38282233	-1.63660430
O	0.13816418	2.80021397	-0.23884333
P	-0.08051363	1.39359634	-1.13668075
P	1.52201532	-0.81135435	-0.52187867
O	2.60177182	-0.82339202	0.77564207
O	1.01821389	-2.40328222	-0.73087447
C	2.14799759	-1.62706065	1.88193523
C	2.12655088	-3.31712472	-0.72621603
C	2.32098782	-3.03116440	1.83719744
C	1.58990157	-1.02599590	3.01526710
C	2.67139001	-3.75161899	0.50447285
C	2.63701321	-3.81145498	-1.93119052
C	2.18271035	-3.76634285	3.02137171
C	1.36849512	-1.76361130	4.13615588
C	3.53314564	-4.85296955	0.50786516
C	3.54305659	-4.82415835	-1.92378426
C	2.49948744	-5.13073741	3.06417512
C	1.70595726	-3.12879531	4.17169018
H	0.94077777	-1.29896460	4.99982352
C	3.97348874	-5.38531889	-0.70751214
C	3.97397655	-5.41547126	1.71228329
H	3.92755953	-5.19979394	-2.84953120
C	2.35081105	-5.82572142	4.21981125
H	2.85945844	-5.62587640	2.18590147
C	1.56145887	-3.86485302	5.35524127
C	4.85348977	-6.47542764	-0.70434512
C	4.82649707	-6.47208739	1.69393072
H	3.64028863	-5.01221540	2.64508559
C	1.87858647	-5.18438503	5.37807142
H	2.59344859	-6.86822948	4.24948892
H	1.20130658	-3.38735105	6.24273697
C	5.27153520	-7.00725103	0.47327987
H	5.19746589	-6.88926706	-1.62950788
H	5.16228842	-6.89903359	2.61560317
H	1.76818520	-5.74064318	6.28493128
H	5.94538942	-7.83960016	0.47136302
N	0.26475667	0.12788300	-0.23226063
O	0.81516711	1.38520907	-2.34660952
O	2.22040513	-0.30268788	-1.75437799
**Bq**	**2.12078803**	**4.42446805**	**-1.85795242**
**Bq**	**-2.97704031**	**-0.95103567**	**-0.17428909**
**Bq**	**2.08478709**	**-3.12617068**	**-3.46672729**
**Bq**	**1.19528054**	**0.69952595**	**2.9926167**

#4 – Extracting and export ensemble-level descriptors

The calculated representations for each BAOC are shown in *dataset_active-site.csv*. The buried volume representation is reported across the indicated distances at either '1O' or '2H', corresponding to either the heteroatom ('O' for CPA, H8CPA, and SPA; 'N' for IDP, NTP) and the acidic proton ('H') respectively.

Unique to the BAOC dataset is *calc. pK_a* for each ensemble generated. The *calc. pK_a* of each BAOC is computed from an xTB-based benchmark of the corresponding ensemble-level *deprotonation energy* (ΔG_{deprot}, in Hartrees) between calculated acid ('H') and conjugate base ('CB') of a selected array of experimentally determined acidities in MeCN. The xTB-based benchmark was validated with a higher-level DFT-based benchmark and an experimental ground truth (observed acidity in acetonitrile from Kutt, A. et al. *Eur. J. Org. Chem.* **2021**[60]). Each BAOC computed was organized into one of two bins relating to the general characteristics of the acidic proton: (1) phosphate-like, or (2) sulfonamide-like. In this work, phosphate-like acids include CPA, H8CPA, SPA, and IDP. The sulfonamide-like acids include NTP.

Phosphate-like calc. pK_a conversion

Ground state ('C_0') calc. pK$_a$ $= -0.142830176 * \Delta G_{deprot} - 9.073037818$

Boltzmann avg. ('AV_XTB') calc. pK$_a$ $= -0.143257524 * \Delta G_{deprot} - 9.136612018$

Minimum ('MIN') calc. pK$_a$ $= -0.142830176 * \Delta G_{deprot} - 9.073037818$

Maximum ('MAX') calc. pK$_a$ $= -0.096629251 * \Delta G_{deprot} - 1.88123327$

Sulfonamide-like calc. pK_a conversion

Ground state ('C_0') calc. pK$_a$ $= -0.24500443 * \Delta G_{deprot} - 26.55425678$

Boltzmann avg. ('AV_XTB') calc. pK$_a$ $= -0.245285358 * \Delta G_{deprot} - 26.60609466$

Minimum ('MIN') calc. pK$_a$ $= -0.24500443 * \Delta G_{deprot} - 26.55425678$

Maximum ('MAX') calc. pK$_a$ $= -0.24275215 * \Delta G_{deprot} - 26.39632654$

Altogether, the ensembles computed for each BAOC are tagged at the end of each descriptor, including: (1) xTB-based ground state (written as 'C_0'), (2) xTB-based Boltzmann average (written as 'AV_XTB'), (3) minimum value across all conformers (written as 'MIN'), and (4) maximum value across all conformers (written as 'MAX'). It should be noted that DFT-based ensembles (like 'GD' and 'AV_DFT') are not computed for the BAOCs because of the excessive computational cost of even single-point energies on the large molecules. xTB-based descriptors are solely extracted and deployed in the reported correlations herein.

Visualizations of Buried Volume with SambVca 2.1

Throughout the main text, *active site-based* buried volume was visualized with SambVca 2.1[56] to contextualize changes in confinement at the active site as it reflects the observed stereoselectivity. As the buried volumes are computed from ensembles of chiral acids, we instead use the ground state (lowest energy from xTB single point after conformational searching, *vida supra*) conformation identified for visualization. The coordinates for these ground state conformers are printed below corresponding to their label in the main text:

TRIP-**CPA**

O	-0.7540616335	1.9108734094	-0.9706461343
H	-0.7162905563	2.8492358236	-0.7914107526
C	-2.6258202481	-3.0724675463	-1.2528755066
C	-1.2378920936	-3.0625392624	-0.9563457865
C	-0.7057181199	-1.9855836146	-0.1893266030
C	-1.5227464830	-0.9249937587	0.1282095721
H	-4.2271304059	-4.1584612946	-2.1834192058
H	-4.4842596240	-2.0046402975	-1.0813509619
C	-3.1662175347	-4.1548349387	-1.9786021032
C	-0.4332980277	-4.1024382363	-1.4674380753
C	-0.9849271273	-5.1281202496	-2.1842763354
C	-2.3654880657	-5.1666219349	-2.4276751097
H	0.6318047117	-4.0761156823	-1.2965194425
H	-0.3553683528	-5.9153224071	-2.5717668469
H	-2.7855937496	-5.9907740115	-2.9849590284
C	1.5013857968	-0.8945257620	-0.0618748726
C	0.7061403485	-1.9771021223	0.2409501314
C	1.2663601934	-3.0643663487	0.9716709947
C	2.6575743465	-3.0564286867	1.2525872640
C	3.4405433477	-1.9566097697	0.8498258048
C	2.8811854660	-0.8634960839	0.2387384659
H	-0.5824837963	-4.1169912227	1.3071296354
C	0.4845068783	-4.1282754336	1.4685708746
C	3.2226931472	-4.1421025968	1.9542174148
H	4.5006202013	-1.9659953557	1.0560077785
C	2.4433587412	-5.1749893497	2.3927805154
C	1.0602501008	-5.1570909495	2.1613526989
H	4.2853536914	-4.1313211775	2.1490180409
H	2.8825218416	-6.0012115913	2.9320067554
H	0.4481954314	-5.9625016753	2.5394084641
O	-1.0177795420	0.1409251039	0.8562449147
O	0.9497441820	0.1979129071	-0.7185718475
P	0.0226788077	1.1404074409	0.1840277506
O	0.6532972526	2.0113178216	1.1649442801
C	-2.8945568863	-0.8982811208	-0.2112667398
C	-3.4292227010	-1.9871016216	-0.8508262991
C	3.6737141785	0.3366051177	-0.1107487811
C	3.9262825680	0.6369561002	-1.4520335734
C	4.6476946453	1.7809761691	-1.7602493599
C	5.1259417698	2.6284096722	-0.7749901504
C	4.8792883633	2.3013994601	0.5488434296
C	4.1588152523	1.1711524828	0.9023686524
C	7.3218334851	3.8143808545	-0.5730282204
C	5.9023288697	3.8700126131	-1.1414445525
C	5.1729245977	5.1255598618	-0.6594616600
C	3.4839319516	-0.2824853008	-2.5684644372
C	2.5625618924	0.4415457220	-3.5514649704
C	4.7046949777	-0.8629525262	-3.2882953319
C	3.9374362949	0.8680360386	2.3668841196
C	3.2797579958	2.0433646796	3.0914824315
C	5.2677394949	0.4979963160	3.0307238593
H	7.8318755977	2.9167399086	-0.9161336908
H	7.8924302804	4.6826995569	-0.8961798571
H	7.3025522051	3.8027689494	0.5137543317
H	5.9758694317	3.9169940288	-2.2341619833
H	4.1638194157	5.1543078608	-1.0644572508
H	5.1044202446	5.1395866762	0.4251959943
H	5.7037114482	6.0189870099	-0.9824271730
H	4.8474423222	2.0192512479	-2.7951482640
H	2.9241732410	-1.1187910935	-2.1394364720
H	1.6992654303	0.8407118635	-3.0248244910
H	2.2139253997	-0.2504265850	-4.3158031143
H	3.0800249549	1.2619766113	-4.0425972047
H	4.3889855720	-1.5693278999	-4.0534003102
H	5.2855645636	-0.0775733739	-3.7653343986
H	5.3478317790	-1.3831460062	-2.5813592879
H	3.2614518170	0.0125301062	2.4531756482
H	3.0570949173	1.7643911214	4.1194300887
H	3.9297612928	2.9144153582	3.1096635859
H	2.3516133347	2.3075399733	2.5934956040
H	5.7323920879	-0.3435568818	2.5210985160
H	5.1050598930	0.2247395063	4.0711589647
H	5.9597457492	1.3363482043	2.9997897139
H	5.2558662790	2.9445951096	1.3285878062
C	-3.6916799425	0.3084609663	0.1031947684
C	-3.9578385184	0.6477003497	1.4328544405
C	-4.6585981455	1.8143191626	1.7019021151
C	-5.1007928051	2.6497815023	0.6900853099
C	-4.8479857592	2.2818105586	-0.6220664563
C	-4.1516086599	1.1252195503	-0.9375604392
C	-7.2542830580	3.9011579631	0.4182810672
C	-5.8456175348	3.9216958431	1.0150666283
C	-5.0650906158	5.1432674836	0.5250909445
C	-3.5620228183	-0.2578168227	2.5767958622
C	-2.6929511339	0.4753614269	3.5997566208
C	-4.8169829648	-0.8346967939	3.2394055676
C	-3.9413855062	0.7714132468	-2.3938983693
C	-3.2524507766	1.8977350686	-3.1664200229
C	-5.2862147014	0.4233198173	-3.0399461962
H	-7.8039506780	4.7908653028	0.7182829012
H	-7.2149487487	3.8740131912	-0.6677864003
H	-7.7983377179	3.0245970059	0.7633813311
H	-5.9393938792	3.9930853566	2.1045489723
H	-4.9689100302	5.1314369792	-0.5578858719

H	-5.5765244245	6.0590723592	0.8140286324	H	-5.4185918035	-1.3719601688	2.5088289461
H	-4.0670631772	5.1517226726	0.9579570628	H	-3.2949892267	-0.1080945610	-2.4574228383
H	-4.8694424559	2.0809452980	2.7271422741	H	-3.0907117502	1.5905899001	-4.1977828599
H	-2.9808223312	-1.0960635350	2.1821678414	H	-3.8531037888	2.8037051503	-3.1734612237
H	-2.3630525222	-0.2179046771	4.3708754231	H	-2.2886917966	2.1199089586	-2.7174810870
H	-3.2440778419	1.2805666757	4.0792011640	H	-5.9473018740	1.2867110210	-3.0438819121
H	-1.8157014356	0.8955019078	3.1138517360	H	-5.7792287882	-0.3773678832	-2.4930984967
H	-4.5384581742	-1.5248916575	4.0329257208	H	-5.1375107723	0.0992808700	-4.0678465872
H	-5.4267562486	-0.0442304497	3.6700284813	H	-5.2046585083	2.9116461398	-1.4223582060

TRIP-$\mathbf{H_8CPA}$

O	-0.7816535231	1.9713876825	-0.9542249497	C	4.9745956167	2.3193620304	0.5207273654
H	-0.7416644414	2.9082309604	-0.7676000783	C	4.2076606766	1.2261898825	0.8926030087
C	-2.5239251641	-2.9317259397	-1.3896780840	C	7.4836828150	3.7170209284	-0.6046609816
C	-1.1727567527	-2.9146714372	-1.0528644209	C	6.0751054676	3.8059191833	-1.1954876428
C	-0.6934565569	-1.8977035916	-0.2153929690	C	5.3836835277	5.0989607674	-0.7586925831
C	-1.5294256511	-0.8446685672	0.1340181276	C	3.5250771407	-0.2894396469	-2.5507768149
H	-3.9035100561	-3.6575364757	-2.8483596591	C	2.6137067931	0.4394902767	-3.5396358292
H	-4.4043095063	-1.9345625400	-1.2096687871	C	4.7308242269	-0.9037416575	-3.2679449819
C	-3.1283882400	-4.0555759540	-2.1899570156	C	3.9540820882	0.9726021560	2.3613076149
C	-0.2167112066	-3.9017858471	-1.6700395803	C	3.3112157212	2.1846423076	3.0369063014
C	-0.9124524417	-5.1842325661	-2.1100118064	C	5.2627221097	0.5919328269	3.0613432950
C	-2.0969230136	-4.8320655946	-2.9997814989	H	7.4486301495	3.7352965784	0.4816474858
H	0.2211650070	-3.4166188850	-2.5502161214	H	7.9669980742	2.7934286920	-0.9161413351
H	-0.2061416539	-5.8198525344	-2.6473805728	H	8.0886889290	4.5562527466	-0.9419577148
H	-2.5540157030	-5.7352038629	-3.4087951789	H	6.1655601417	3.8202079990	-2.2878965031
C	1.5125303663	-0.8069888235	-0.0642796899	H	5.9500623651	5.9643112874	-1.0974244002
C	0.7043287464	-1.8866597402	0.2733571348	H	4.3820732880	5.1515380508	-1.1795317736
C	1.2199780819	-2.9170909509	1.0711859541	H	5.3003270034	5.1448842025	0.3240632548
C	2.5769516498	-2.9140278746	1.3842091178	H	4.9732735991	1.9526603607	-2.8145738319
C	3.3823553941	-1.8714101021	0.9443353391	H	2.9515933359	-1.1082130321	-2.1066238105
C	2.8647989893	-0.7785238847	0.2636969637	H	2.2594641813	-0.2514525498	-4.3025590957
H	-0.5336326665	-4.1663796753	1.0039051243	H	3.1416753157	1.2522410801	-4.0324502035
C	0.2944834102	-3.9412576593	1.6746832395	H	1.7541167572	0.8503455381	-3.0162023621
C	3.2166539654	-4.0393576121	2.1544970666	H	4.3979107165	-1.6214423434	-4.0152126876
H	4.4381077387	-1.8866441987	1.1722740828	H	5.3200618710	-0.1388557032	-3.7673039058
C	2.2128326316	-4.8595589823	2.9560253020	H	5.3715923434	-1.4186932219	-2.5551041660
C	1.0261606357	-5.2177633735	2.0715288797	H	3.2554634984	0.1371128309	2.4591838786
H	3.7215562687	-4.6938872615	1.4352200963	H	3.9733107280	3.0466392036	3.0231737015
H	2.6955823157	-5.7614155045	3.3374796802	H	2.3884629512	2.4408964417	2.5250901576
H	0.3424429959	-5.8844709503	2.6004071899	H	3.0805599883	1.9495471631	4.0740855454
O	-1.0119638834	0.2058542261	0.8817952699	H	5.7189126027	-0.2727407640	2.5834748150
O	0.9411418205	0.2708439942	-0.7360890752	H	5.0740420374	0.3506795625	4.1054200508
P	0.0192468983	1.2016803067	0.1866896314	H	5.9737990986	1.4137994460	3.0226893083
O	0.6641855634	2.0769997000	1.1555863542	H	5.3648175775	2.9685033508	1.2889531486
C	-2.8709689993	-0.8219550430	-0.2389386185	C	-3.7199645341	0.3428420019	0.0952535187
C	-3.3551913941	-1.9080599719	-0.9537005072	C	-4.0218946666	0.6312944732	1.4296707418
H	3.9862397064	-3.6348421199	2.8152746334	C	-4.7755594445	1.7591032991	1.7208941072
H	1.8615421193	-4.2784266050	3.8110730332	C	-5.2403929153	2.6037134375	0.7272102875
H	1.3794957835	-5.7372641401	1.1776437441	C	-4.9528901940	2.2851937957	-0.5906041705
H	-0.1424430200	-3.4899009446	2.5730071814	C	-4.2006091769	1.1704827831	-0.9275489055
H	0.6079752826	-4.1257813811	-0.9951470001	C	-7.4516431387	3.7504611048	0.4707992161
H	-1.2635232944	-5.7357774768	-1.2346890690	C	-6.0489232200	3.8294243447	1.0768914057
H	-1.7498683383	-4.2220763639	-3.8363698107	C	-5.3303310210	5.1008456569	0.6204883181
H	-3.6211807519	-4.7386781259	-1.4890848600	C	-3.6077459865	-0.2878288172	2.5563549870
C	3.7039294671	0.3831452345	-0.1043335626	C	-2.7573536538	0.4467806686	3.5939644109
C	3.9866227447	0.6387366716	-1.4492663345	C	-4.8491920692	-0.9072170705	3.2061263496
C	4.7528563720	1.7479207788	-1.7765823059	C	-3.9539525047	0.8706401259	-2.3903479844
C	5.2499125528	2.6028476071	-0.8074261515	C	-3.2996808466	2.0467027054	-3.1173747007

C	-5.2734453402	0.4894476152	-3.0694445035
H	-8.0467235260	4.6060735166	0.7832440647
H	-7.4043119754	3.7452028910	-0.6153087154
H	-7.9533108029	2.8415639217	0.7960019845
H	-6.1518879154	3.8702905773	2.1671792673
H	-5.2327244466	5.1222808187	-0.4621779019
H	-5.8873577833	5.9820921564	0.9319745292
H	-4.3343137631	5.1480164421	1.0554007435
H	-5.0116317630	1.9869774157	2.7501369267
H	-3.0065472312	-1.1038135824	2.1458046825
H	-1.8815859959	0.8816178565	3.1187405599
H	-2.4256825309	-0.2485317704	4.3626980360
H	-3.3228722536	1.2412267139	4.0745252095
H	-5.4408283439	-1.4373213012	2.4623410621
H	-4.5551493375	-1.6123276305	3.9808783238
H	-5.4747665301	-0.1419940877	3.6588982253
H	-3.2705019042	0.0210822975	-2.4677188056
H	-3.9271258670	2.9338708025	-3.0897051847
H	-2.3434539082	2.2793044101	-2.6581330777
H	-3.1280497642	1.7854380161	-4.1598032224
H	-5.9659843683	1.3277949268	-3.0641863785
H	-5.7462765187	-0.3420699570	-2.5513784851
H	-5.0957162087	0.1975648935	-4.1025112529
H	-5.3264479419	2.9214976536	-1.3781075177

TRIP-**SPA**

O	0.0106838280	-2.7176960361	0.3754401899
H	-0.0184622542	-2.9524349232	-0.5520728821
C	2.3761614115	3.3022424442	0.2910511844
C	1.0930958299	2.9259841646	-0.0477522122
C	0.7004833168	1.5905825158	-0.0738576584
C	1.6761589118	0.6125447854	-0.1675572201
H	4.3932552436	2.5499460454	0.4261088842
C	-1.7017948634	0.6022088928	0.0514893821
C	-0.7193551053	1.5645138419	0.2107822338
C	-1.1031306566	2.8723237493	0.4895597180
C	-2.3797743755	3.3270072328	0.2293051037
C	-3.3671239067	2.3729231054	0.0503953902
C	-3.0377795577	1.0045999434	0.0343376246
H	-4.3955687189	2.6496807183	-0.1297218813
O	1.2989056859	-0.7190683220	-0.2550587515
O	-1.3156553866	-0.7099941764	-0.1786927193
P	0.0175363683	-1.1667492522	0.6333612307
O	0.0561322503	-0.7343909127	2.0199893344
C	3.0166751034	0.9983422099	-0.0879680933
C	3.3584197321	2.3298568210	0.2091564381
C	2.2468986311	4.6545050625	0.9487724842
H	2.3718131327	5.4635196852	0.2250756566
H	2.9624659456	4.7989279869	1.7566693206
C	0.0001028250	3.8799539153	0.3417680222
C	-2.2368335460	4.7976541383	-0.0789852709
H	-2.9422267247	5.1412161983	-0.8342392415
H	-2.3660950034	5.4056705409	0.8191770192
C	0.7776266087	4.6107676834	1.4676610661
H	0.3826179761	5.6036389861	1.6776142097
H	0.7440921967	4.0185288757	2.3829863986
C	-0.7616365412	4.8726206223	-0.5761702758
H	-0.3601219960	5.8836303516	-0.5277851686
H	-0.7194158288	4.5295666976	-1.6111815769
C	-4.0932985504	-0.0232447555	-0.1450903684
C	-4.5278687104	-0.3677033734	-1.4259587167
C	-5.4913105690	-1.3565238778	-1.5635198609
C	-6.0336250256	-2.0023875576	-0.4641192144
C	-5.5855576028	-1.6450539446	0.7981910967
C	-4.6208389235	-0.6656755659	0.9765753044
C	-8.3966184672	-2.6611299151	0.0254996160
C	-7.0841432913	-3.0713701658	-0.6455595181
C	-6.5945721621	-4.4156714982	-0.1033413332
C	-3.9256427565	0.2831329548	-2.6507423416
C	-2.9808342045	-0.7064870333	-3.3377867035
C	-4.9941112844	0.7854323968	-3.6228818153
C	-4.1439797266	-0.3175448623	2.3675623822
C	-3.3857501543	-1.4976969342	2.9787146418
C	-5.3114971186	0.1074954075	3.2613290397
H	-8.7390925443	-1.7057461178	-0.3662776174
H	-9.1656817633	-3.4081448329	-0.1600471139
H	-8.2663097918	-2.5628485275	1.1002208736
H	-7.2730216615	-3.1860717694	-1.7192045162
H	-6.4298974123	-4.3629377317	0.9698111114
H	-7.3307175920	-5.1926564804	-0.2992977919
H	-5.6576163563	-4.6968670812	-0.5792148416
H	-5.8268978609	-1.6382829143	-2.5510416990
H	-3.3335635603	1.1459107448	-2.3292076092
H	-2.5054983713	-0.2431827492	-4.2002803955
H	-3.5254067238	-1.5868116333	-3.6722787618
H	-2.2082976067	-1.0237593877	-2.6410146861
H	-4.5288874083	1.3543121524	-4.4254110192
H	-5.5431295539	-0.0393779915	-4.0694044609
H	-5.7017706869	1.4307948509	-3.1070896677
H	-3.4495324595	0.5255601102	2.2984408154
H	-2.5118430537	-1.7376428154	2.3767516471
H	-3.0450303820	-1.2447958580	3.9799535596
H	-4.0197707393	-2.3795374737	3.0392164191
H	-5.9949603254	-0.7194486709	3.4367725265
H	-5.8670091613	0.9203882761	2.7979171481
H	-4.9376713965	0.4489042880	4.2240630536
H	-5.9916552602	-2.1443842786	1.6642506916
C	4.0762529017	-0.0358057754	-0.1941377372
C	4.4979364349	-0.7334457772	0.9385943413
C	5.4922727659	-1.6908603380	0.8033329005
C	6.0600714778	-1.9830117045	-0.4266826885
C	5.6125685449	-1.2910055217	-1.5411713821
C	4.6329567511	-0.3144220570	-1.4442146266
C	6.6125602633	-4.4068554767	-0.1353994945
C	7.1363322164	-3.0333678083	-0.5598081266
C	8.3741128232	-2.6495755440	0.2536095312
C	3.8927109372	-0.4651424099	2.2966115385
C	3.2655809895	-1.7352689000	2.8733417832
C	4.9452648761	0.1086838771	3.2484523775
C	4.1296599111	0.3993116867	-2.6787158413
C	3.1405898645	-0.5106951191	-3.4127294609
C	5.2595055418	0.8385517555	-3.6104206747
H	5.7307167757	-4.6707887482	-0.7151341823
H	7.3739678555	-5.1675912801	-0.2952959072
H	6.3417562097	-4.4088797279	0.9173039221
H	7.4281192224	-3.0916157603	-1.6147905838

H	9.1668678803	-3.3793793521	0.1026465217
H	8.7397657317	-1.6718967748	-0.0529404823
H	8.1417779964	-2.6107148041	1.3148444837
H	5.8264424385	-2.2248952455	1.6793937022
H	3.0936327472	0.2740169570	2.1875920012
H	2.5124301610	-2.1231499322	2.1906693002
H	2.7752836895	-1.5100879488	3.8170616257
H	4.0139363292	-2.5053896905	3.0453915036
H	4.4920528306	0.3452442885	4.2084248337
H	5.7493628503	-0.6042974247	3.4148793564
H	5.3752925484	1.0187280813	2.8348268338
H	3.5908417333	1.2978812163	-2.3596358947
H	2.7316027347	-0.0044694405	-4.2849709920
H	3.6352995931	-1.4230046667	-3.7395896862
H	2.3220103236	-0.7824846405	-2.7502233116
H	6.0115570683	1.3970081510	-3.0575672493
H	4.8631985795	1.4778716758	-4.3966975262
H	5.7394435111	-0.0145030504	-4.0828937787
H	6.0402564617	-1.5233889698	-2.5052813763

CPA-**2**

O	-0.6907074372	1.9178833086	-0.8025394478
H	-0.6110244469	2.8366427485	-0.5516338421
C	-2.6357992625	-3.2532832990	-1.2748601653
C	-1.2496586265	-3.1993946455	-0.9739604934
C	-0.7521760394	-2.1080159354	-0.1976381241
C	-1.5926356935	-1.0532039742	0.0683268038
H	-4.2066309179	-4.4057597374	-2.1765421154
H	-4.5139708884	-2.2074022392	-1.1784264784
C	-3.1458135603	-4.3666807215	-1.9739725836
C	-0.4147077538	-4.2160338996	-1.4815316120
C	-0.9360924750	-5.2694583690	-2.1813943126
C	-2.3161072636	-5.3615452561	-2.4088496775
H	0.6510388173	-4.1506058750	-1.3268458801
H	-0.2815190177	-6.0365873615	-2.5674047913
H	-2.7132500518	-6.2088309645	-2.9478648196
C	1.4508954067	-1.0140921535	0.0758311065
C	0.6379182726	-2.1056036816	0.2914568501
C	1.1798545055	-3.2451889698	0.9578549025
C	2.5759046580	-3.2993814097	1.2030454356
C	3.3791739453	-2.1901549231	0.8695176700
C	2.8317206391	-1.0351433469	0.3754432560
H	-0.6959706863	-4.2487157123	1.2961112110
C	0.3748632288	-4.3083098579	1.4164688851
C	3.1229006763	-4.4475759388	1.8122662858
H	4.4437873441	-2.2393596461	1.0497146433
C	2.3204650591	-5.4803805718	2.2081487072
C	0.9326847642	-5.3973940055	2.0275730626
H	4.1903685808	-4.4846009027	1.9763860393
H	2.7457882450	-6.3543605874	2.6787701054
H	0.3021997214	-6.2001680878	2.3798363468
O	-1.1601877154	0.0089123514	0.8475322815
O	0.9397499218	0.1242924658	-0.5358098047
P	-0.0471822033	1.0275150915	0.3458866804
O	0.5039880593	1.8130182446	1.4406438393
C	-2.9468774686	-1.0419808324	-0.3409115837
C	-3.4635789499	-2.1649464853	-0.9289843845
C	3.6480380145	0.1726180468	0.1200221108
C	4.0392251916	0.4875243922	-1.1949029440
C	4.7190721245	1.6804136566	-1.4058091157
C	5.1191883855	2.5143427604	-0.3766248080
C	4.7883850988	2.1309508943	0.9072303671
C	4.0161740904	1.0063556355	1.1905444892
C	7.2022121095	3.4094179405	-1.4085376589
C	5.8957028331	3.7835514532	-0.6947004185
C	5.0397733663	4.6745971945	-1.6056721360
C	6.2514409127	4.5904210538	0.5570885739
C	3.8073187387	-0.3354294108	-2.4729018162
C	5.1492751736	-0.4716015727	-3.2213365858
C	2.7929666222	0.4222142154	-3.3437560323
C	3.2901364214	-1.7694307949	-2.3148691542
C	3.7168256181	0.8216708118	2.6877288838
C	3.1763673676	2.1435154270	3.2656422483
C	5.0405080588	0.4482453451	3.3769120372
C	2.6765479184	-0.2302176314	3.0872924964
H	7.7816992786	4.3043349215	-1.6266332504
H	7.0092438049	2.8930666159	-2.3447152040
H	7.8003872181	2.7555455867	-0.7773127631
H	5.5655881984	5.6012116718	-1.8275417536
H	4.8129126532	4.1802287526	-2.5461863127
H	4.1003085245	4.9178674552	-1.1139092391
H	5.3552954211	4.8933187327	1.0935258127
H	6.8924827838	4.0180330583	1.2237497343
H	6.7902119023	5.4907154644	0.2672843915
H	4.9617270631	1.9694247541	-2.4139167078
H	5.0285031042	-1.1282797444	-4.0804768307
H	5.5264387025	0.4760076446	-3.5913273120
H	5.9004065796	-0.9034760446	-2.5631664135
H	3.1452304957	1.4199757016	-3.5917623190
H	1.8498993821	0.5184123112	-2.8107994969
H	2.6153053320	-0.1192293722	-4.2711224685
H	3.9147504980	-2.3492207290	-1.6413379708
H	3.3110573888	-2.2532866210	-3.2912152141
H	2.2642236334	-1.7962049561	-1.9673850512
H	2.9592398076	2.0160365030	4.3241775112
H	2.2535560377	2.4024744458	2.7547157929
H	3.8712978343	2.9717387822	3.1765229445
H	5.8003436398	1.2102111673	3.2247536968
H	5.4197506288	-0.4920376019	2.9809456245
H	4.8830261587	0.3309395387	4.4470682748
H	3.0329049496	-1.2449258639	2.9501923122
H	1.7504757876	-0.0900389169	2.5382406599
H	2.4518015077	-0.1051994690	4.1456681864
H	5.1312506907	2.7362037683	1.7252714982
C	-3.7139070792	0.2041402036	-0.1242085755
C	-4.2142773903	0.5196502773	1.1465227848
C	-4.7840140092	1.7765263293	1.3396443842
C	-4.9225313555	2.6952918965	0.3210419461
C	-4.4770988504	2.3219083100	-0.9376641518
C	-3.8472349681	1.1127276626	-1.1963204082
C	-4.4998166364	5.1385422919	0.1368834066
C	-5.5375408312	4.0729730675	0.5176290975
C	-6.7761524504	4.2059897794	-0.3793738958
C	-5.9698704550	4.3279069079	1.9642336214
C	-4.2217751986	-0.3690080335	2.3982918917

C	-5.6722539528	-0.4745911772	2.9082914025	H	-6.3038920998	-0.9186917683	2.1411575952
C	-3.3316999013	0.2968774402	3.4603523878	H	-5.7078471846	-1.1095424582	3.7911481200
C	-3.7345702645	-1.8137199844	2.2495812035	H	-3.7017647716	1.2783077848	3.7427874338
C	-3.4516372453	0.8551508374	-2.6625389439	H	-2.3213163967	0.4138457233	3.0744473052
C	-2.2032487037	-0.0106172365	-2.8922246977	H	-3.2925828491	-0.3207174645	4.3555267216
C	-3.1662441164	2.1800879849	-3.3978715012	H	-4.2838083410	-2.3477420054	1.4797111215
C	-4.6565120243	0.1855721766	-3.3430609875	H	-3.8992594902	-2.3299946690	3.1951232237
H	-3.6123888265	5.0345279354	0.7580011000	H	-2.6727784492	-1.8630447423	2.0360988927
H	-4.9116398024	6.1346369447	0.2863125374	H	-1.3855821658	0.3118902715	-2.2568845759
H	-4.2002243845	5.0471000846	-0.9035530067	H	-1.8803919504	0.0997930329	-3.9263376867
H	-7.5018637325	3.4335404878	-0.1332995171	H	-2.3877151248	-1.0653472743	-2.7320360049
H	-7.2430747388	5.1779377212	-0.2334972867	H	-2.7316277913	1.9674071302	-4.3721345490
H	-6.5188544581	4.1083727224	-1.4303763694	H	-2.4565800168	2.7810423907	-2.8346052726
H	-6.7317441257	3.6172037985	2.2765133538	H	-4.0677186257	2.7587845320	-3.5809533660
H	-5.1219418828	4.2664293280	2.6424318879	H	-4.4609429934	0.0569193817	-4.4057897412
H	-6.3920519945	5.3277554999	2.0456014830	H	-5.5495714906	0.7963274460	-3.2253138248
H	-5.1257831371	2.0398739925	2.3237332933	H	-4.8514741136	-0.7914218045	-2.9104451941
H	-6.0947439596	0.4876814498	3.1783691605	H	-4.6232450313	3.0205562507	-1.7399320425

CPA-**3**

O	-0.8087594802	1.6907641545	-1.0662589676	C	5.1310023052	2.6206688398	-0.4307091242
H	-0.6593329571	2.6345016381	-1.0497546539	C	5.0815454545	2.0635823097	0.8412949093
C	-2.5402401689	-3.0849346732	-1.4779033747	C	4.3541117606	0.9156502144	1.0939158563
C	-1.1847449100	-3.1448348947	-1.0596289963	C	6.9667851347	3.5737996731	-1.8168691100
C	-0.6759407385	-2.1028942557	-0.2355845789	C	5.9072118249	3.8881755943	-0.7511406170
C	-1.4845539044	-1.0338220384	0.0786558405	C	6.6198824863	4.4697027175	0.4727884705
H	-4.0931994617	-4.0766189503	-2.5814085370	C	4.9301267169	4.9463323866	-1.2827044546
H	-4.3601445766	-1.9437796184	-1.4383008878	H	6.5113967674	3.2133829417	-2.7349861378
C	-3.0560953469	-4.1239567324	-2.2818493289	H	7.6505571417	2.8095618067	-1.4530136549
C	-0.3822042840	-4.2103604885	-1.5188904561	H	7.5410025679	4.4682520148	-2.0497937023
C	-0.9095448951	-5.1935583362	-2.3094538450	H	7.3441412514	3.7654513399	0.8756938263
C	-2.2613642929	-5.1601534682	-2.6828477905	H	7.1544588148	5.3732814433	0.1857754657
H	0.6612303956	-4.2376927608	-1.2448707102	H	5.9070352340	4.7337787195	1.2501942210
H	-0.2835071899	-6.0029188439	-2.6548037665	H	4.1552283377	5.1433860146	-0.5450048040
H	-2.6629513677	-5.9510261216	-3.2990157215	H	5.4581192623	5.8753267443	-1.4886852446
C	1.5147384657	-1.0171049220	-0.0560224383	H	4.4501603408	4.6168920210	-2.1999886199
C	0.7201637527	-2.0998903530	0.2490704959	H	4.4382372800	2.3675993270	-2.4477007229
C	1.2460545226	-3.1497992377	1.0512781791	H	3.1588887406	0.3509601849	-2.0050671385
C	2.6033440590	-3.0786500784	1.4624898095	H	4.3150243006	0.5173696512	2.0972624652
C	3.3788507371	-1.9588270447	1.1073311121	H	5.6068942647	2.5223761384	1.6629030041
C	2.8553994943	-0.9195354680	0.3766595521	C	-3.6307540749	0.2422372180	-0.0509941669
H	-0.5848703409	-4.2691016707	1.2286656386	C	-3.6929954077	0.7720001697	1.2318036196
C	0.4597374928	-4.2327393037	1.4975688267	C	-4.4466499981	1.9019497407	1.4942461119
C	3.1365685486	-4.1236396251	2.2471697553	C	-5.1602374862	2.5460820110	0.4909174745
H	4.4080299046	-1.9133232946	1.4326847719	C	-5.0949784758	2.0044053256	-0.7913418840
C	2.3574040494	-5.1761750974	2.6360246605	C	-4.3503829543	0.8748626135	-1.0607315991
C	1.0040858793	-5.2214917631	2.2694043758	C	-5.9599675266	4.2500965653	2.1962510168
H	4.1744719085	-4.0674401297	2.5421248685	C	-5.9899178123	3.7967380380	0.7343007131
H	2.7725399530	-5.9711826538	3.2377012214	C	-7.4494466144	3.5133557707	0.3512887059
H	0.3905401504	-6.0443126051	2.6052802809	C	-5.4367010529	4.9374451352	-0.1317740476
O	-0.9631592662	0.0095576981	0.8345392432	H	-4.9472087218	4.4902208524	2.5112047200
O	0.9635366100	0.0303121855	-0.7840950423	H	-6.3656612910	3.4831095603	2.8519602993
P	0.0377508313	1.0076310692	0.0959431815	H	-6.5683107639	5.1452446353	2.3103725976
O	0.6984694602	1.9311905854	1.0053091900	H	-7.5407149652	3.2487499408	-0.6984570047
C	-2.8245879451	-0.9471079965	-0.3600528342	H	-7.8406239004	2.6901702893	0.9456706990
C	-3.3314867930	-1.9804502789	-1.1099871997	H	-8.0623622356	4.3933973225	0.5347158908
C	3.6416092994	0.2855542781	0.0810681226	H	-5.4924198100	4.6953675510	-1.1894541357
C	3.6902814352	0.8315805595	-1.1972252202	H	-6.0066204239	5.8483015037	0.0397009263
C	4.4238905546	1.9743220621	-1.4419215636	H	-4.3956604523	5.1287308578	0.1202504031

H	-4.4643536520	2.2734863960	2.5056885750	H	-4.3043874822	0.4882773435	-2.0686685312
H	-3.1561133424	0.2917649530	2.0356877483	H	-5.6315692531	2.4713718585	-1.6039591634

CPA-**4**

O	0.7992872245	-2.4663014575	-0.9684329973	C	-2.3069664818	-0.8853486631	-3.7648909720
H	0.7052500556	-3.4076013807	-0.8293558521	H	-6.1741564150	-1.9201353853	2.4972436751
C	2.7055275363	2.5360537964	-1.0209151531	H	-5.8961014886	-0.2319491007	2.0769060229
C	1.2998575237	2.5206330248	-0.8256590465	H	-5.3916066620	-0.8426122739	3.6552775670
C	0.7142464399	1.4279185211	-0.1223544987	H	-3.4298878386	-0.6151267439	2.1832429752
C	1.5065127795	0.3592143215	0.2294023568	H	-4.1722407384	-3.5230766711	2.7229397115
H	4.3687954547	3.6394339874	-1.8119007856	H	-2.5533335245	-2.9196578487	2.3471802451
H	4.5483491198	1.4662679260	-0.7316320531	H	-3.3733382999	-2.4033408003	3.8240586585
C	3.2959446882	3.6323952626	-1.6839859673	H	-5.3583530994	-3.5207223852	0.8373114989
C	0.5333265353	3.5716961131	-1.3713730408	H	-5.6016649113	-4.0058996104	-1.5514304175
C	1.1343145099	4.6113243860	-2.0257103109	H	-4.6474735334	-2.4842879777	-3.2203433087
C	2.5287261268	4.6535728842	-2.1689299857	H	-2.7678635336	0.6353300287	-2.3383905636
H	-0.5412732194	3.5428938114	-1.2778724518	H	-5.0060647784	-0.3505125827	-4.1612696691
H	0.5336523562	5.4069001015	-2.4409265794	H	-5.1531149792	0.9205127554	-2.9475078239
H	2.9871122570	5.4884818955	-2.6778647030	H	-4.0888793868	1.1458019310	-4.3393661817
C	-1.4990422136	0.3430678328	-0.1757472102	H	-1.9030722500	-0.1728889625	-4.4816781121
C	-0.7245576654	1.4152092497	0.2069301436	H	-1.4843973523	-1.3024373946	-3.1891742627
C	-1.3326166238	2.4885739534	0.9198105549	H	-2.7886382853	-1.6894881343	-4.3157139484
C	-2.7405086549	2.4806661684	1.0995127301	C	3.6789827215	-0.8663219969	0.3511606276
C	-3.4958705898	1.3939471517	0.6161608428	C	3.8500846100	-1.2135497194	1.6955814218
C	-2.8969859441	0.3117988295	0.0234970561	C	4.5427253111	-2.3757239177	2.0057670999
H	0.4903597069	3.5252692296	1.4105741760	C	5.0602707597	-3.1790990391	1.0091242032
C	-0.5854102065	3.5375654790	1.4954875321	C	4.9064561442	-2.8171048823	-0.3146436619
C	-3.3515394893	3.5526198747	1.7835305676	C	4.2180760185	-1.6636710743	-0.6665136893
H	-4.5681358537	1.4044775950	0.7444505274	C	5.4830692036	-0.9320891263	-2.6832985374
C	-2.6026573263	4.5714655055	2.3009754782	C	4.1014234136	-1.2937849023	-2.1289547942
C	-1.2065682014	4.5527448988	2.1689104903	C	3.4713637139	-2.4168581754	-2.9546386503
H	-4.4254372193	3.5422417309	1.9013343162	C	3.3589402959	-0.3229372251	2.8138009364
H	-3.0770151933	5.3867923557	2.8267419966	C	4.5531206133	0.2518425073	3.5822484838
H	-0.6209745800	5.3464062848	2.6086316649	C	2.4150529097	-1.0738358103	3.7544808683
O	0.9520464285	-0.7233694391	0.8938823334	H	6.1473310875	-1.7924988425	-2.6546675659
O	-0.9031284366	-0.7366327340	-0.8145325492	H	5.9356905527	-0.1351835167	-2.0973361151
P	-0.0499152565	-1.7013941975	0.1375616065	H	5.3983790252	-0.5969873595	-3.7148738653
O	-0.7589220415	-2.5760018904	1.0596795265	H	3.4551525240	-0.4171068716	-2.2253365996
C	2.9001780962	0.3418004378	-0.0056028188	H	4.0752915651	-3.3201531829	-2.9263866269
C	3.4789841796	1.4433551451	-0.5816013360	H	2.4804486736	-2.6460575254	-2.5734333367
C	-3.6660583054	-0.8766053795	-0.4116511862	H	3.3783985994	-2.1025846507	-3.9922827304
C	-3.8204901219	-1.1365144396	-1.7774255433	H	5.3321832238	-3.4423206005	-1.0848821642
C	-4.5194773506	-2.2693993798	-2.1699911718	H	5.5944045617	-4.0829732475	1.2643549520
C	-5.0609887902	-3.1263829087	-1.2322662280	H	4.6838761714	-2.6564242523	3.0382965557
C	-4.9195839892	-2.8514752105	0.1133930698	H	2.8062645032	0.5173554515	2.3841403106
C	-4.2223257007	-1.7317338846	0.5466368296	H	5.2106265511	0.7976684883	2.9083786915
C	-5.4788792380	-1.0902416487	2.5995617493	H	4.2074099854	0.9340542671	4.3559623703
C	-4.1059690824	-1.4609811390	2.0291185323	H	5.1286193082	-0.5401220002	4.0551105782
C	-3.5161478244	-2.6581801579	2.7759108586	H	2.9298223668	-1.8852015806	4.2629083550
C	-3.2961418100	-0.1863813354	-2.8307390922	H	2.0234685696	-0.3932347236	4.5078137059
C	-4.4606511744	0.4186862058	-3.6203177699	H	1.5798257504	-1.4883915822	3.1952122712

CPA-**5**

O	-0.6354591842	0.8048173314	-0.9967802787	C	-0.6625940682	-3.1037170338	-0.3172754496
H	-0.6267964992	1.7390620463	-0.7935601610	C	-1.5204063200	-2.0544399770	-0.0802089017
C	-2.4172701762	-4.1591332259	-1.6612002528	H	-3.8734307567	-5.2167110014	-2.8318778305
C	-1.0819233472	-4.1576005269	-1.1801998470	H	-4.2873927822	-3.0990209241	-1.7084783566

C	-2.8498767643	-5.2189978179	-2.4856864496
C	-0.2112280768	-5.1813770919	-1.6087878019
C	-0.6565703498	-6.1850361683	-2.4239616810
C	-1.9914083227	-6.2163667089	-2.8528437705
H	0.8208830822	-5.1599762024	-1.2946598568
H	0.0228407940	-6.9601529275	-2.7461028827
H	-2.3288171221	-7.0232632861	-3.4863706625
C	1.5005690468	-2.0119230060	0.1362097550
C	0.6790243649	-3.1049925548	0.2981558145
C	1.1427576010	-4.2110636937	1.0672538199
C	2.4829123152	-4.2067427453	1.5347132813
C	3.3054817190	-3.0932085420	1.2734494681
C	2.8264043983	-1.9850004728	0.6227000929
H	-0.7269952847	-5.2787240041	1.1187132110
C	0.3081764406	-5.2911393531	1.4235619086
C	2.9549927622	-5.3100247146	2.2764803096
H	4.3275764871	-3.1046008758	1.6221056249
C	2.1304614184	-6.3571910939	2.5766669342
C	0.7915575036	-6.3371958653	2.1597817936
H	3.9812699229	-5.3013784964	2.6141316311
H	2.4979322813	-7.1967129094	3.1480218272
H	0.1394116715	-7.1548148246	2.4288950529
O	-1.1217888398	-1.0094490433	0.7388992322
O	1.0362191036	-0.9028684315	-0.5587640716
P	-0.0114889112	0.0123966391	0.2332540483
O	0.4757562389	0.8645507507	1.3080388833
C	-2.8346749100	-2.0187244189	-0.5989243546
C	-3.2729378607	-3.0874997762	-1.3379549795
C	3.6475958981	-0.7712968554	0.4195956171
C	4.0740515297	-0.4226485093	-0.8653148730
C	4.8124822888	0.7357324864	-1.0388332525
C	5.1515169678	1.5656354896	0.0225610944
C	4.7352934209	1.1848718590	1.2879330315
C	3.9873720303	0.0357651978	1.5076172350
C	3.7981928533	-1.3096582196	-2.0592992147
C	3.0124616707	-0.5633799316	-3.1387072237
C	5.1117863788	-1.8617337659	-2.6206339911
C	5.9517925271	2.8308044779	-0.2416631761
C	6.2451775885	3.6270942385	1.0356106718
C	7.0440898492	4.8896459036	0.7111828697
C	6.2441209822	5.7746976408	-0.2414970280
C	5.9631519406	4.9965581523	-1.5244329122
C	7.2846259366	4.6028311655	-2.1829033429
C	8.0892504180	3.7286847508	-1.2220598592
C	8.3712181973	4.5067442129	0.0606761736
C	7.2957133499	2.4653830063	-0.8927473684
C	5.1600968917	3.7390403298	-1.1965610646
C	3.5787134997	-0.3104683109	2.9216603567
C	2.8115309019	0.8357432465	3.5825338495
C	4.8135411848	-0.6838617320	3.7479457449
H	3.1933724705	-2.1616829757	-1.7353515408
H	2.7728424911	-1.2369048866	-3.9593925473
H	2.0844633180	-0.1793103246	-2.7225843412
H	3.5856966424	0.2701612525	-3.5372612303
H	4.9112546342	-2.5466677671	-3.4420263746
H	5.7457972998	-1.0596923158	-2.9903541823
H	5.6565624476	-2.3990144680	-1.8470583509
H	5.1335812862	0.9923363075	-2.0365699644
H	6.8199004021	3.0117567417	1.7317295815
H	5.3069480809	3.9123558834	1.5163836518
H	7.2385679544	5.4374278339	1.6410268560
H	6.8069421166	6.6830431082	-0.4696080571
H	5.3029995898	6.0718529090	0.2270726805
H	5.3847736739	5.6233584142	-2.2136360860
H	7.8555532313	5.4991493866	-2.4368870569
H	7.0907042993	4.0567719415	-3.1093790522
H	9.0374787532	3.4449698445	-1.6941013142
H	8.9499143773	5.4054289979	-0.1658101811
H	8.9600821268	3.8930719485	0.7465926489
H	7.1295183040	1.8948803093	-1.8076659111
H	7.8667026688	1.8326688005	-0.2084716780
H	4.9298991500	3.2053174929	-2.1198744283
H	4.2112933314	4.0109395324	-0.7275356454
H	4.9879192688	1.7901686850	2.1413626068
H	2.9107453658	-1.1761252285	2.8940364250
H	2.4542779847	0.5233410492	4.5617405346
H	3.4407446348	1.7122050565	3.7145769181
H	1.9566372332	1.1056044183	2.9696945970
H	5.3544549027	-1.5057689785	3.2834285964
H	4.5169093147	-0.9878264870	4.7495537215
H	5.4907281868	0.1628311369	3.8323564597
C	-3.6719317845	-0.8234511580	-0.3559344076
C	-4.0009274058	0.0248176560	-1.4201725368
C	-4.7352666718	1.1701758929	-1.1592740526
C	-5.1586568510	1.5121480452	0.1190397805
C	-4.8431835755	0.6406211504	1.1488943941
C	-4.1100190758	-0.5184154625	0.9342032855
C	-3.6088277996	-0.2840496339	-2.8489898656
C	-4.8609190796	-0.6100560970	-3.6693709159
C	-2.8295082354	0.8630255239	-3.4945481480
C	-5.9432174119	2.7967385881	0.3295954958
C	-6.3297232074	3.0244920462	1.7959771402
C	-7.1099610859	4.3302997337	1.9500264502
C	-8.3861141848	4.2688472189	1.1140724735
C	-8.0109685012	4.0600787680	-0.3508686385
C	-7.1453983805	5.2256162441	-0.8273699536
C	-5.8756543408	5.2939549215	0.0199501192
C	-6.2499409834	5.5028829841	1.4850322370
C	-5.0907409280	3.9916947859	-0.1281279328
C	-7.2359053899	2.7522065954	-0.5013617451
C	-3.8657777334	-1.4561660768	2.0950723212
C	-5.1951602208	-2.0514136201	2.5696747020
C	-3.1403433343	-0.7550324176	3.2446924425
H	-2.9581335531	-1.1626399009	-2.8570103674
H	-5.5156768913	0.2556868798	-3.7349383013
H	-4.5833264556	-0.9083380889	-4.6781569775
H	-5.4199715442	-1.4222863390	-3.2101009825
H	-3.4233622111	1.7721338250	-3.5450204582
H	-1.9274748572	1.0654313665	-2.9244220351
H	-2.5437319656	0.5886174557	-4.5080732576
H	-4.9848561103	1.8147193756	-1.9876722718
H	-5.4293075992	3.0714613154	2.4125551716
H	-6.9500377485	2.1970845706	2.1483192664
H	-7.3719233048	4.4664489866	3.0060062496
H	-8.9522019856	5.1963842486	1.2271221888
H	-9.0183795303	3.4465292329	1.4573655438
H	-8.9221589467	4.0097972631	-0.9586658029
H	-7.7016467643	6.1618389609	-0.7400862116
H	-6.8840023397	5.0894516071	-1.8796448874
H	-5.2533800262	6.1294760473	-0.3218990240
H	-6.8004208852	6.4393936462	1.6012437853
H	-5.3456290237	5.5674464518	2.0945848528
H	-4.7928680862	3.8655300726	-1.1702084346
H	-4.1792017284	4.0334275917	0.4735107612

H	-7.0025775913	2.5899195811	-1.5546888011	H	-5.0213579999	-2.7656207041	3.3717251219
H	-7.8500060699	1.9125322121	-0.1661348730	H	-5.8587660164	-1.2739613461	2.9402490762
H	-5.1708731393	0.8516348580	2.1524011341	H	-2.2106887906	-0.3173267096	2.8893416793
H	-3.2354391858	-2.2832385154	1.7563528870	H	-2.9075041648	-1.4727637468	4.0287468703
H	-5.6938887798	-2.5656368936	1.7504589701	H	-3.7533490397	0.0324397539	3.6760248324

Detailed Statistical Analyses

The statistical analyses reported within the main text were limited to comparisons between only a few pairs of descriptors. We show here additional analyses that support the expectation that *active site-based* buried volume is an *advantageous*, *best-in-general* representation relative to other features, including: (1) expanded confidence intervals (ref. **Fig. 2** from main text), (2) average statistical performance for other representations of confinement across structural diversity (ref. **Fig. 3** from main text), (3) functional diversity (ref. **Fig. 4** from main text), (4) mechanistic diversity (ref. electronic control in **Fig. 5** from main text), and (5) univariate regression models for each dataset built from *active site-based* buried volume.

Expanded Confidence Intervals for Meaningful Correlations

Initially, *active site-based* buried volume was assigned as the *most meaningful* representation as it captured the greatest proportion of *meaningful* univariate linear regressions (based on *p*-value, ref. *dataset_scores.xlsx*) across all datasets considered (54 total reactions). In the main text (**Fig. 2B**), this analysis was conducted with the standard 95% confidence and 99% confidence. We report a complete analysis (up to 99.9% confidence) below for all descriptors considered.

Across the analyses, we observe qualitatively consistent (if not more robust) conclusions at increasingly strict confidence levels. Even at the most strict confidence, *active site-based* buried volume captures the greatest proportion of *meaningful* univariate regressions, and a much greater proportion compared to the average descriptor altogether.

	confidence level				
molecular descriptor	**95%**	**99%**	**99.50%**	**99.90%**	
**active site-based sterimol_L**	0.19	0.09	0.09	0.04	1
**active site-based sterimol_Bmin**	0.33	0.11	0.06	0.02	2
**active site-based sterimol_Bmax**	0.3	0.07	0.02	0.02	3
**active site-based xtb_pKa**	0.11	0.09	0.06	0	4
**active site-based bur_vol aggregate**	**0.67**	**0.48**	**0.41**	**0.26**	5
					6
**substituent-based sterimol_L**	0.09	0.02	0.02	0	7
**substituent-based sterimol_Bmin**	0.17	0.06	0.06	0.04	8
**substituent-based sterimol_Bmax**	0.24	0.09	0.09	0.06	9
**substituent-based xtb_atom_charge**	0.52	0.3	0.22	0.07	10
**substituent-based isotropic_nmr**	0.56	0.3	0.2	0.09	11
**substituent-based anisotropic_nmr**	0.61	0.31	0.24	0.11	12
**substituent-based mulliken_charge**	0.61	0.26	0.15	0.11	13
**substituent-based nbo_charge**	0.5	0.33	0.2	0.04	14
**substituent-based chelpg_charge**	0.65	0.33	0.2	0.07	15
**substituent-based hirshfeld_charge**	0.57	0.33	0.22	0.11	16
**substituent-based cm5_charge**	0.59	0.24	0.19	0.09	17
**substituent-based bur_vol**	0.7	0.39	0.35	0.19	18
average	0.44	0.22	0.16	0.08	

Supplementary Fig. 1. Indexed and tabulated proportions of meaningful regressions across all datasets by each descriptor considered, from 95% to 99.9% confidence.

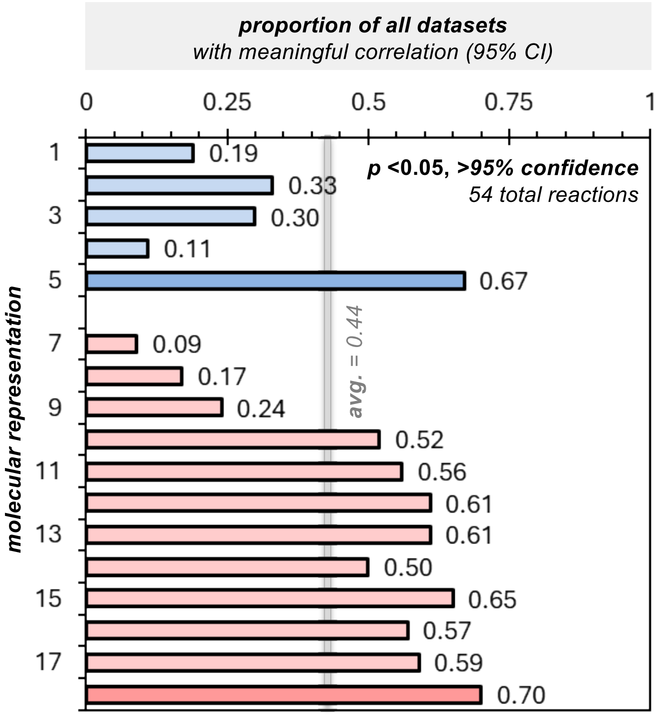

Supplementary Fig. 2. Proportion of datasets (54 total reactions) captured with a *meaningful* univariate linear regression model by the corresponding descriptors (at 95% confidence).

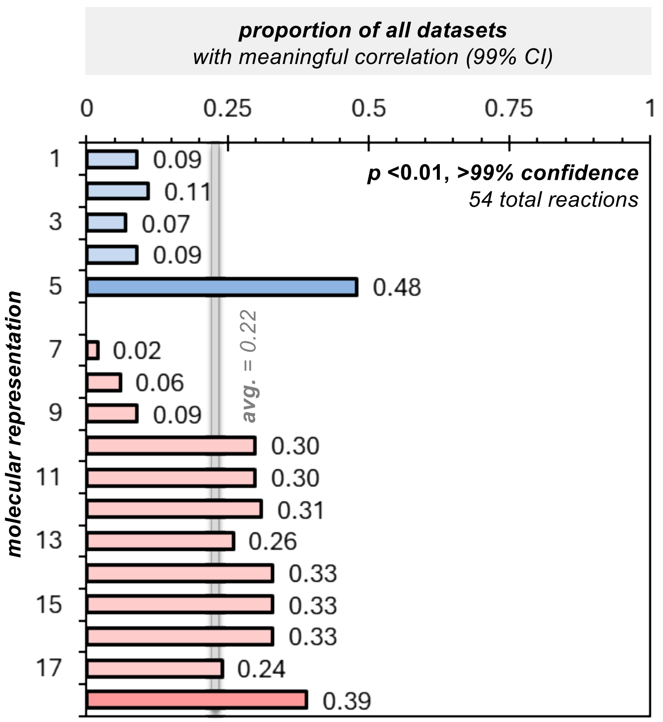

Supplementary Fig. 3. Proportion of datasets (54 total reactions) captured with a *meaningful* univariate linear regression model by the corresponding descriptors (at 99% confidence).

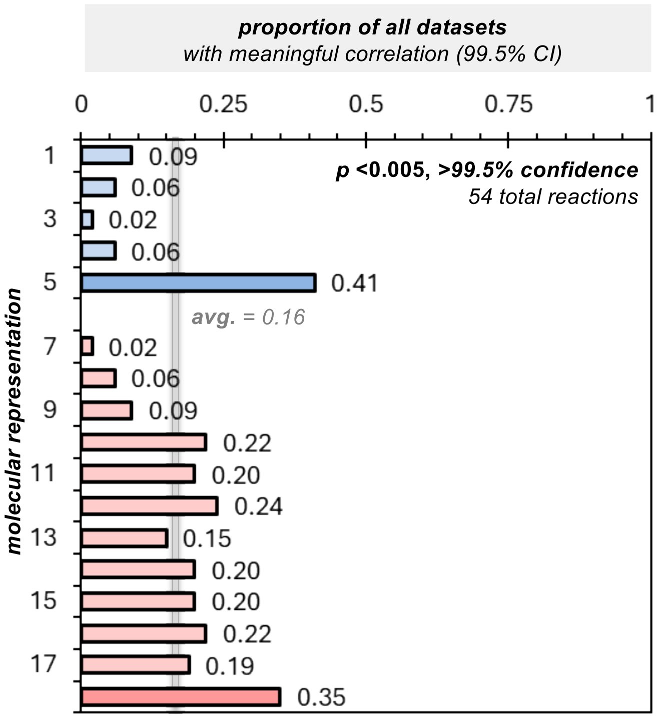

Supplementary Fig. 4. Proportion of datasets (54 total reactions) captured with a *meaningful* univariate linear regression model by the corresponding descriptors (at 99.5% confidence).

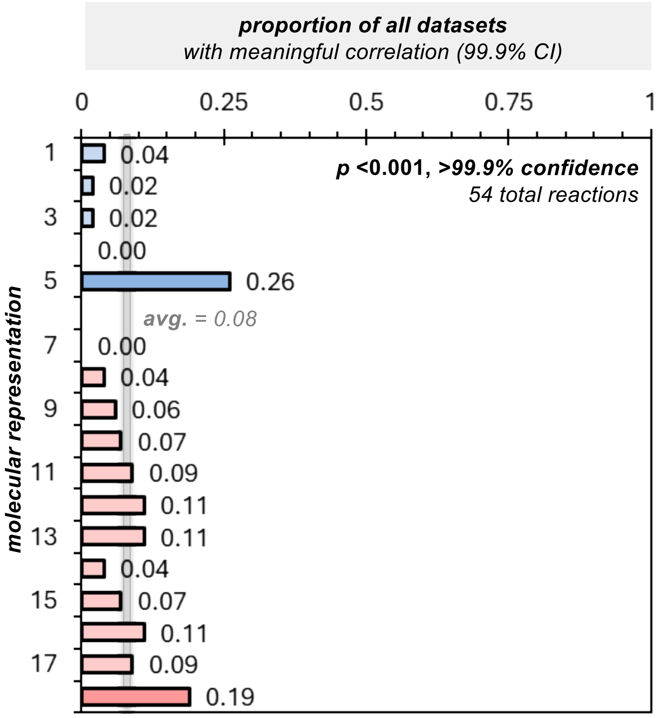

Supplementary Fig. 5. Proportion of datasets (54 total reactions) captured with a *meaningful* univariate linear regression model by the corresponding descriptors (at 99.9% confidence).

Regression Performance over Structurally Diverse Datasets

Active site-based buried volume and *substituent-based* buried volumes were explicitly juxtaposed in the main text (**Fig. 3**). The two are distinguished solely by their anticipated capacity to reflect *structural diversity* as it is reflected by the construction of chiral acid organocatalysts. We identify three prominent structural motifs (substituent, chiral backbone, and active site) in BAOCs. As *substituent-based* representations should reflect changes in the substituent, we purposefully group datasets that display changes primarily to this motif alone and denote these as *limited diversity*. The remaining datasets display changes at other motifs as well and we denote these as *significant diversity*.

The qualitative distinction of *limited* and *significant diversity* are distinguished by whether there are more than one examples of BAOCs with structural motifs changed at the chiral backbone or active site (relative to the majority). As this qualitative definition fails to reflect dataset size, we invoke a *quantitative* distinction defined by the Gini-Simpson index. The index is essentially *the likelihood of the two samples drawn from the dataset bearing different structural motifs* (as defined by a different chiral backbone or active site). We labeled each dataset with its corresponding *qualitative* definition and then compared them to their *quantitative* Gini-Simpson index. Excellent agreement (F1 = 0.96) between the two categories was observed with a

univariate classification at Gini-Simpson = 0.30 to distinguish *limited* and *significant diversity*. This *quantitative* analysis only *misclassified* two datasets – R-**X** and R-**XXIII**. Each dataset possesses two instances of a BAOC with a different chiral backbone, although the datasets are relatively large (15 ex. and 14 ex. respectively, compared to the average size of 11). As such, we agree with the classification that such sparse diversity should be considered *limited diversity*.

Altogether, the full classification follows:

Limited diversity – R-**I** through R-**XXVII**.

Significant diversity – R-**XXVIII** through R-**LIV**.

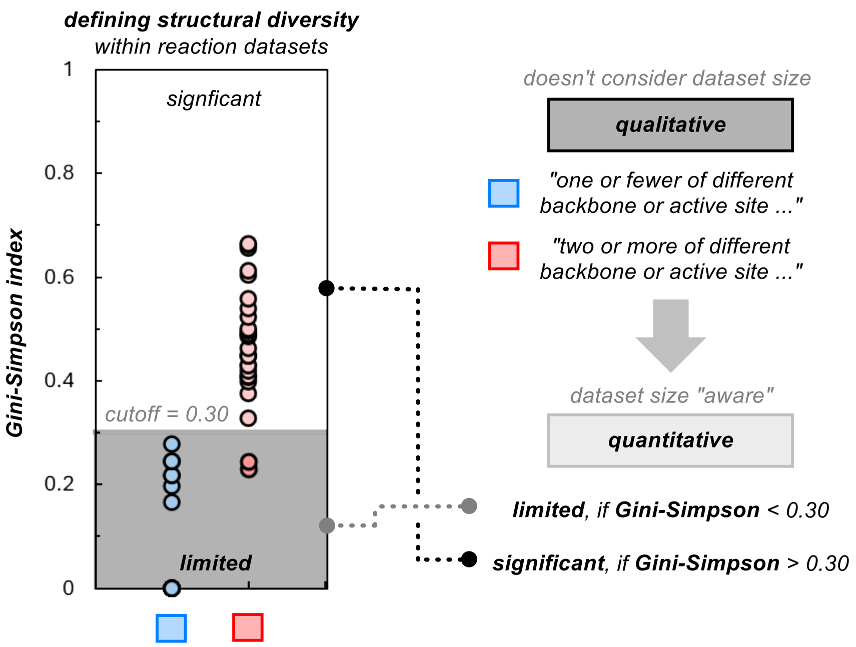

Supplementary Fig. 6. Univariate classification of *structural diversity* with calculated Gini-Simpson index. Classifier defines *limited diversity* if the corresponding Gini-Simpson index is less than 0.30, and *significant diversity* if it is greater than 0.30 (F1 = 0.96).

In the main text, only *active site-based* and *substituent-based* buried volumes were showcased in their performance across the *limited* and *significant diversity* datasets. We showcase other competing representations of confinement below, highlighting their inadequate performance and the relative power of *active site-based* buried volume to accommodate BAOC structural diversity in relationships to stereoselectivity.

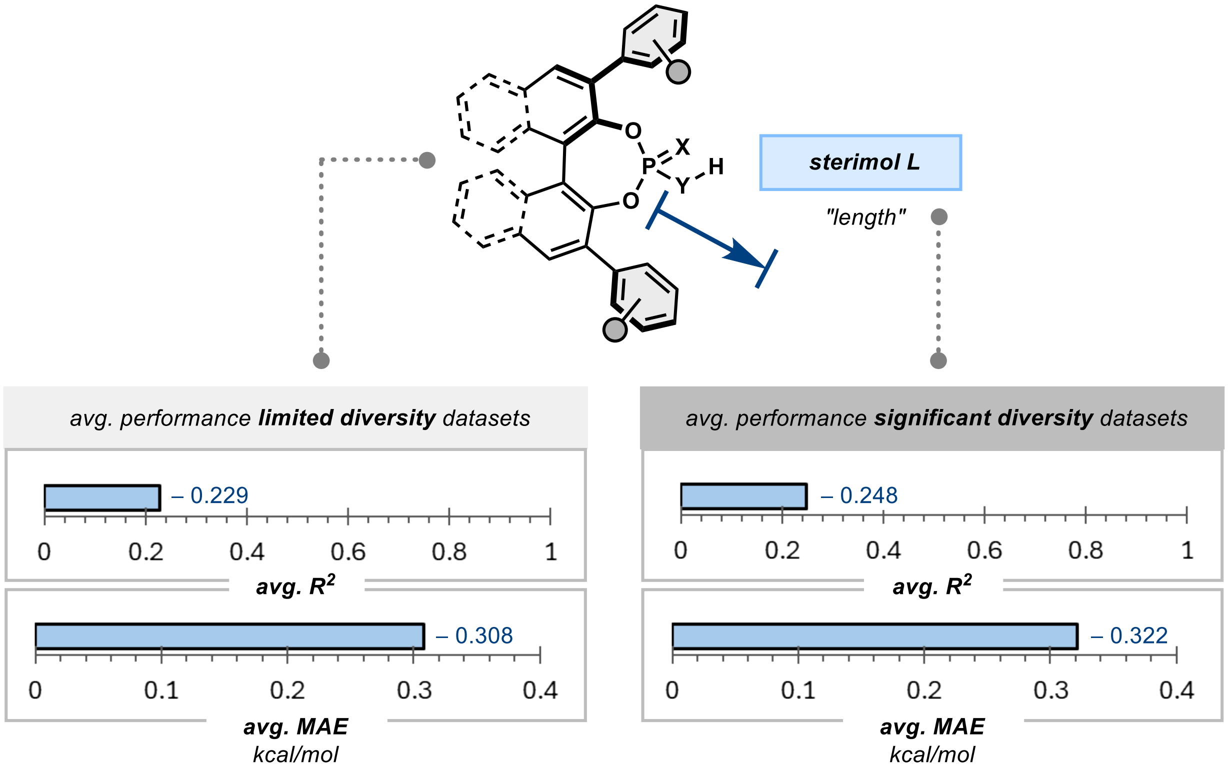

Supplementary Fig. 7. Average performance (R^2 and MAE in *kcal/mol*) across *limited diversity* (left) and *significant diversity* datasets by *active site-based* sterimol L.

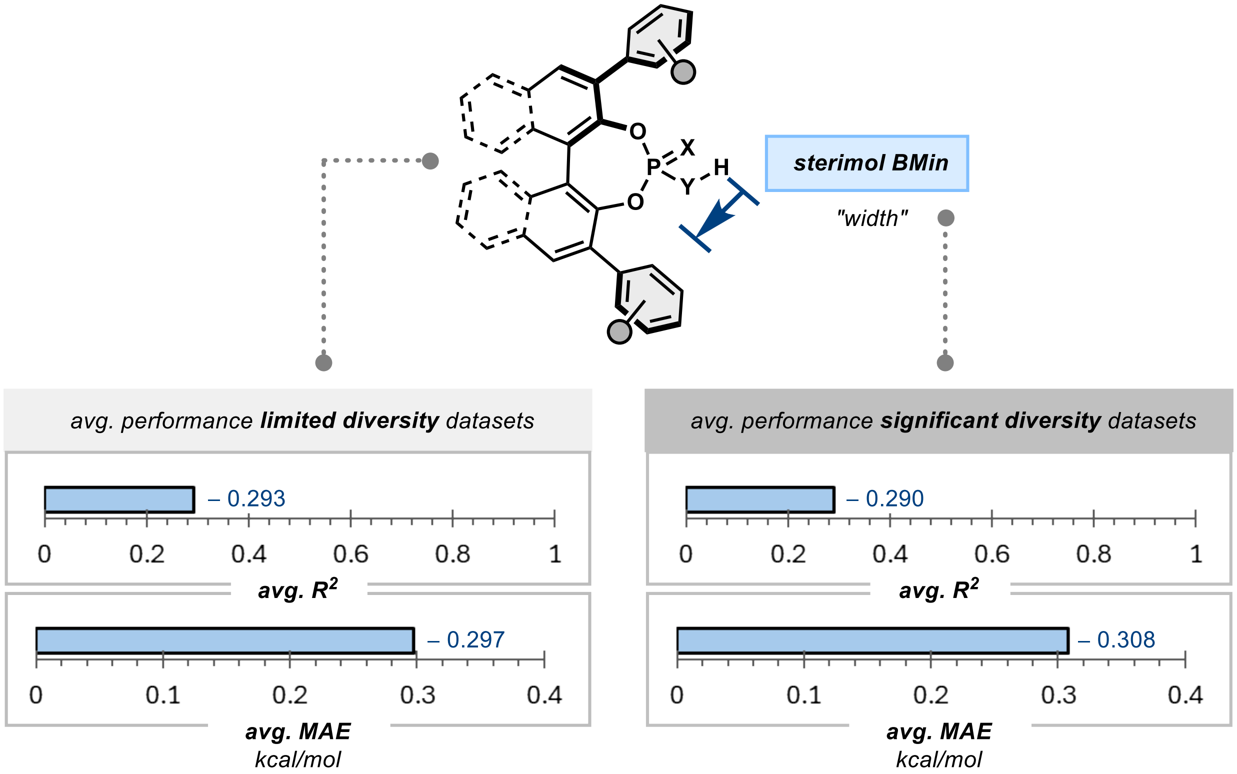

Supplementary Fig. 8. Average performance (R^2 and MAE in *kcal/mol*) across *limited diversity* (left) and *significant diversity* datasets by *active site-based* sterimol BMin.

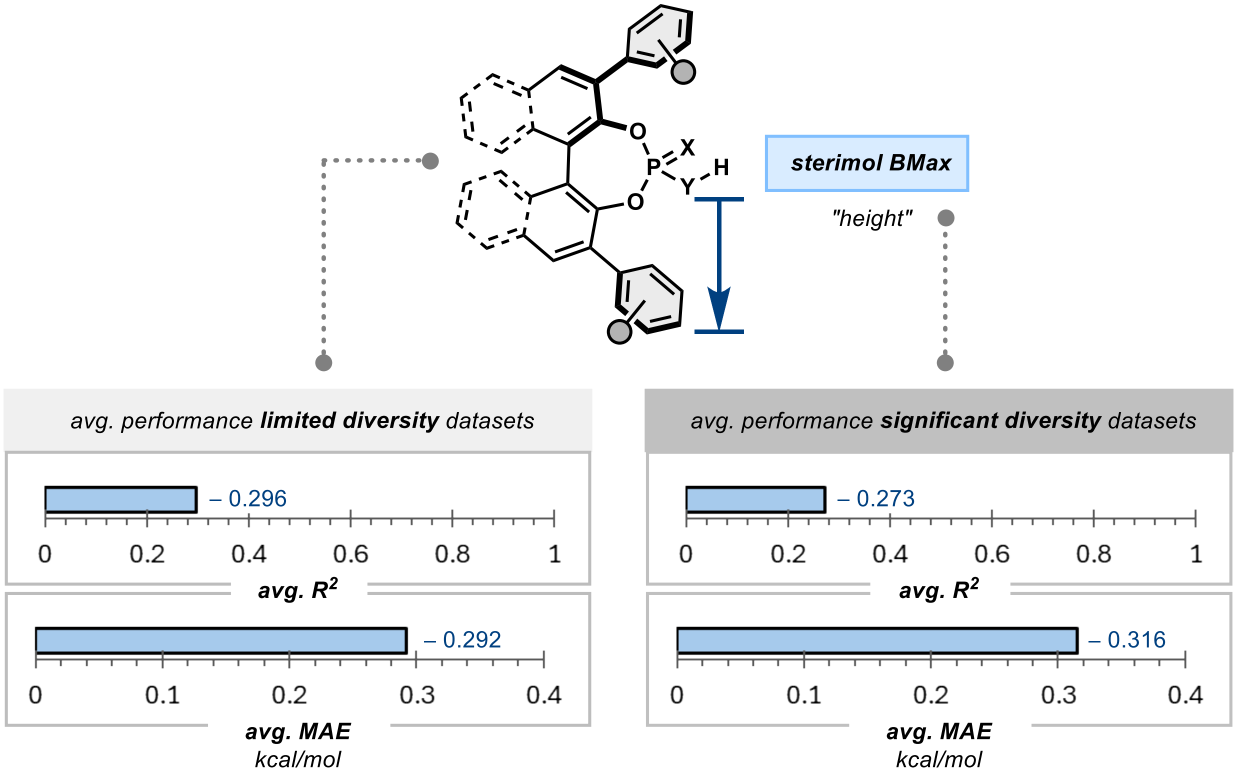

Supplementary Fig. 9. Average performance (R^2 and MAE in *kcal/mol*) across *limited diversity* (left) and *significant diversity* datasets by *active site-based* sterimol BMax.

Regression Performance over Functionally Diverse Datasets

In the main text, only *active site-based* buried volume was showcased in its performance across the three classifications of *functional diversity – acid-mediated additions, acid-mediated substitutions and eliminations,* and *complex/multicomponent reaction* datasets. These categories are defined as described in the text and contain the following datasets accordingly:

Acid-mediated additions – R-**II**, R-**III**, R-**IV**, R-**VII**, R-**VIII**, R-**IX**, R-**X**, R-**XII**, R-**XV**, R-**XVII**, R-**XVIII**, R-**XIX**, R-**XX**, R-**XXIV**, R-**XXVI**, R-**XXVIII**, R-**XXIX**, R-**XXX**, R-**XXXII**, R-**XXXIII**, R-**XXXVIII**, R-**XXXIX**, R- **XLIII**, R-**XLV**, R-**XLVIII**, R-**LIII**, R-**LIV**

Acid-mediated substitutions/eliminations – R-**I**, R-**VI**, R-**XI**, R-**XXI**, R-**XXII**, R-**XXV**, R-**XXVII**, R-**XXXI**, R-**XXXIV**, R-**XLII**, R-**XLIV**, R-**XLVI**, R-**XLVII**, R-**L**, R-**LII**

Complex/multicomponent reactions – R-**V**, R-**XIII**, R-**XIV**, R-**XVI**, R-**XXIII**, R-**XXXV**, R-**XXXVI**, R-**XXXVII**, R-**XL**, R-**XLI**, R-**XLIX**, R-**LI**

Below, we showcase other representations of confinement (both *active site-based* and *substituent-based* representations). These results highlight the utility of *active site-based* buried volume to accommodate BAOC functional diversity in relationships to stereoselectivity.

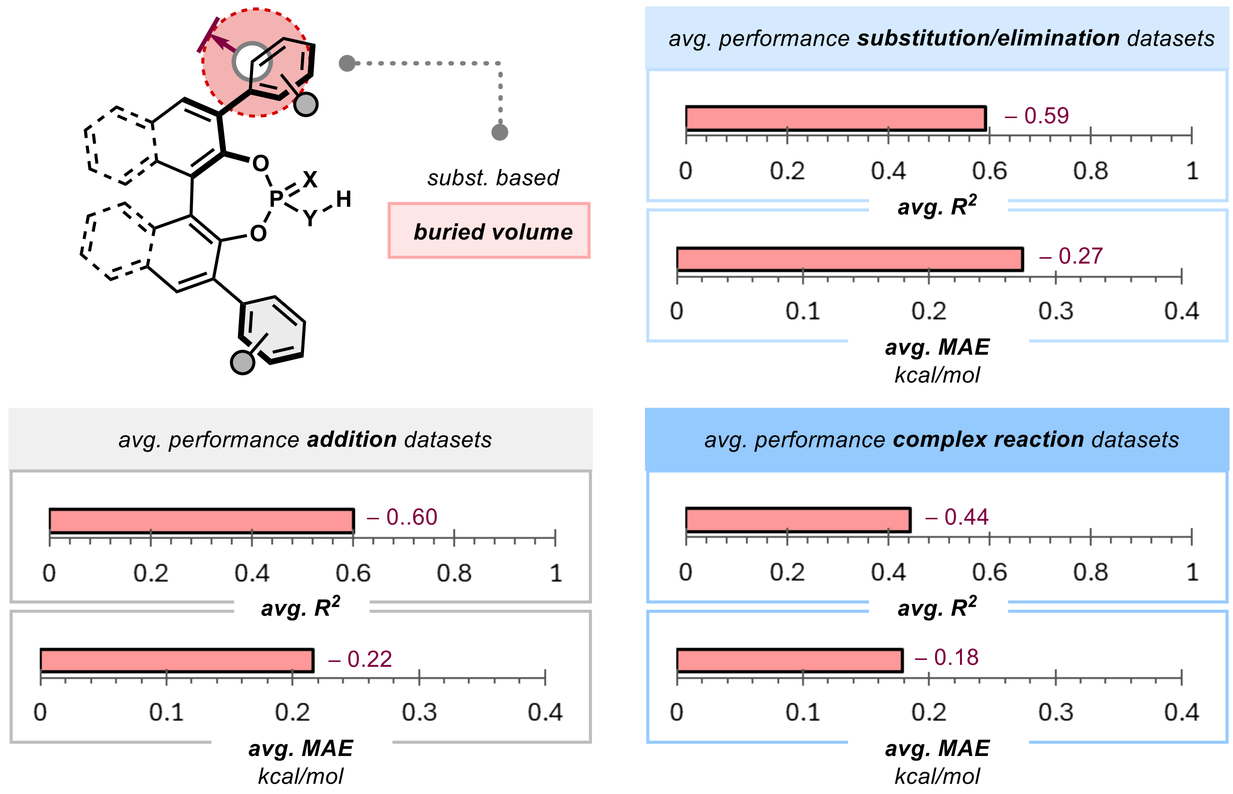

Supplementary Fig. 10. Average performance (R^2 and MAE in *kcal/mol*) across *acid-mediated addition* (bottom left), *acid-mediated substitutions and eliminations* (top right), and *complex/multicomponent reaction* (bottom right) datasets by *substituent-based* buried volume.

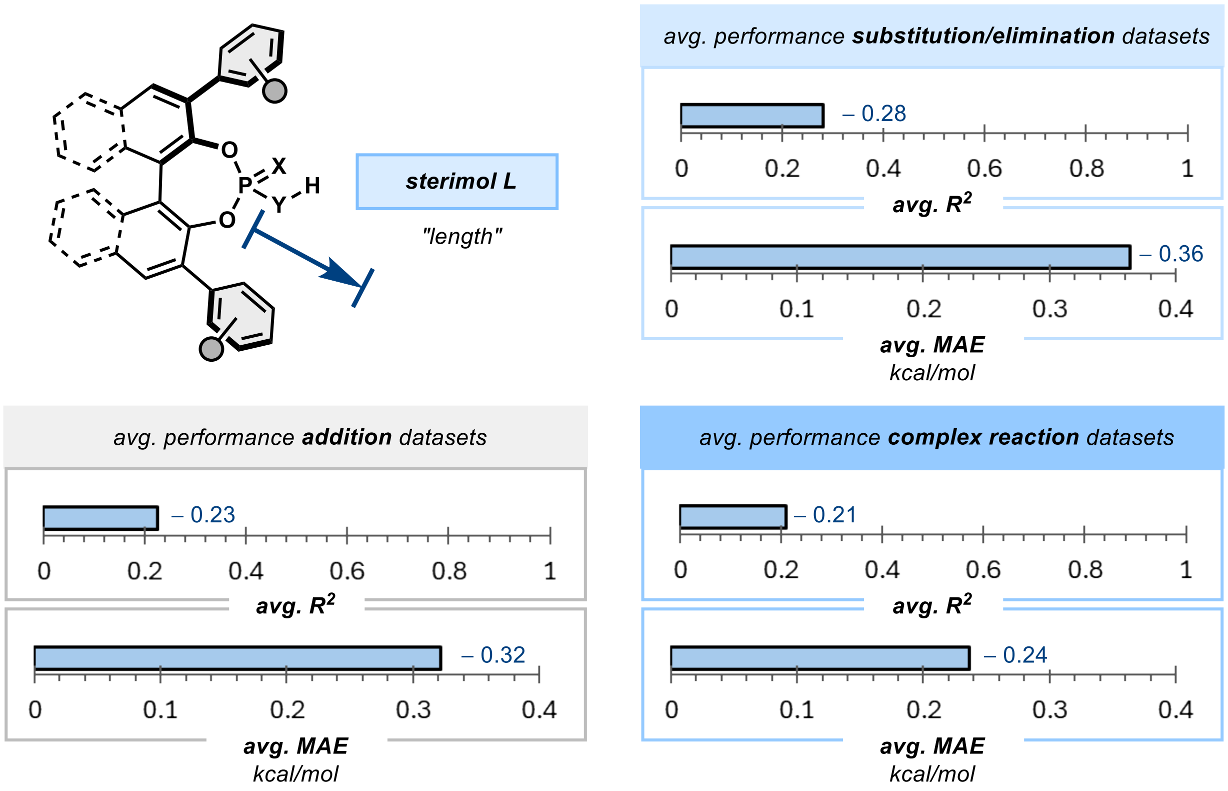

Supplementary Fig. 11. Average performance (R^2 and MAE in *kcal/mol*) across *acid-mediated addition* (bottom left), *acid-mediated substitutions and eliminations* (top right), and *complex/multicomponent reaction* (bottom right) datasets by *active site-based* sterimol L.

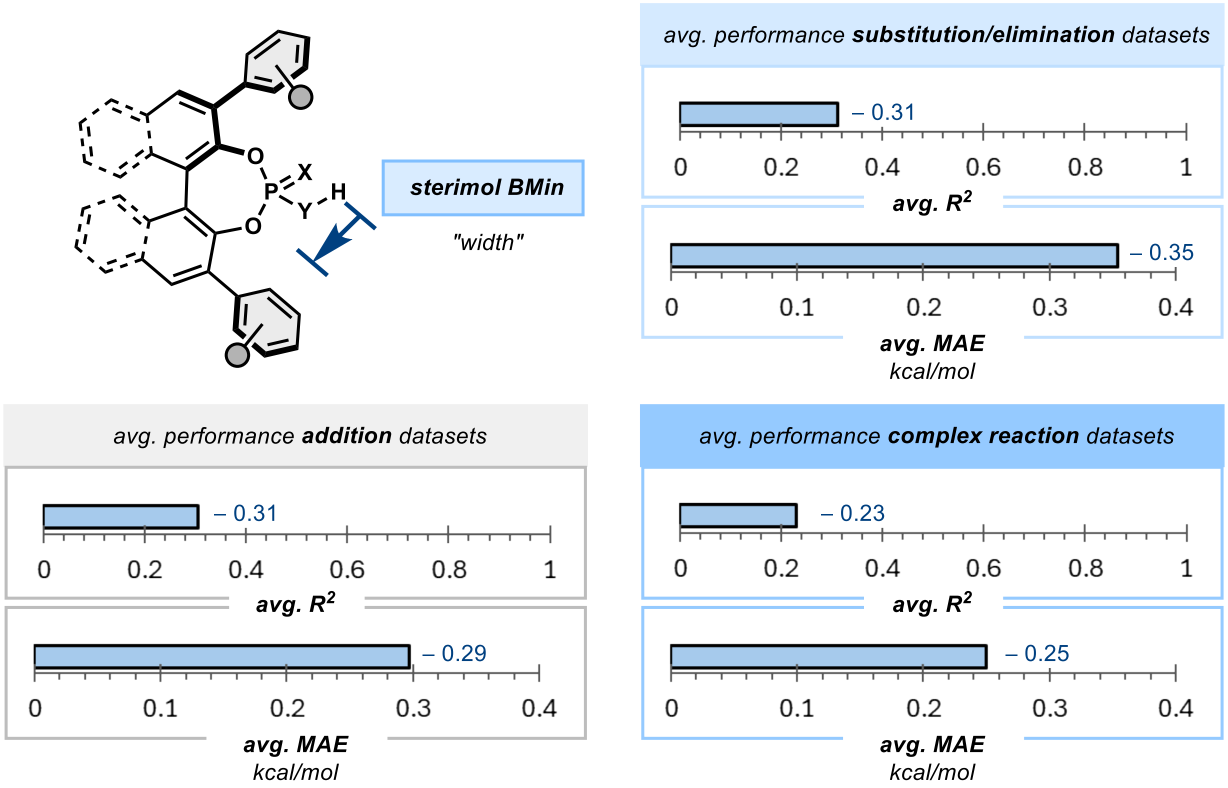

Supplementary Fig. 12. Average performance (R^2 and MAE in *kcal/mol*) across *acid-mediated addition* (bottom left), *acid-mediated substitutions and eliminations* (top right), and *complex/multicomponent reaction* (bottom right) datasets by *active site-based* sterimol BMin.

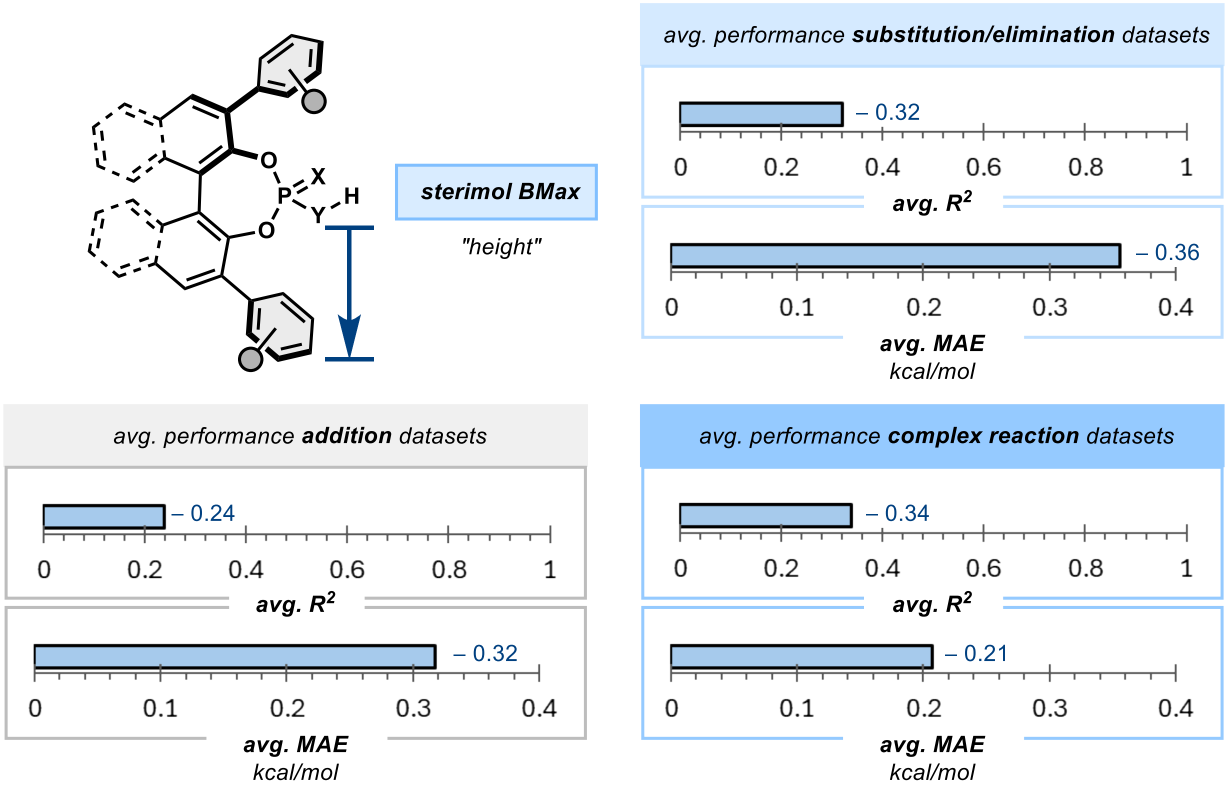

Supplementary Fig. 13. Average performance (R^2 and MAE in *kcal/mol*) across *acid-mediated addition* (bottom left), *acid-mediated substitutions and eliminations* (top right), and *complex/multicomponent reaction* (bottom right) datasets by *active site-based* sterimol BMax.

Regression Performance over Electronic-Controlled Datasets

In the main text, *substituent-based* ChelpG, *substituent-based* NBO charge, and *active site-based* buried volume were showcased in their performance across three datasets operating by electronic stereocontrol – R-**XI,** R-**XXII**, R-**XXVII**. We showcase the remaining electronic representations below. Of note, the three datasets are each *limited diversity*. As such, we show that buried volume (whether at the *substituent* or *active site*) are each a suitable representation to capture the underlying electronic effects.

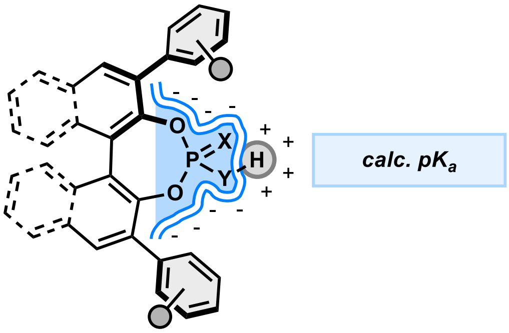

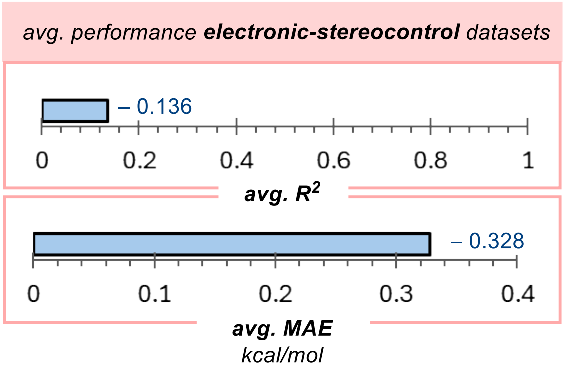

Supplementary Fig. 14. Average performance (R^2 and MAE in *kcal/mol*) across *electronic stereocontrolled* datasets (R-**XI**, R-**XXII**, and R-**XXVII**) by *calc. pK_a*.

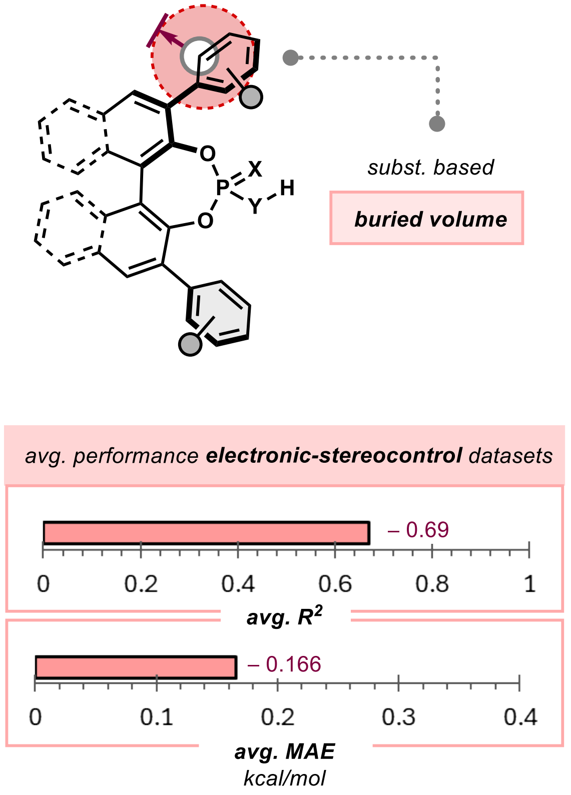

Supplementary Fig. 15. Average performance (R^2 and MAE in *kcal/mol*) across *electronic stereocontrolled* datasets (R-**XI**, R-**XXII**, and R-**XXVII**) by *substituent-based* buried volume.

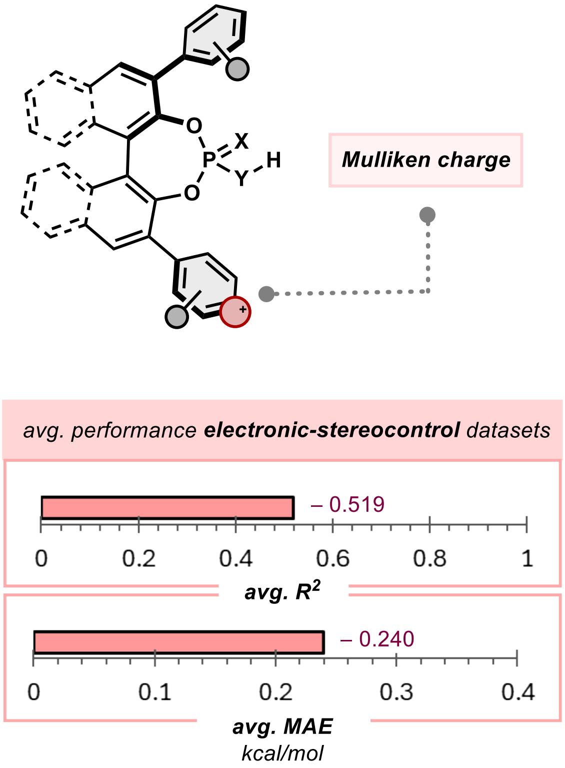

Supplementary Fig. 16. Average performance (R^2 and MAE in *kcal/mol*) across *electronic stereocontrolled* datasets (R-**XI**, R-**XXII**, and R-**XXVII**) by *substituent-based* Mulliken charge.

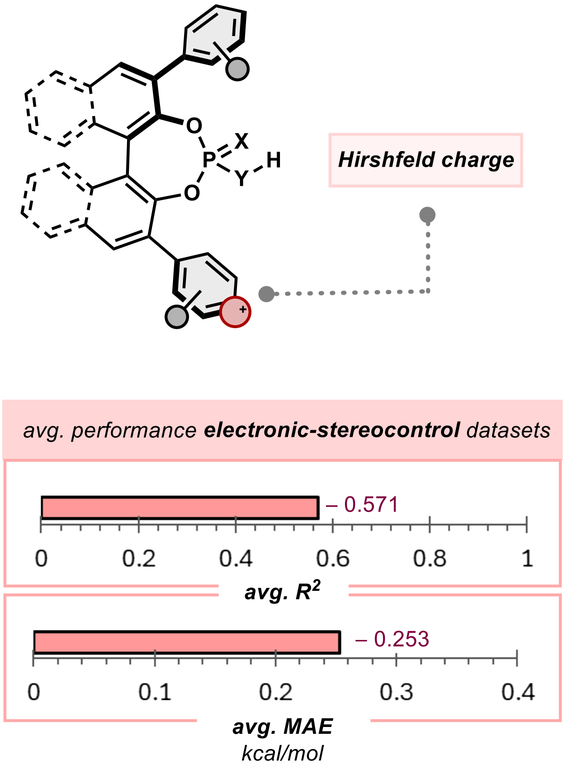

Supplementary Fig. 17. Average performance (R^2 and MAE in *kcal/mol*) across *electronic stereocontrolled* datasets (R-**XI**, R-**XXII**, and R-**XXVII**) by *substituent-based* Hirshfeld.

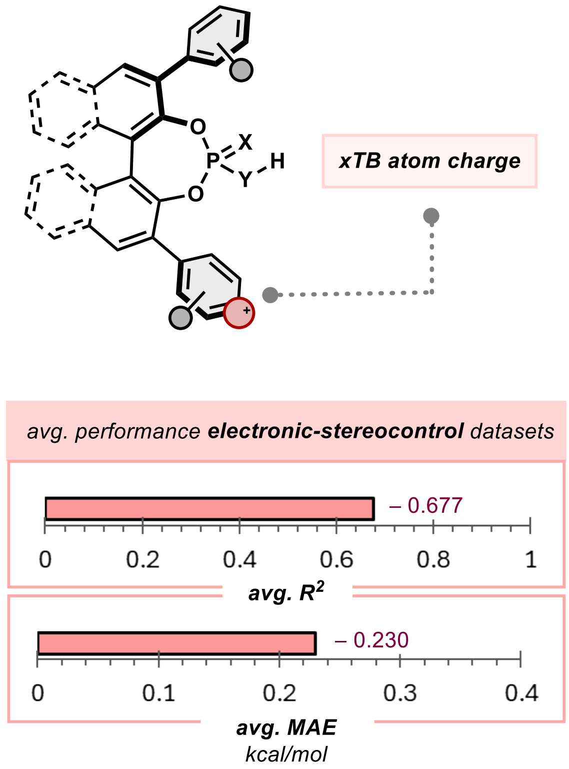

Supplementary Fig. 18. Average performance (R^2 and MAE in *kcal/mol*) across *electronic stereocontrolled* datasets (R-**XI**, R-**XXII**, and R-**XXVII**) by *substituent-based* xTB atom charge.

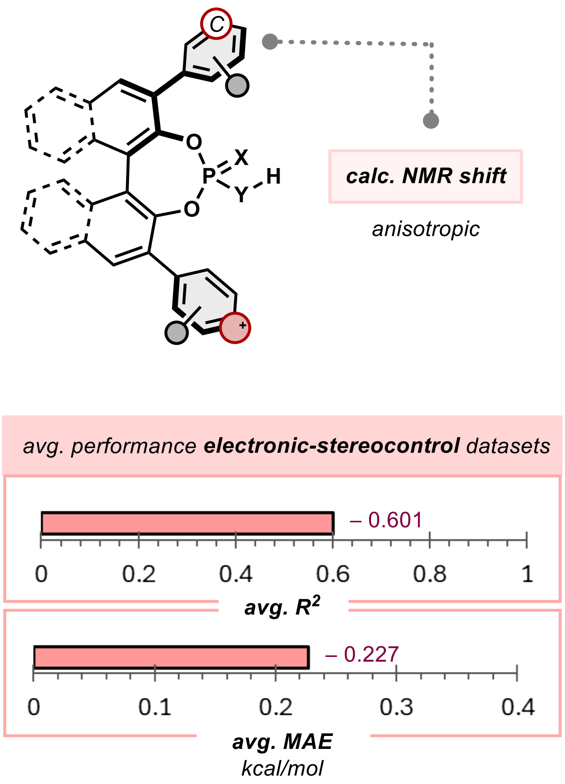

Supplementary Fig. 19. Average performance (R^2 and MAE in *kcal/mol*) across *electronic stereocontrolled* datasets (R-**XI**, R-**XXII**, and R-**XXVII**) by *substituent-based* calculated anisotropic NMR shift.

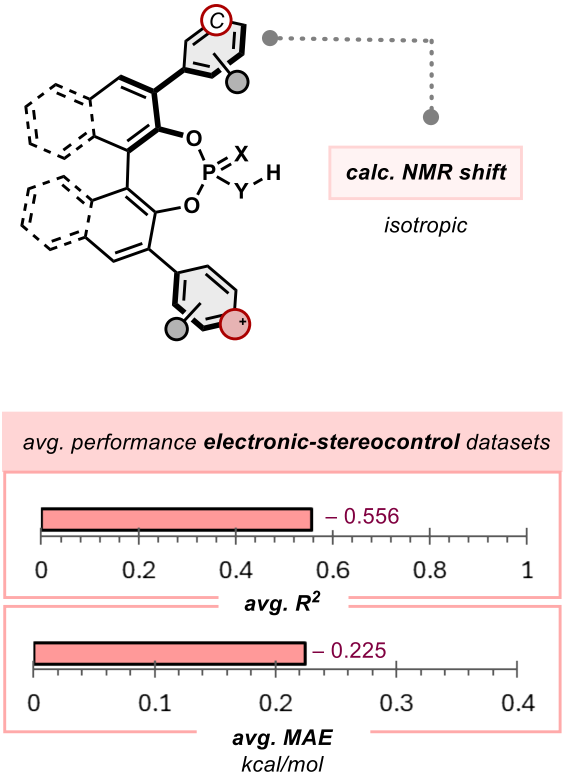

Supplementary Fig. 20. Average performance (R^2 and MAE in *kcal/mol*) across *electronic stereocontrolled* datasets (R-**XI**, R-**XXII**, and R-**XXVII**) by *substituent-based* calculated isotropic NMR shift

Univariate Regression Models from Active Site-based Buried Volume

The following correlations between the selected datasets and *active site-based* buried volume are invoked through the main text and the SI. For clarity (in transformation, dataset, and correlation), we show all 54 correlations below to *active site-based* buried volume. The source for each dataset is tabulated in *dataset_rxn.xlsx* for reference under the corresponding key.

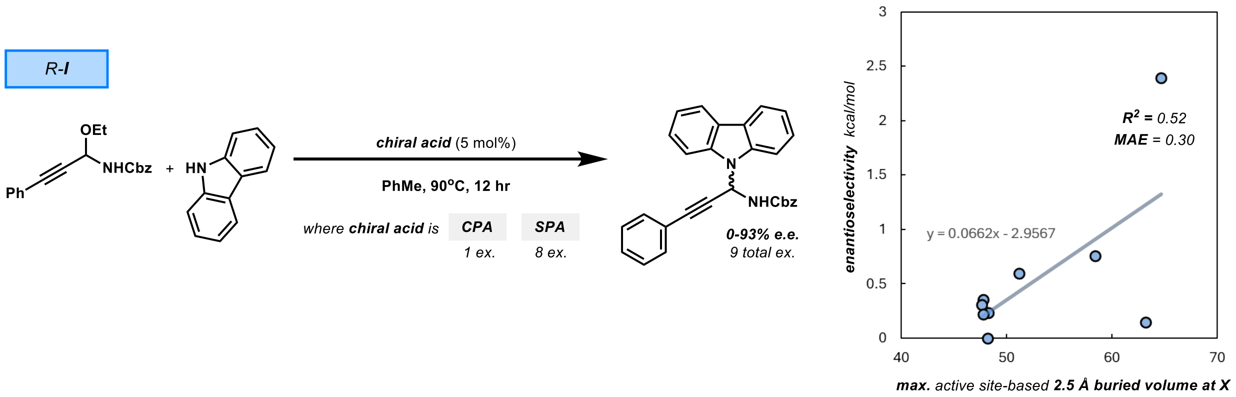

Supplementary Fig. 21. R-**I** correlation drawn by *active site-based* buried volume.

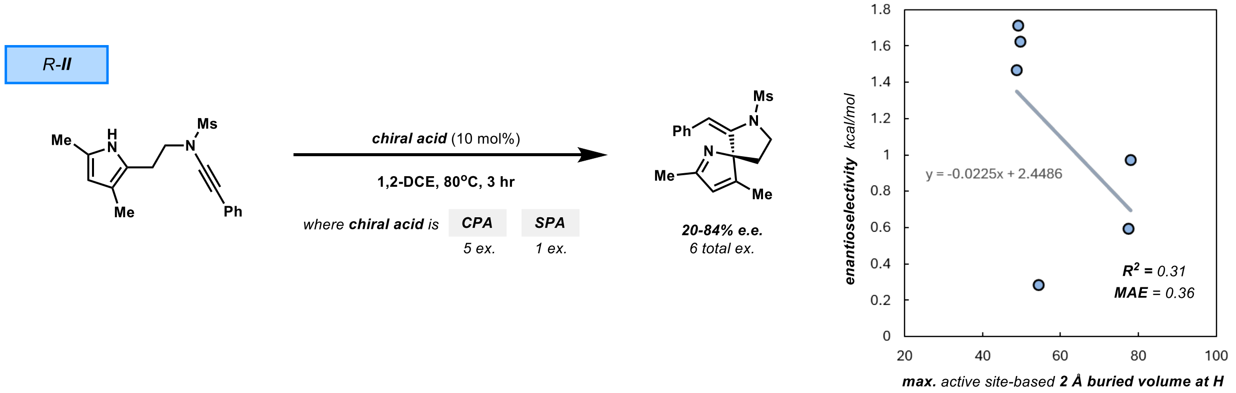

Supplementary Fig. 22. R-**II** correlation drawn by *active site-based* buried volume.

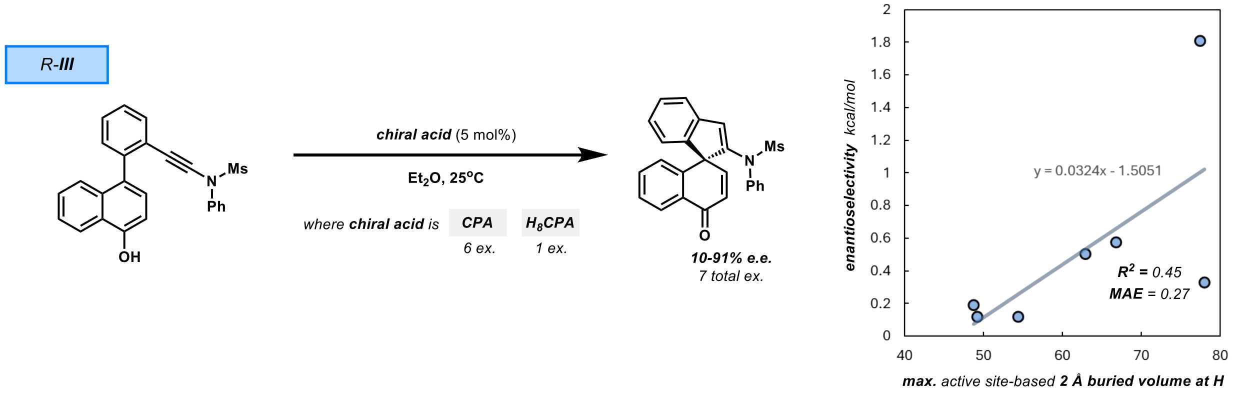

Supplementary Fig. 23. R-**III** correlation drawn by *active site-based* buried volume.

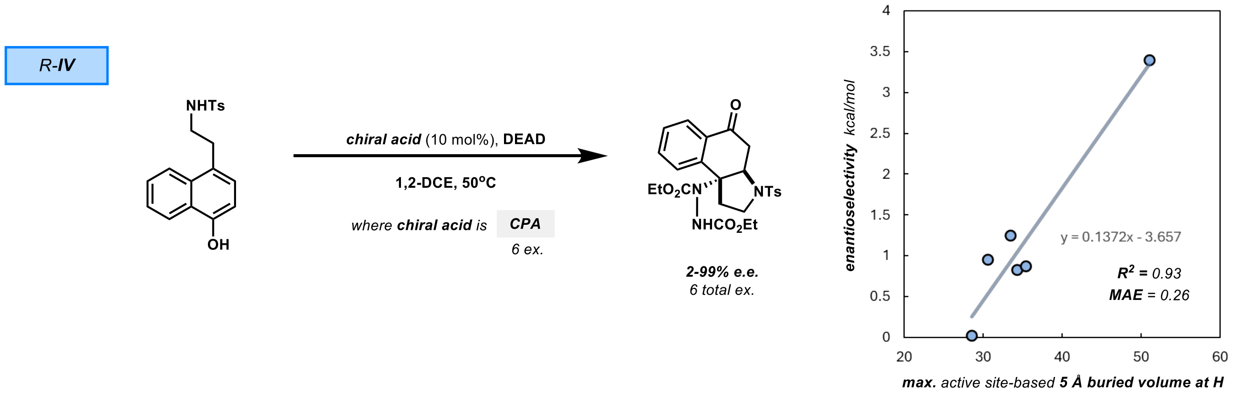

Supplementary Fig. 24. R-**IV** correlation drawn by *active site-based* buried volume.

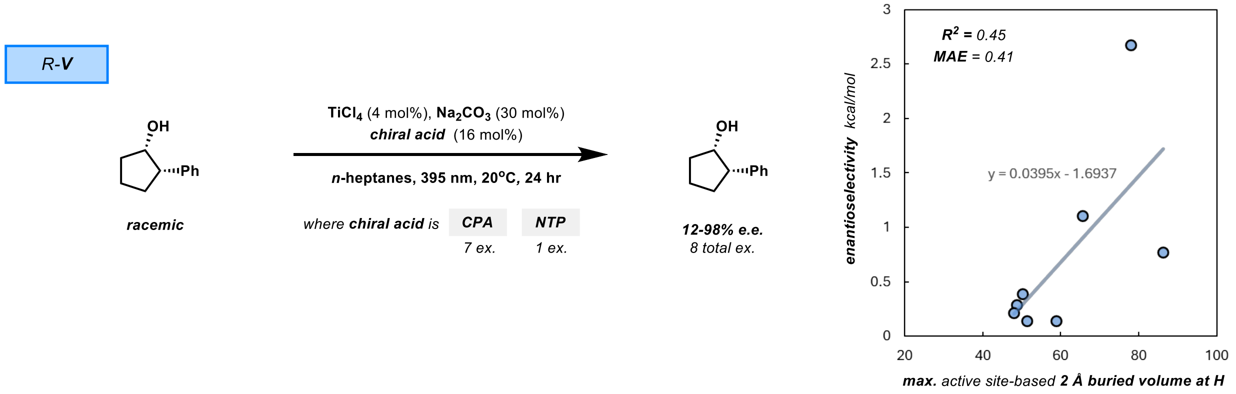

Supplementary Fig. 25. R-**V** correlation drawn by *active site-based* buried volume.

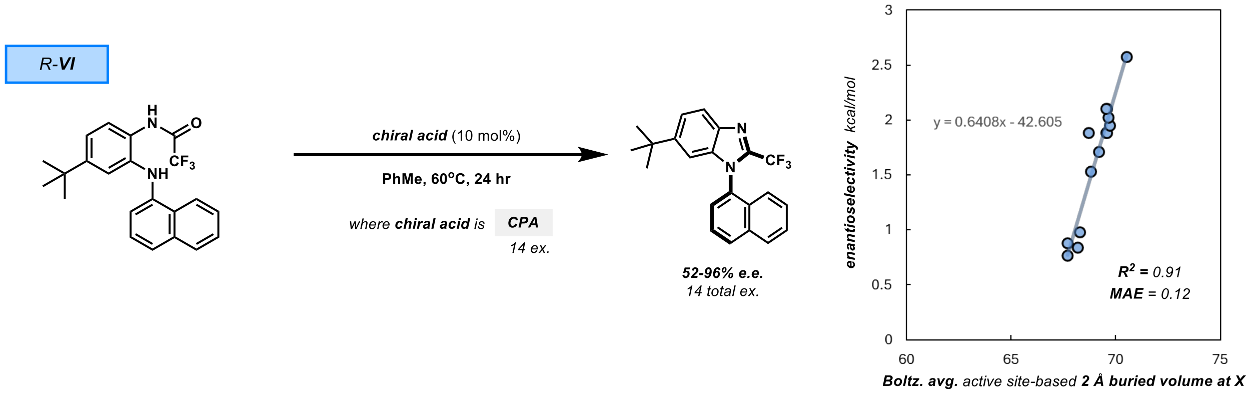

Supplementary Fig. 26. R-**VI** correlation drawn by *active site-based* buried volume.

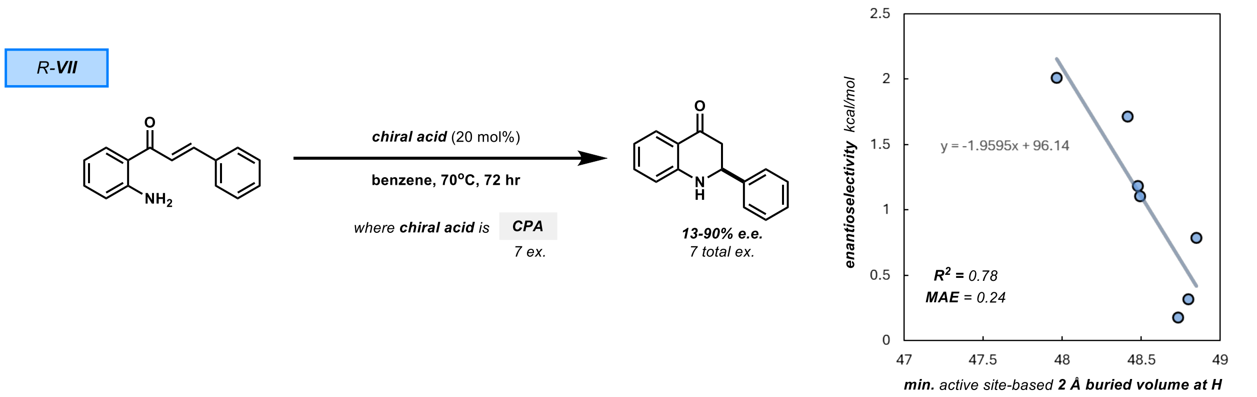

Supplementary Fig. 27. R-**VII** correlation drawn by *active site-based* buried volume.

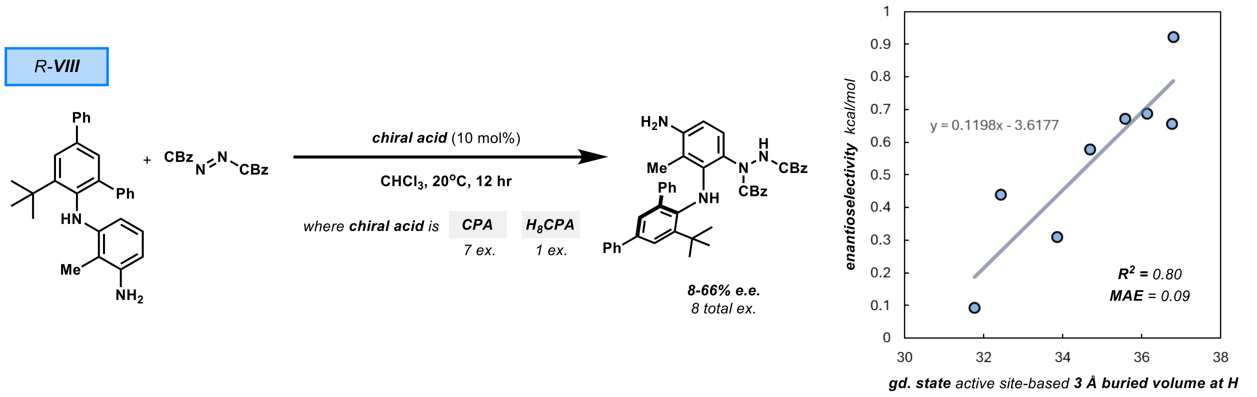

Supplementary Fig. 28. R-**VIII** correlation drawn by *active site-based* buried volume.

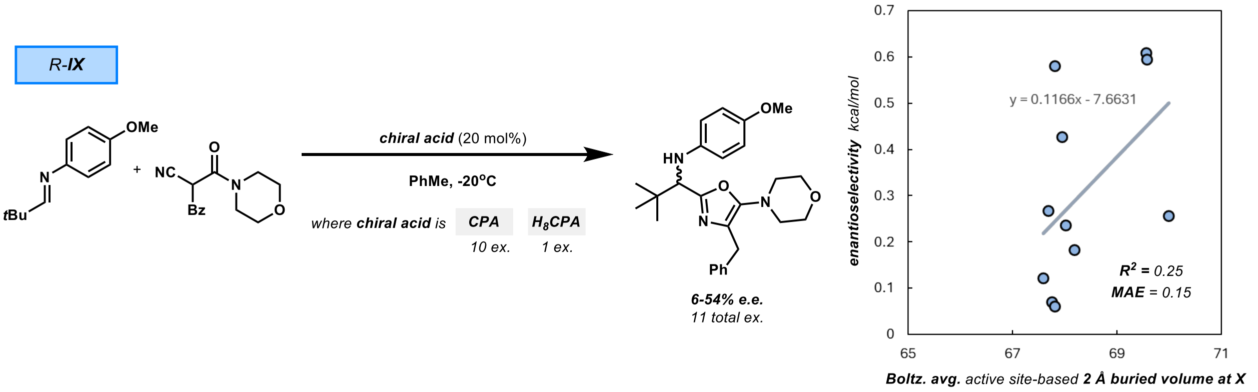

Supplementary Fig. 29. R-**IX** correlation drawn by *active site-based* buried volume.

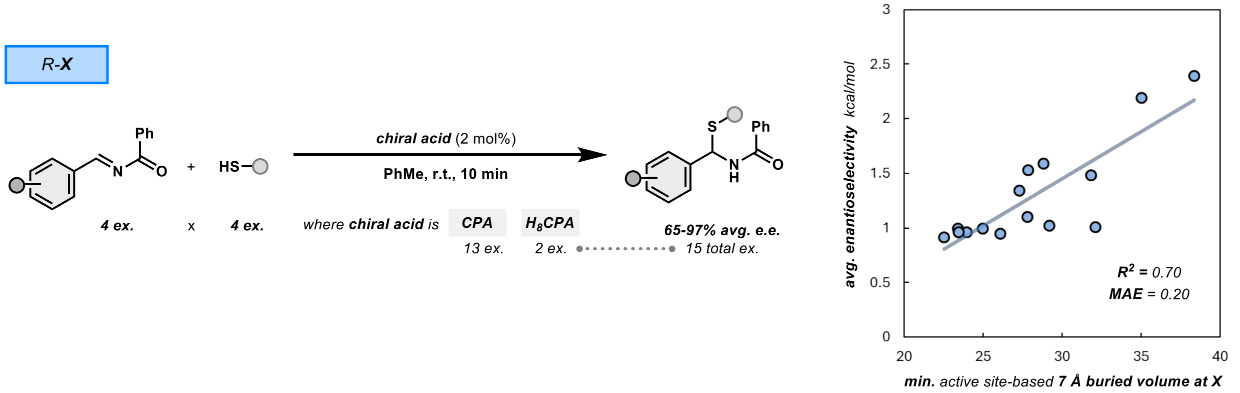

Supplementary Fig. 30. R-**X** correlation drawn by *active site-based* buried volume.

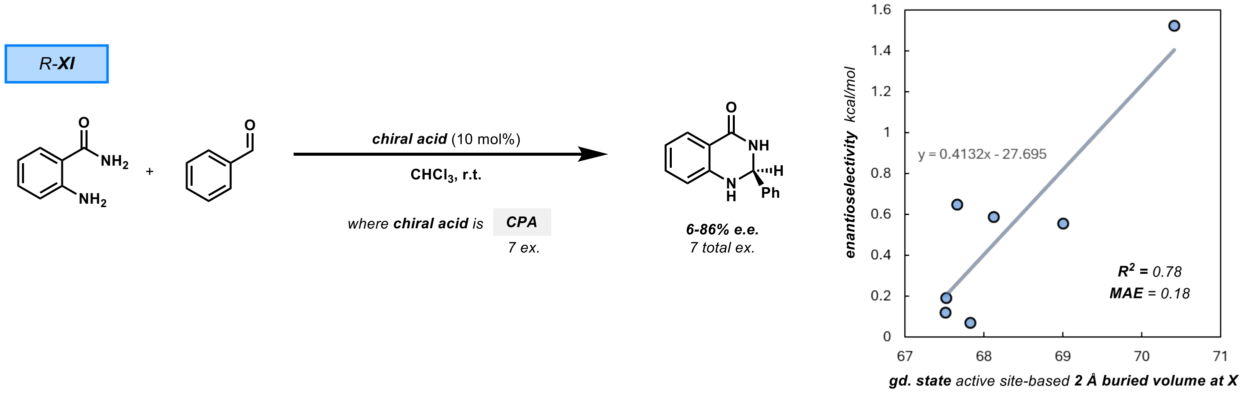

Supplementary Fig. 31. R-**XI** correlation drawn by *active site-based* buried volume.

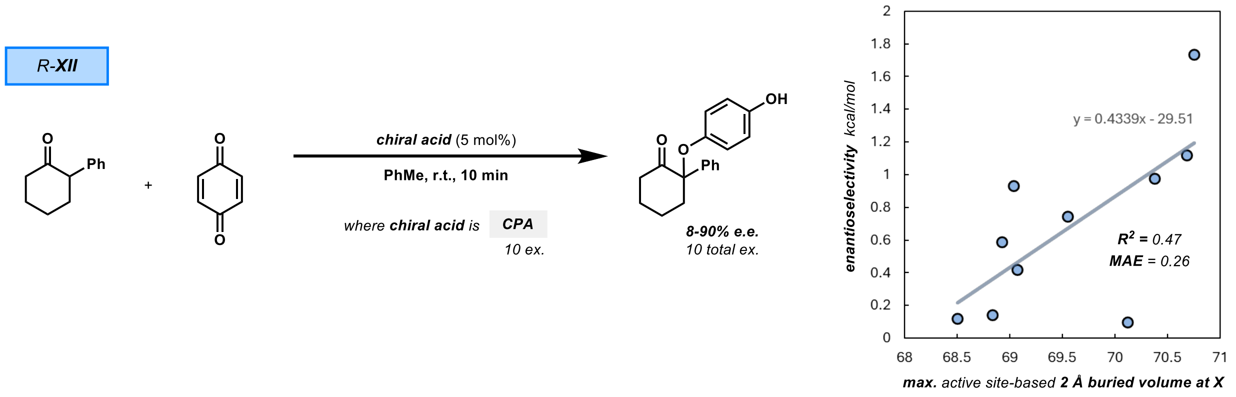

Supplementary Fig. 32. R-**XII** correlation drawn by *active site-based* buried volume.

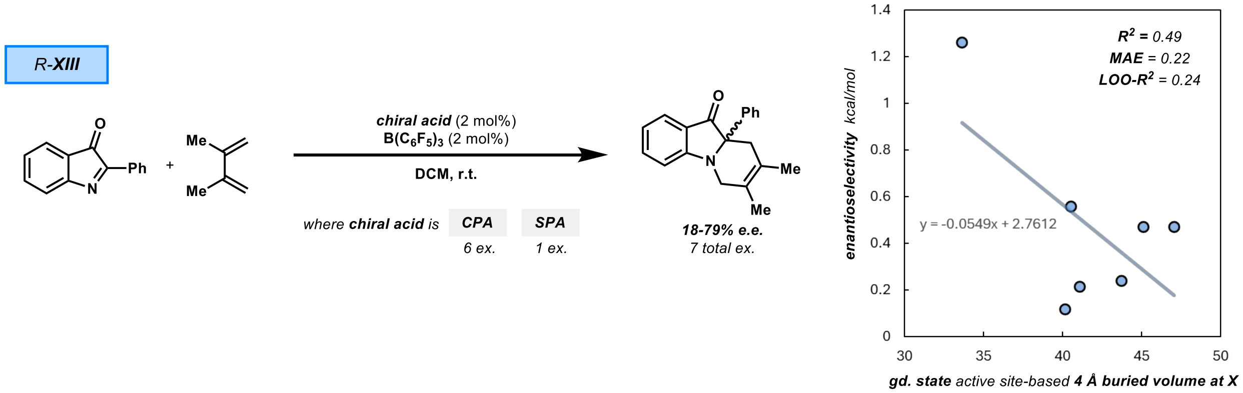

Supplementary Fig. 33. R-**XIII** correlation drawn by *active site-based* buried volume.

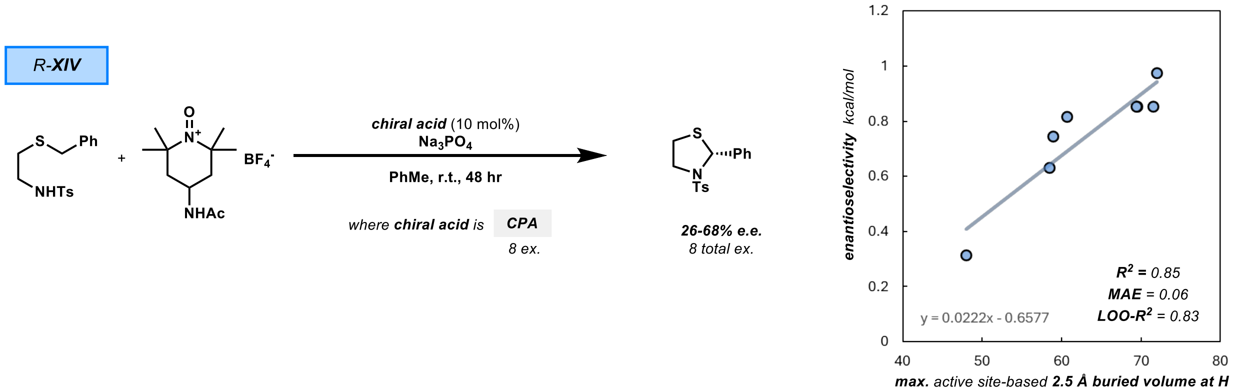

Supplementary Fig. 34. R-**XIV** correlation drawn by *active site-based* buried volume.

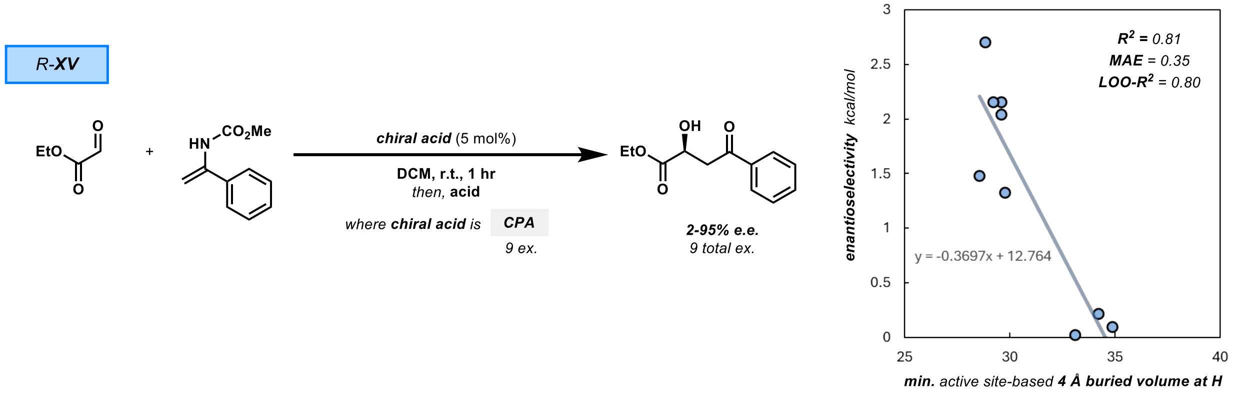

Supplementary Fig. 35. R-**XV** correlation drawn by *active site-based* buried volume.

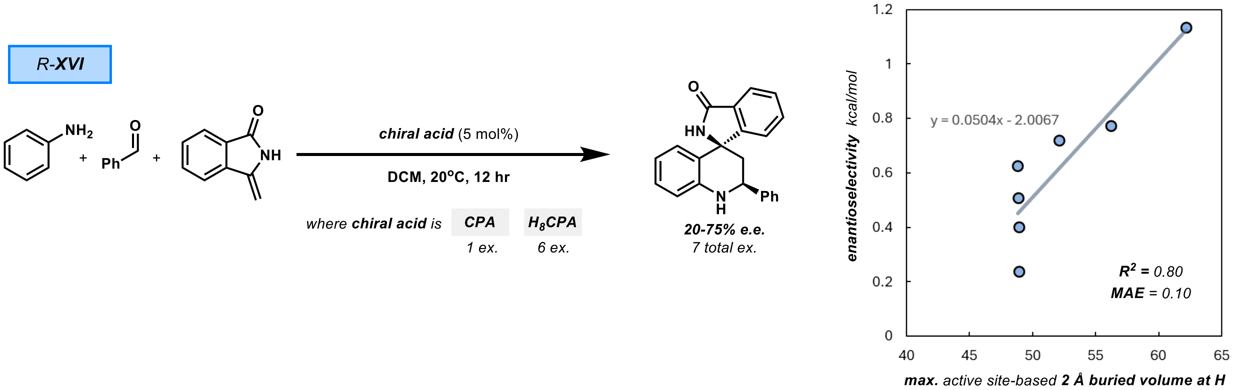

Supplementary Fig. 36. R-**XVI** correlation drawn by *active site-based* buried volume.

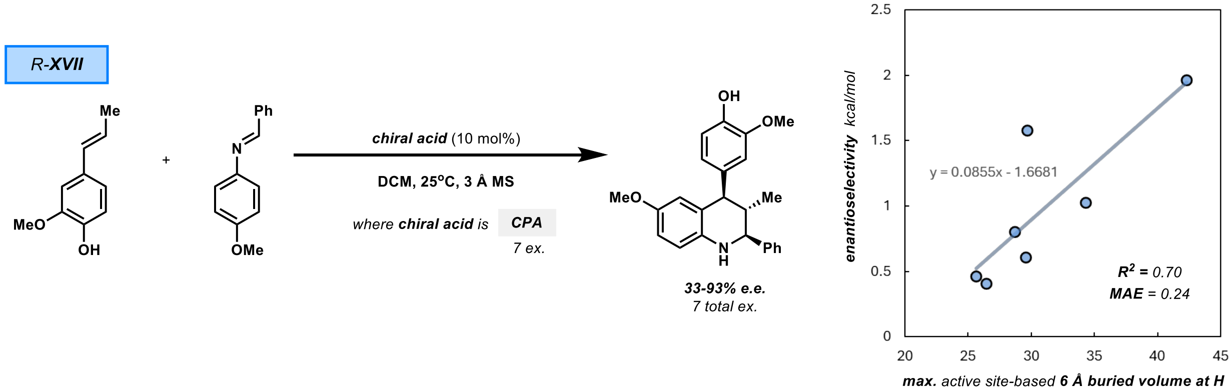

Supplementary Fig. 37. R-**XVII** correlation drawn by *active site-based* buried volume.

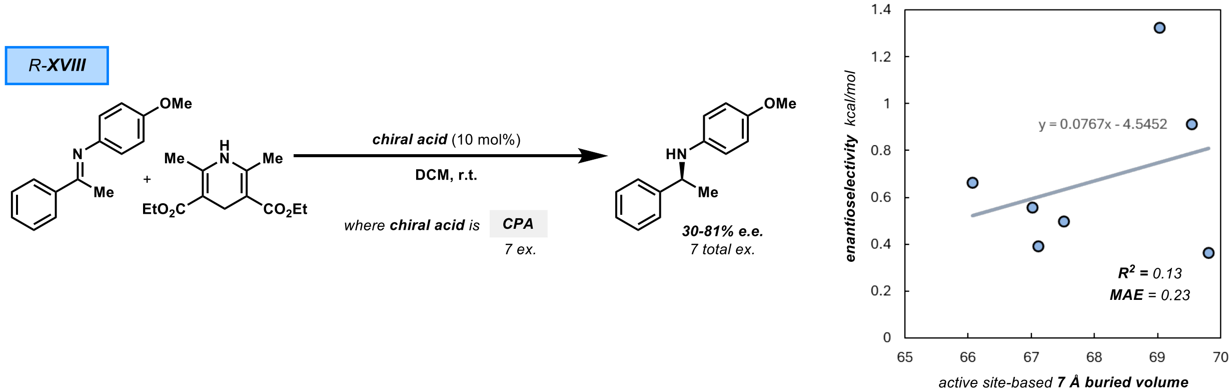

Supplementary Fig. 38. R-**XVIII** correlation drawn by *active site-based* buried volume.

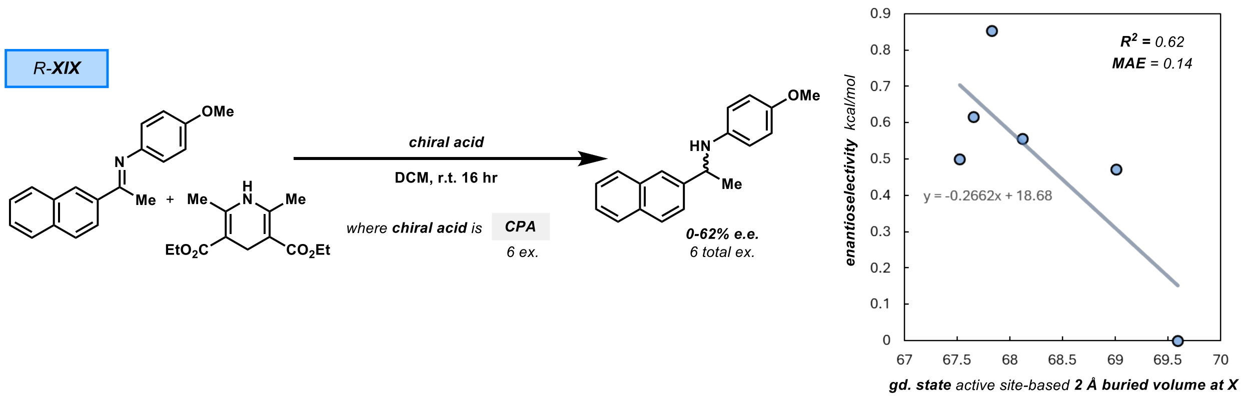

Supplementary Fig. 39. R-**XIX** correlation drawn by *active site-based* buried volume.

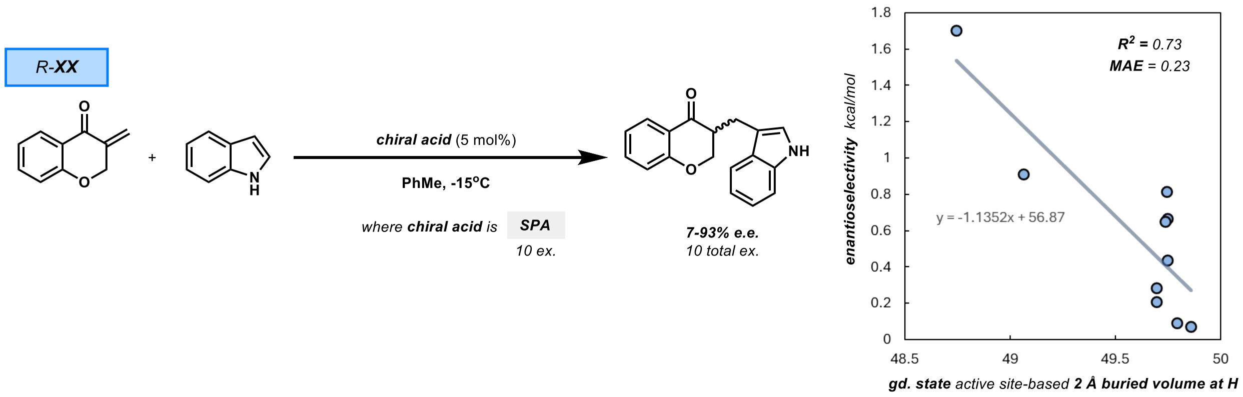

Supplementary Fig. 40. R-**XX** correlation drawn by *active site-based* buried volume.

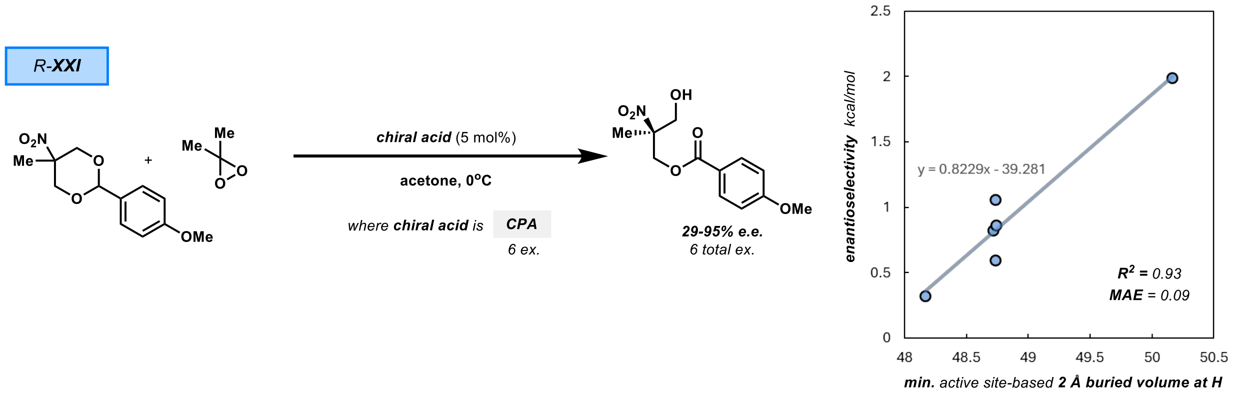

Supplementary Fig. 41. R-**XXI** correlation drawn by *active site-based* buried volume.

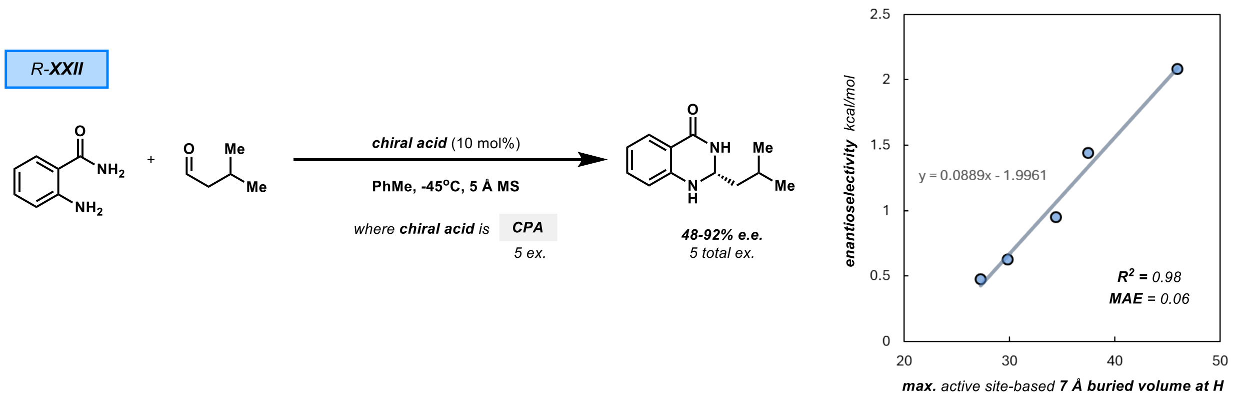

Supplementary Fig. 42. R-**XXII** correlation drawn by *active site-based* buried volume.

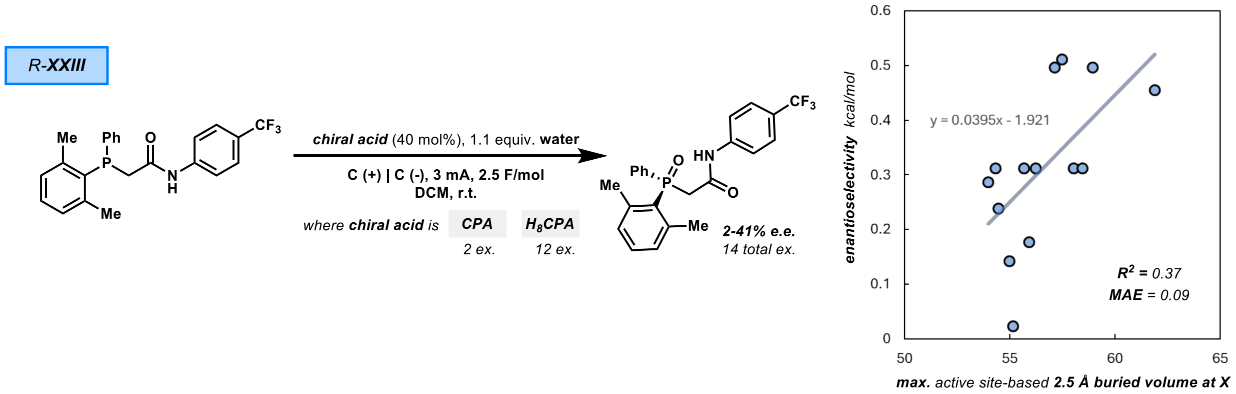

Supplementary Fig. 43. R-**XXIII** correlation drawn by *active site-based* buried volume.

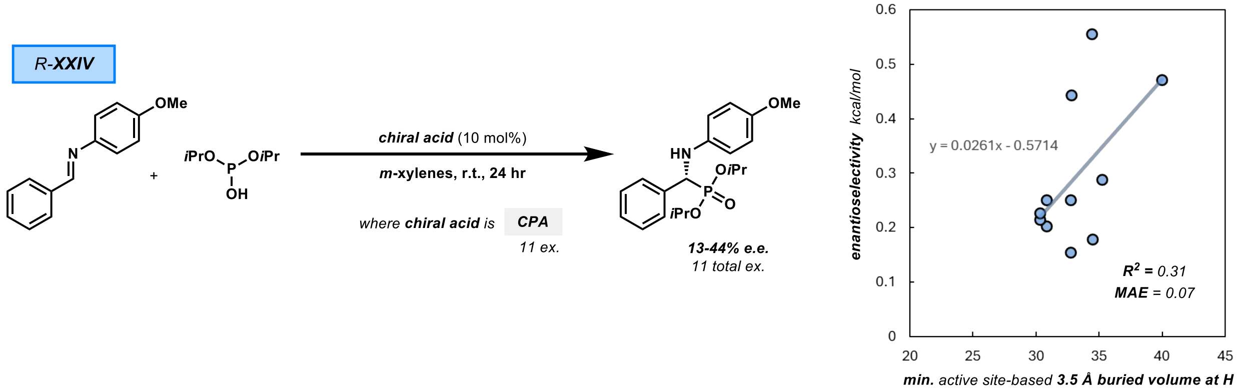

Supplementary Fig. 44. R-**XXIV** correlation drawn by *active site-based* buried volume.

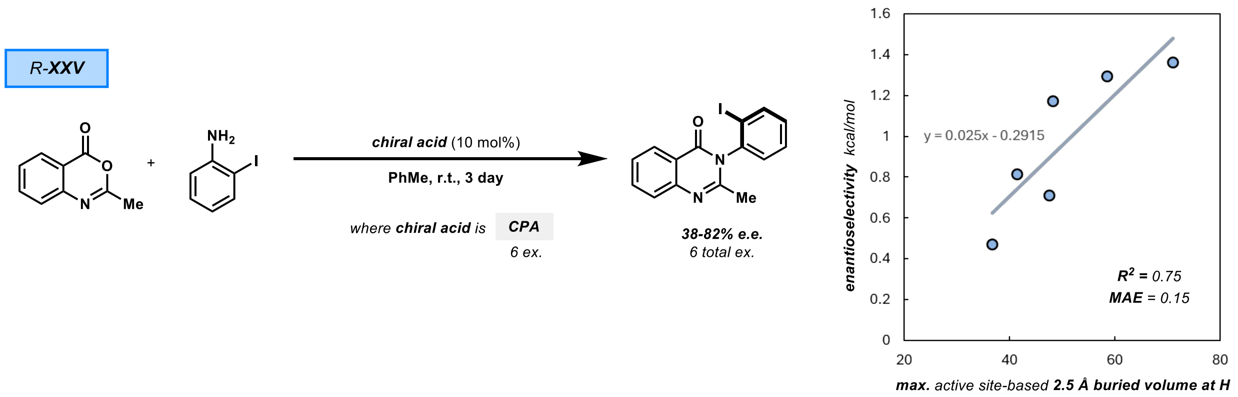

Supplementary Fig. 45. R-**XXV** correlation drawn by *active site-based* buried volume.

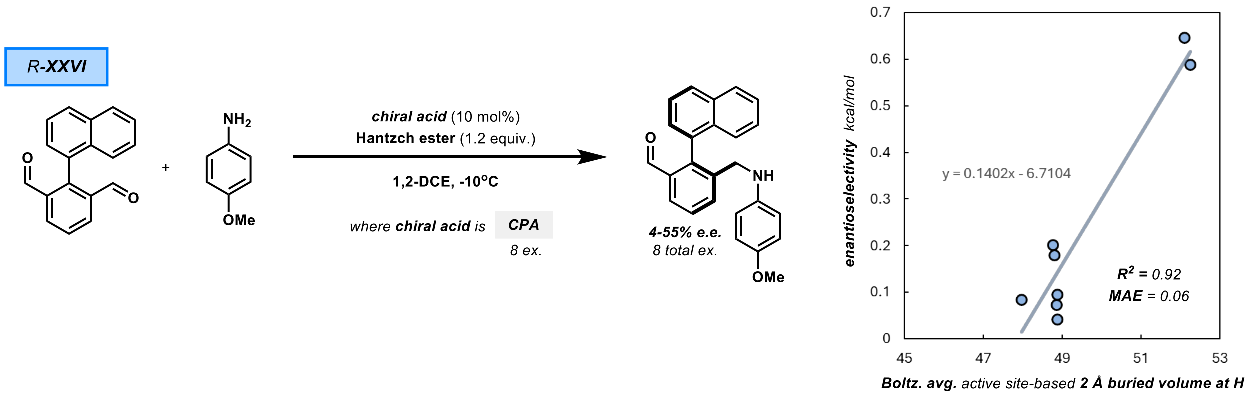

Supplementary Fig. 46. R-**XXVI** correlation drawn by *active site-based* buried volume.

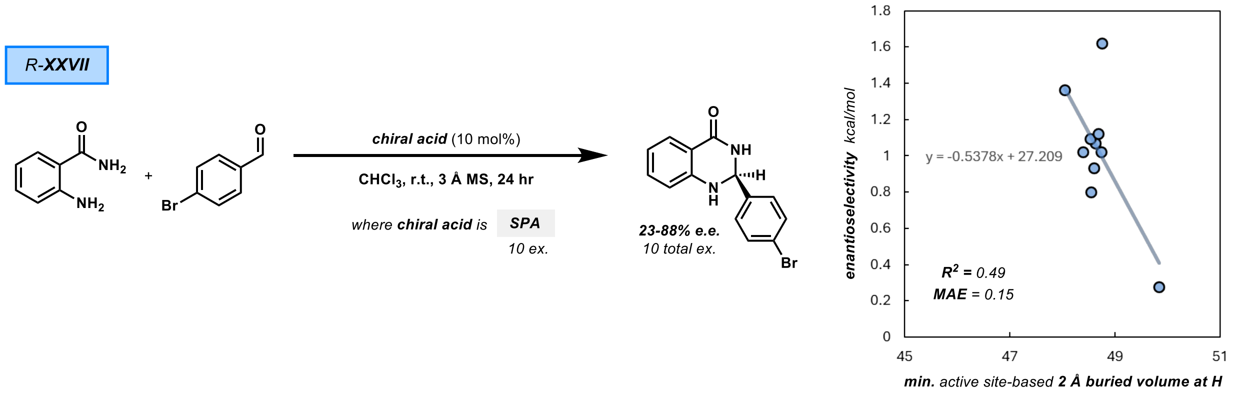

Supplementary Fig. 47. R-**XXVII** correlation drawn by *active site-based* buried volume.

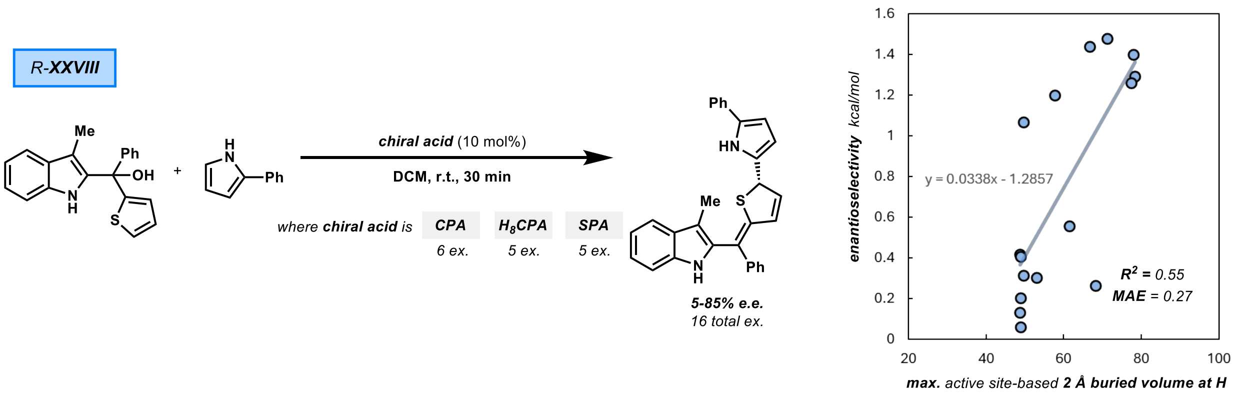

Supplementary Fig. 48. R-**XXVIII** correlation drawn by *active site-based* buried volume.

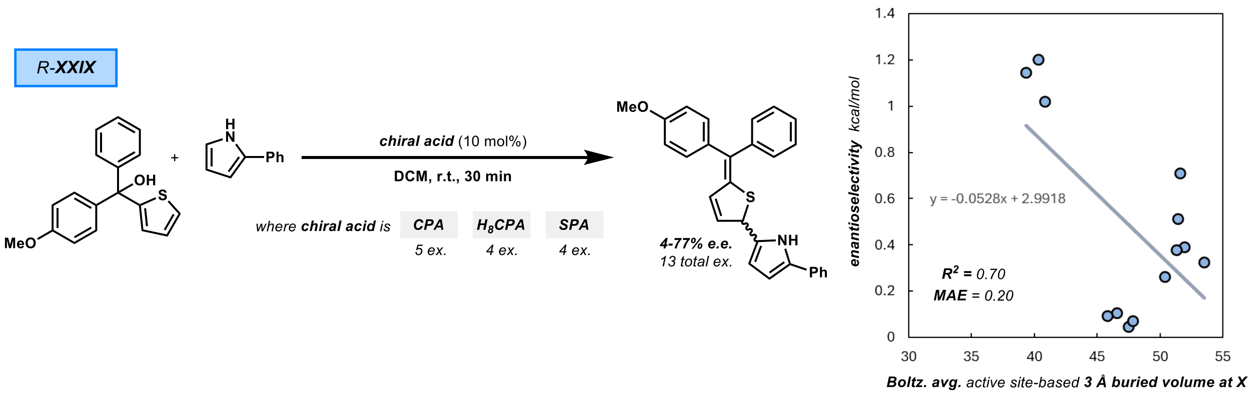

Supplementary Fig. 49. R-**XXIX** correlation drawn by *active site-based* buried volume.

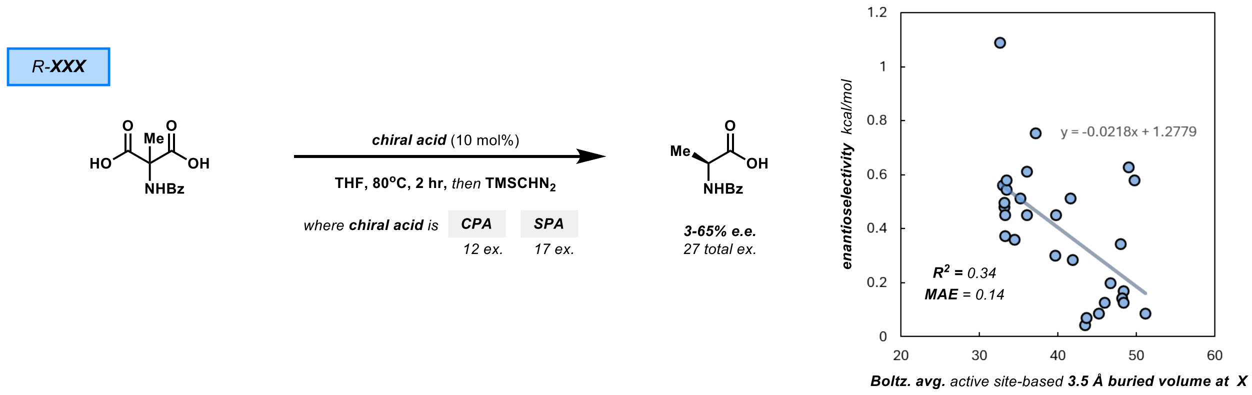

Supplementary Fig. 50. R-**XXX** correlation drawn by *active site-based* buried volume.

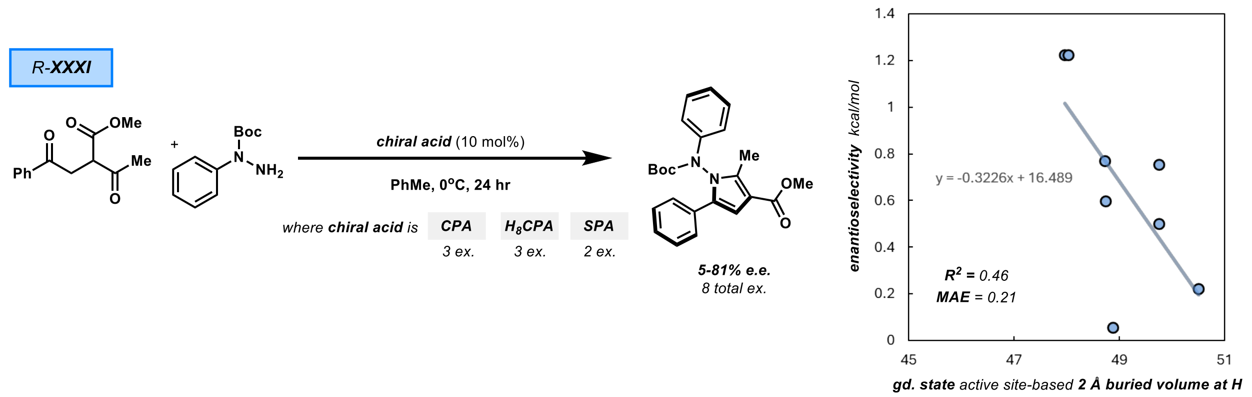

Supplementary Fig. 51. R-**XXXI** correlation drawn by *active site-based* buried volume.

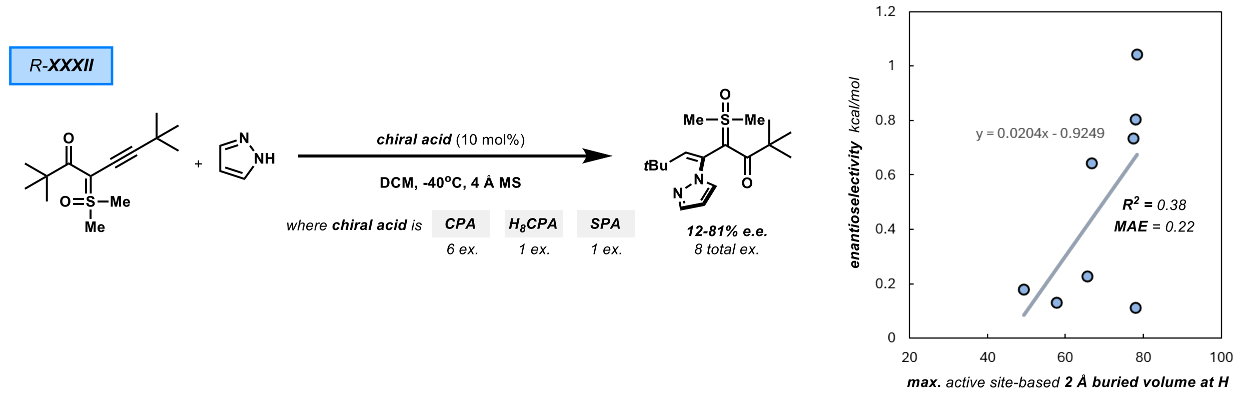

Supplementary Fig. 52. R-**XXXII** correlation drawn by *active site-based* buried volume.

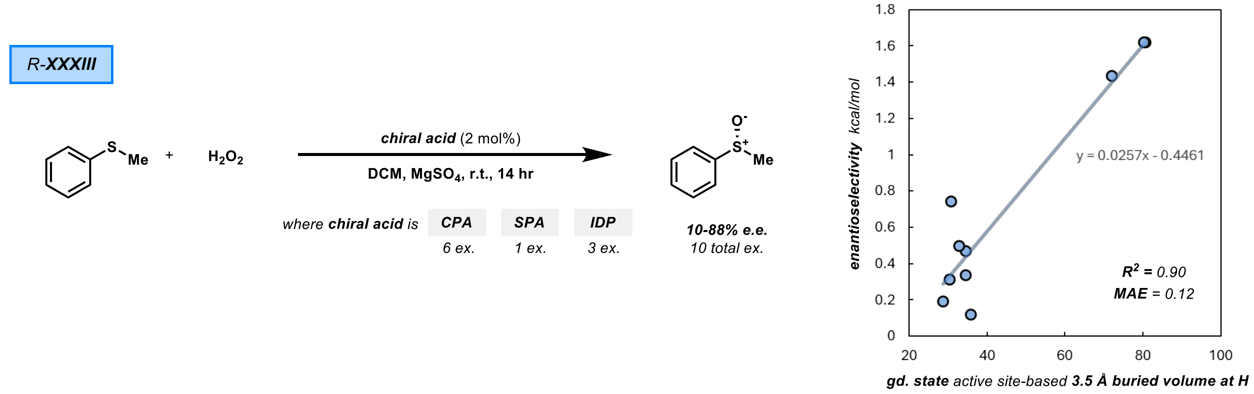

Supplementary Fig. 53. R-**XXXIII** correlation drawn by *active site-based* buried volume.

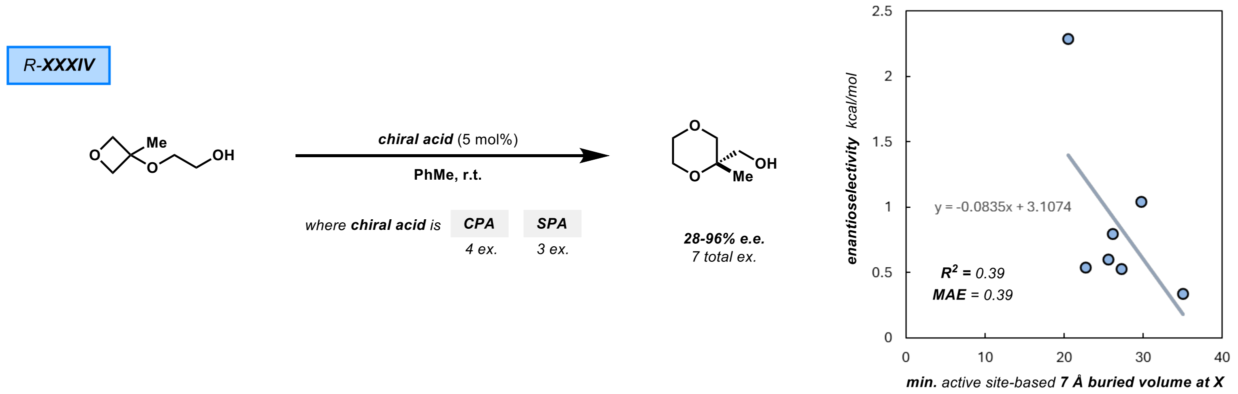

Supplementary Fig. 54. R-**XXXIV** correlation drawn by *active site-based* buried volume.

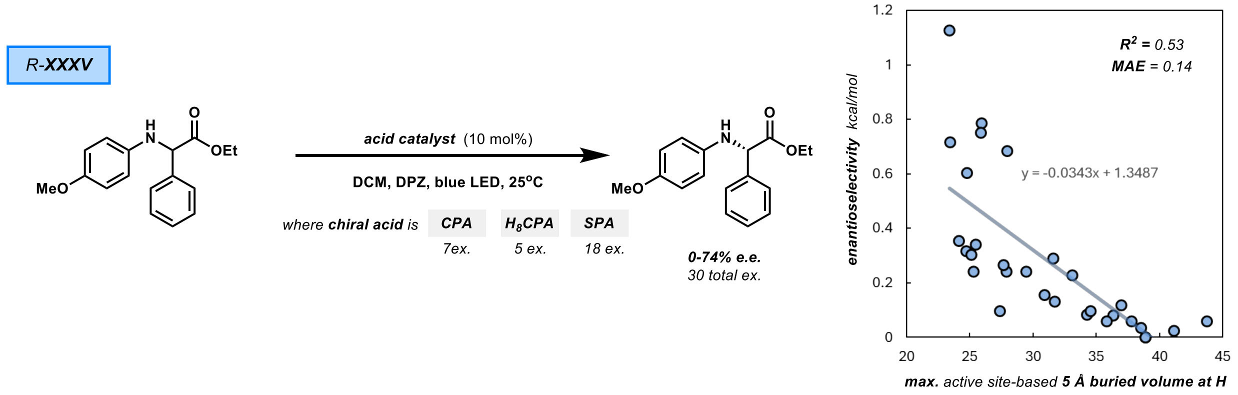

Supplementary Fig. 55. R-**XXXV** correlation drawn by *active site-based* buried volume.

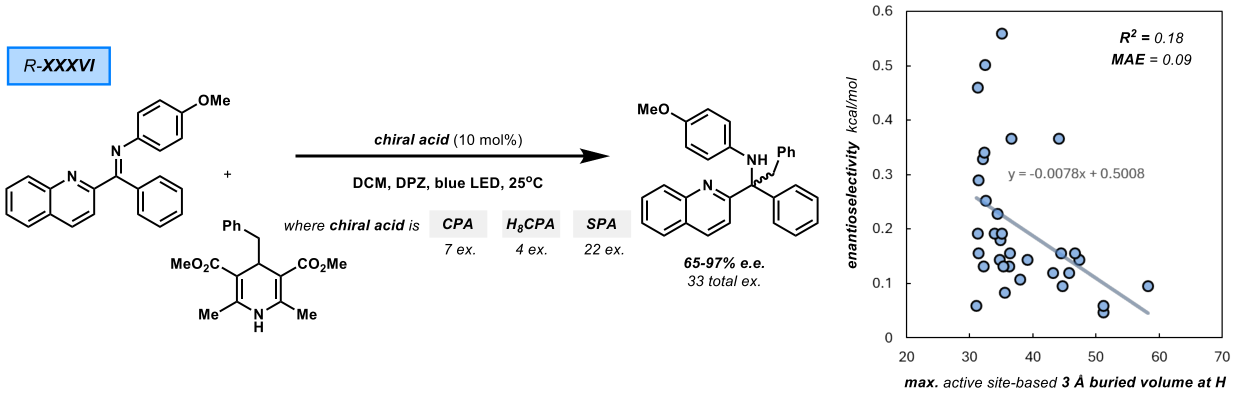

Supplementary Fig. 56. R-**XXXVI** correlation drawn by *active site-based* buried volume.

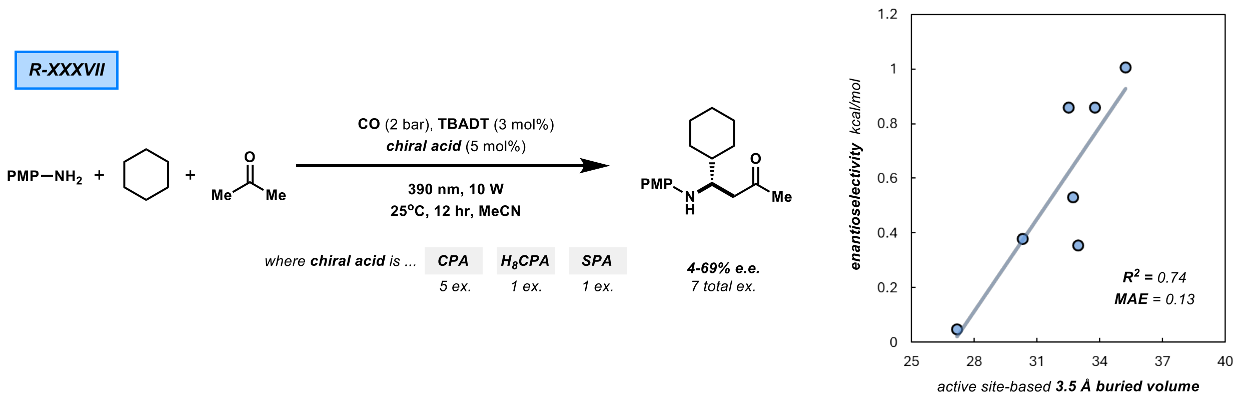

Supplementary Fig. 57. R-**XXXVII** correlation drawn by *active site-based* buried volume.

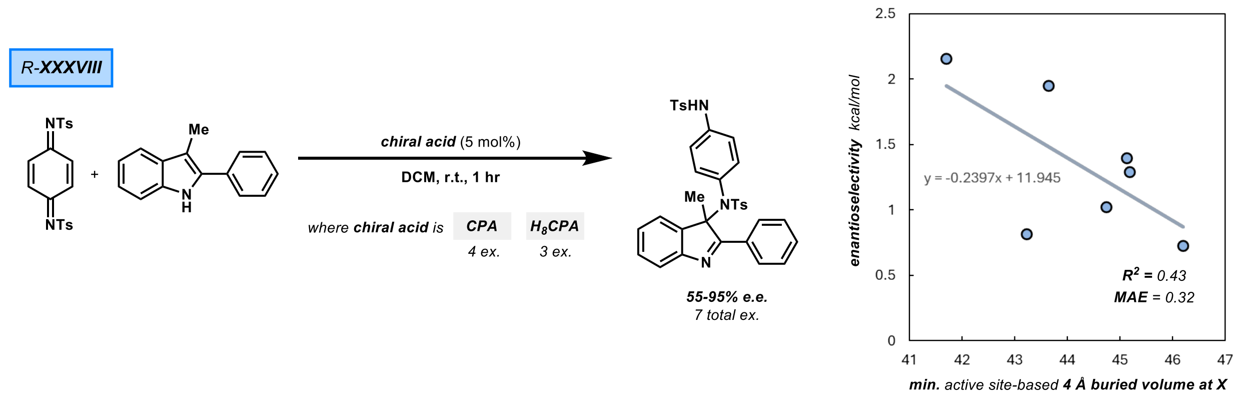

Supplementary Fig. 58. R-**XXXVIII** correlation drawn by *active site-based* buried volume.

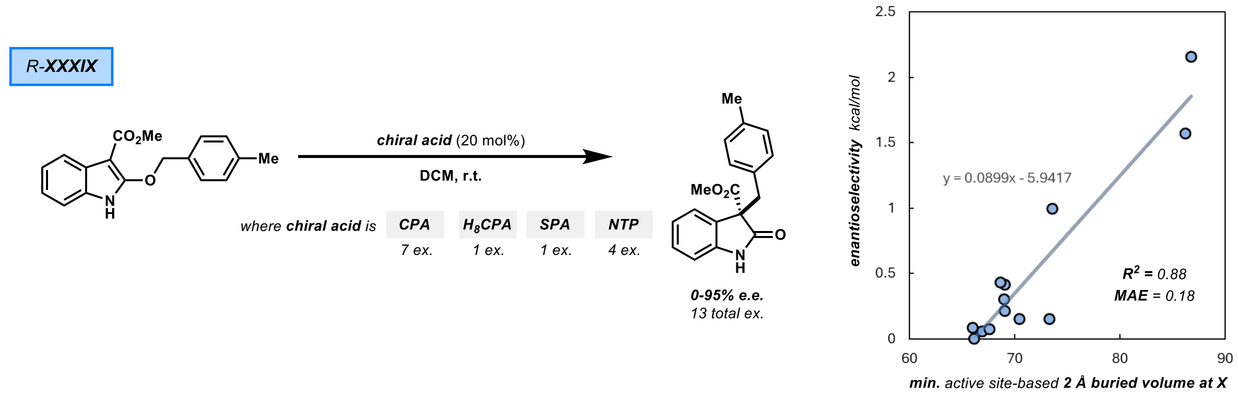

Supplementary Fig. 59. R-**XXXIX** correlation drawn by *active site-based* buried volume.

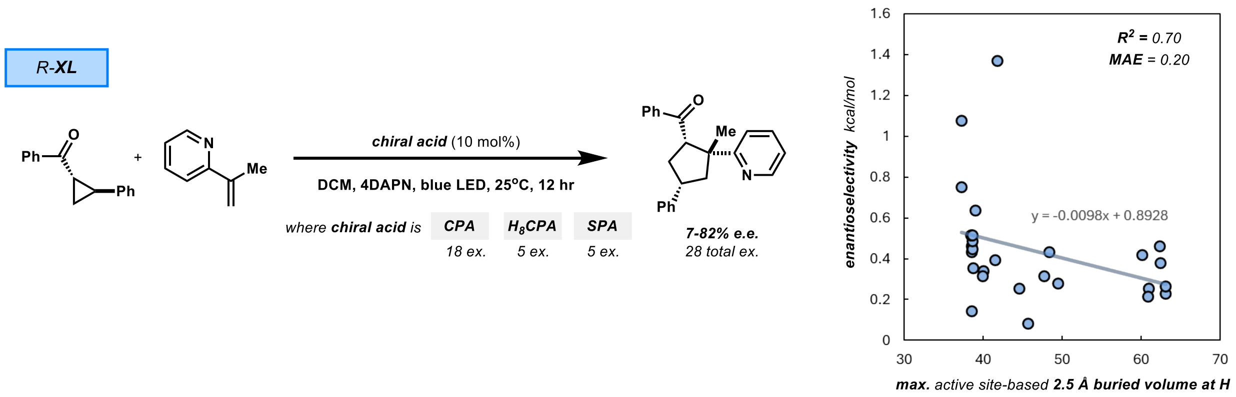

Supplementary Fig. 60. R-**XL** correlation drawn by *active site-based* buried volume.

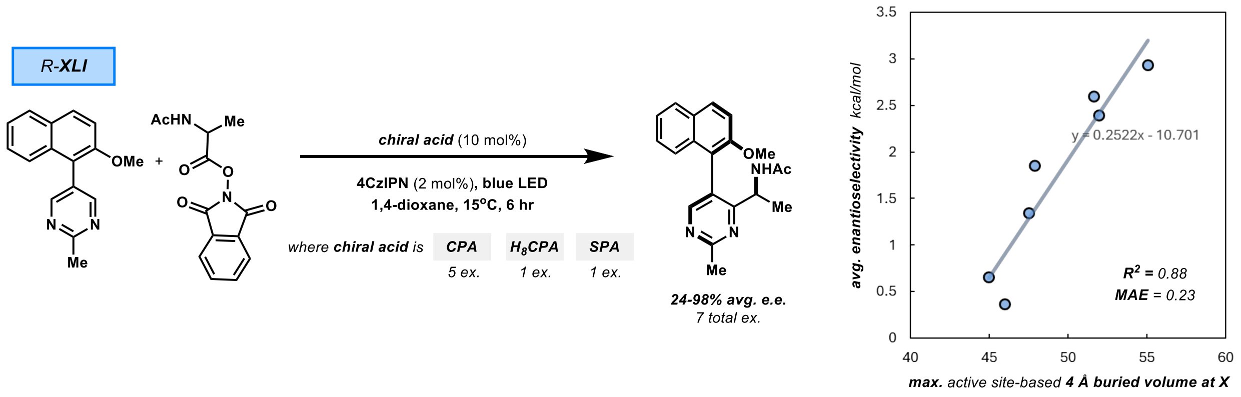

Supplementary Fig. 61. R-**XLI** correlation drawn by *active site-based* buried volume.

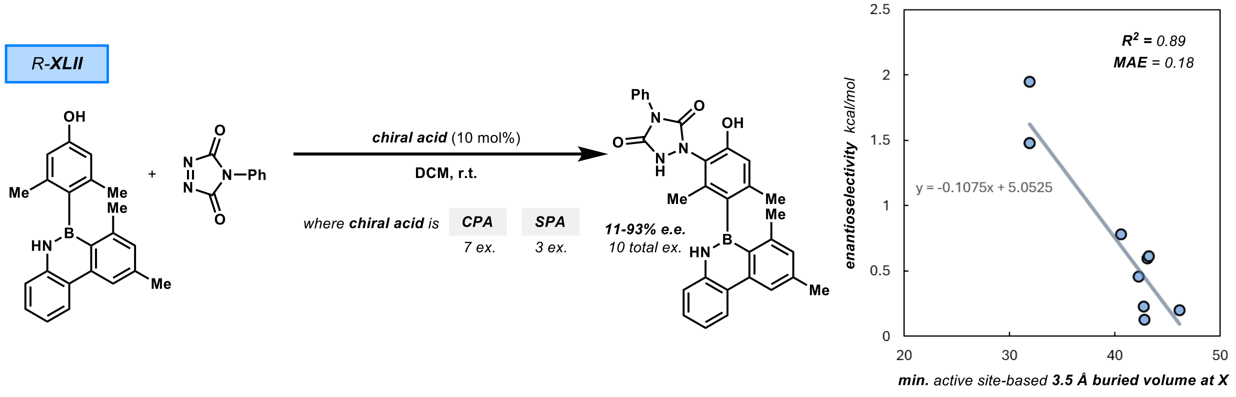

Supplementary Fig. 62. R-**XLII** correlation drawn by *active site-based* buried volume.

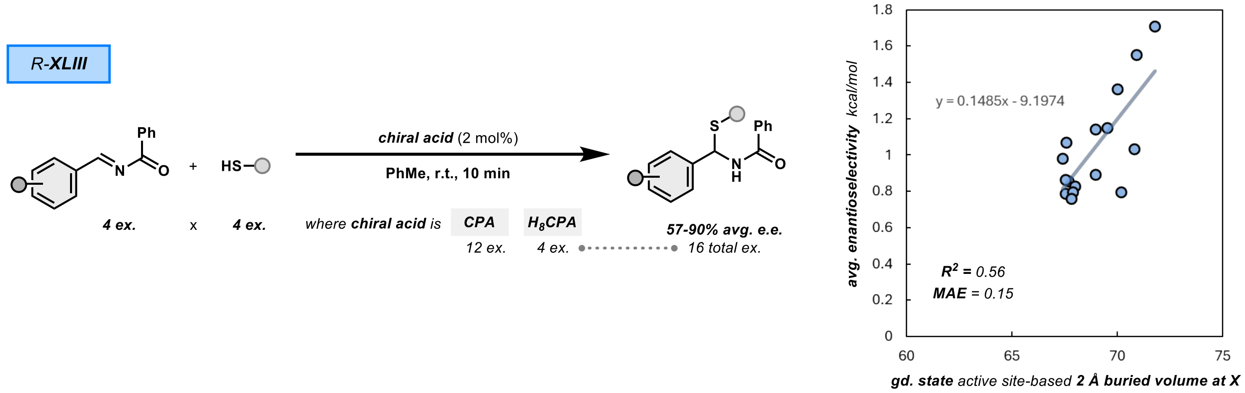

Supplementary Fig. 63. R-**XLIII** correlation drawn by *active site-based* buried volume.

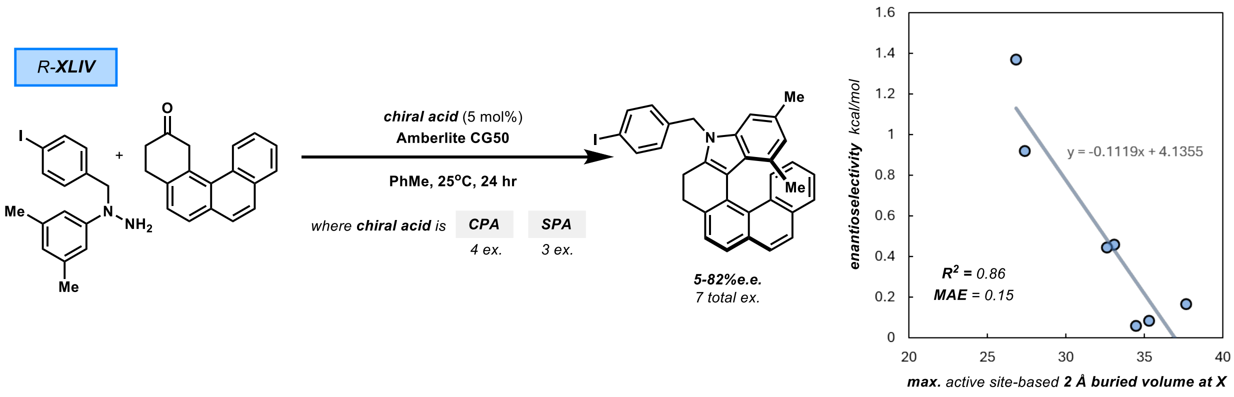

Supplementary Fig. 64. R-**XLIV** correlation drawn by *active site-based* buried volume.

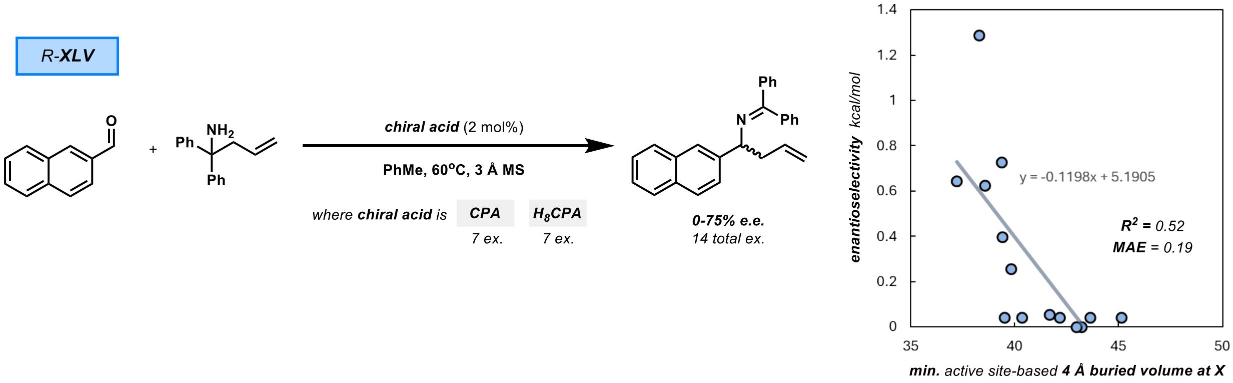

Supplementary Fig. 65. R-**XLV** correlation drawn by *active site-based* buried volume.

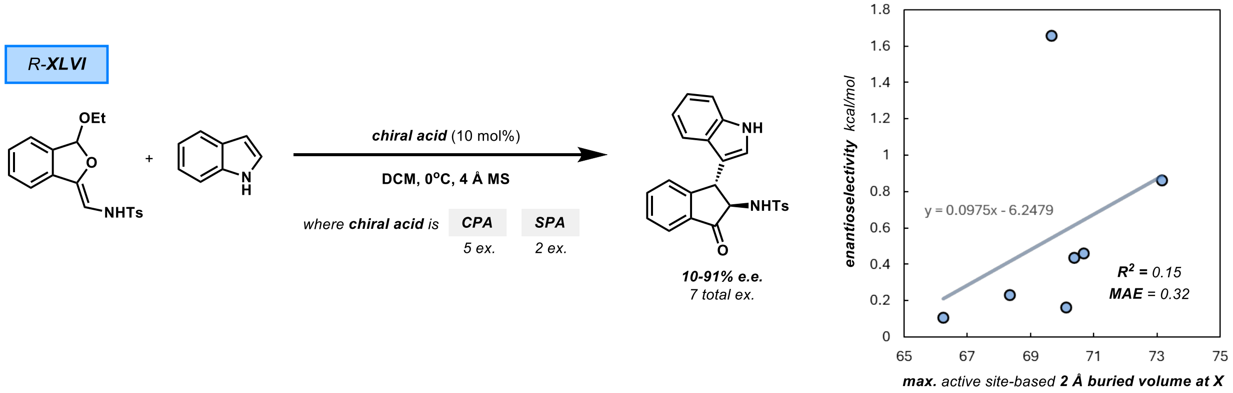

Supplementary Fig. 66. R-**XLVI** correlation drawn by *active site-based* buried volume.

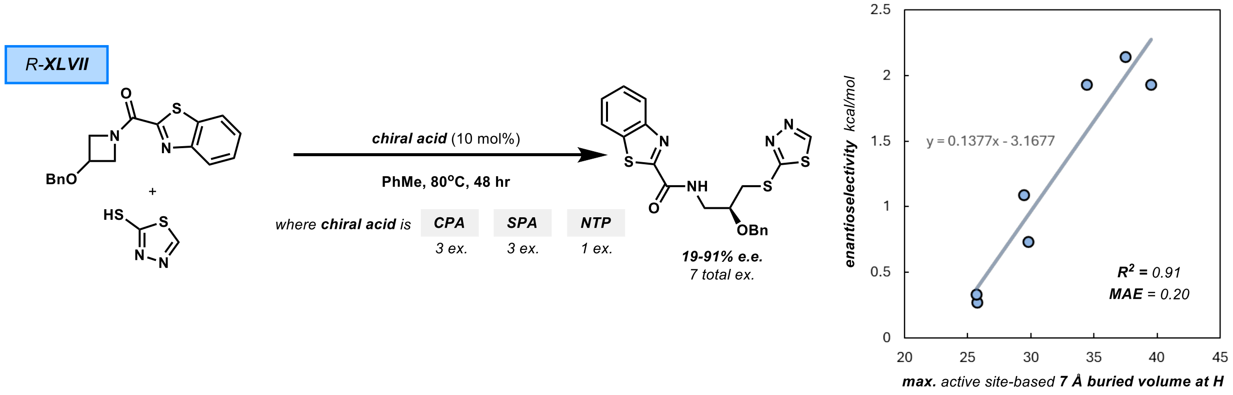

Supplementary Fig. 67. R-**XLVII** correlation drawn by *active site-based* buried volume.

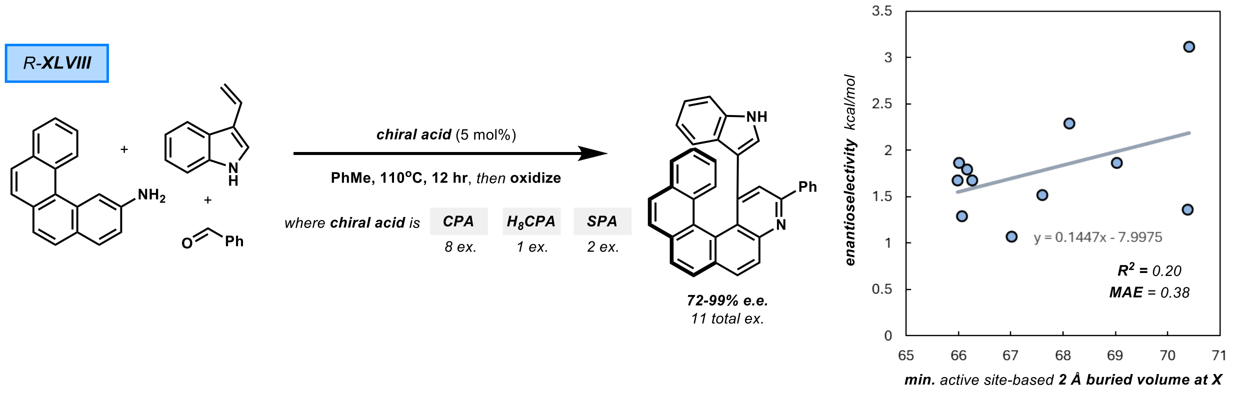

Supplementary Fig. 68. R-**XLVIII** correlation drawn by *active site-based* buried volume.

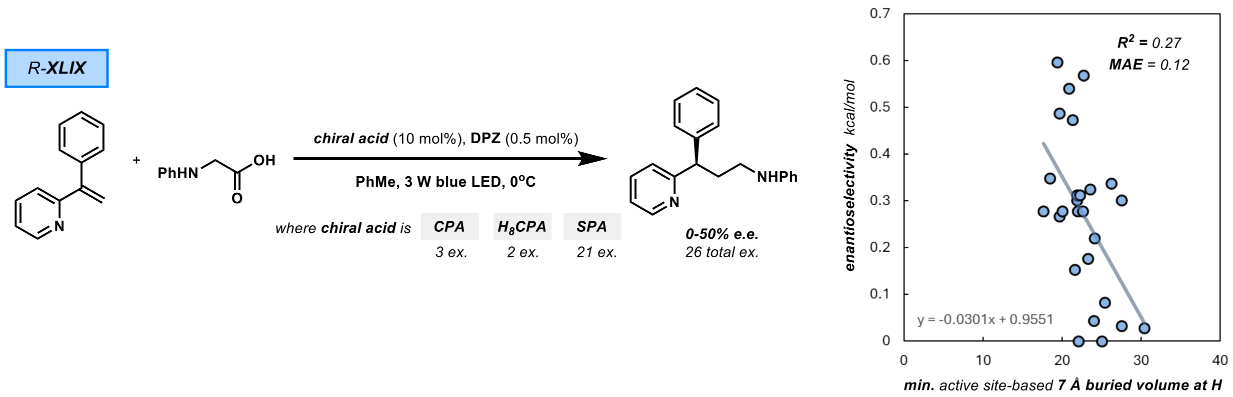

Supplementary Fig. 69. R-**XLIX** correlation drawn by *active site-based* buried volume.

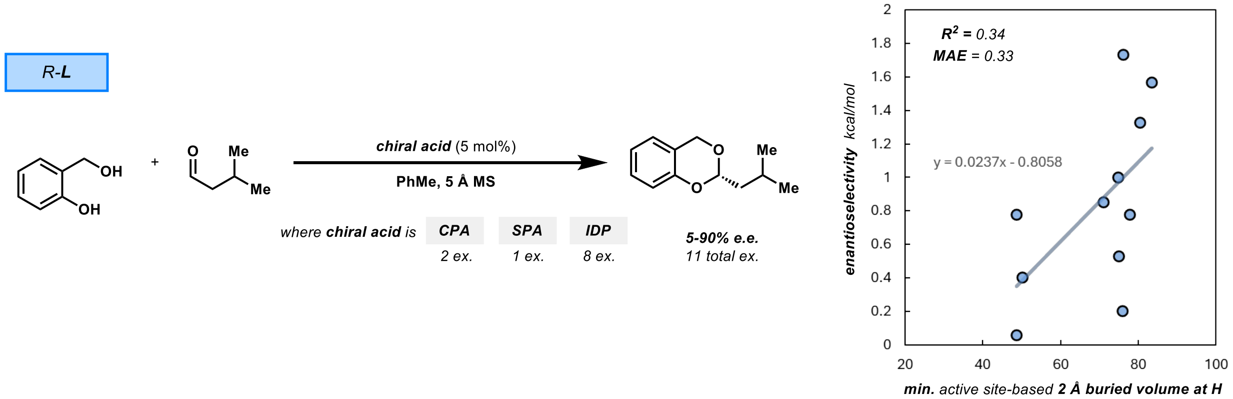

Supplementary Fig. 70. R-**L** correlation drawn by *active site-based* buried volume.

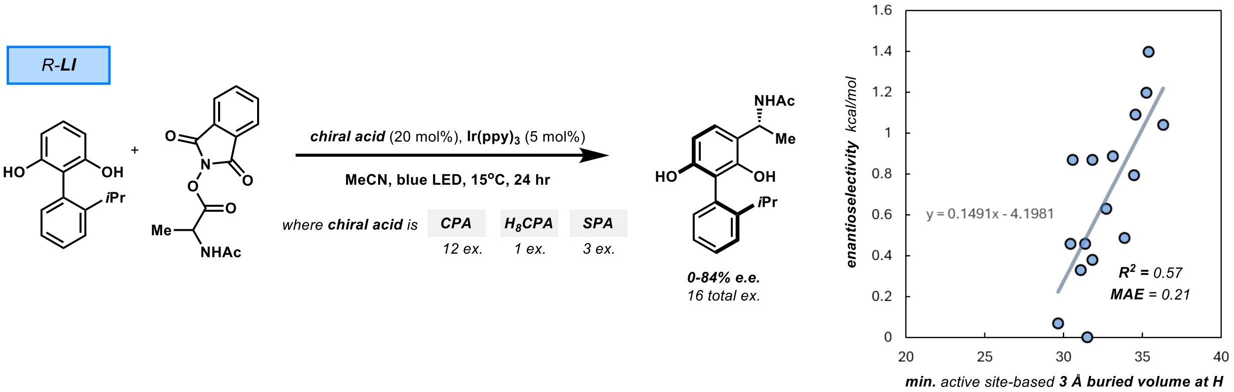

Supplementary Fig. 71. R-**LI** correlation drawn by *active site-based* buried volume.

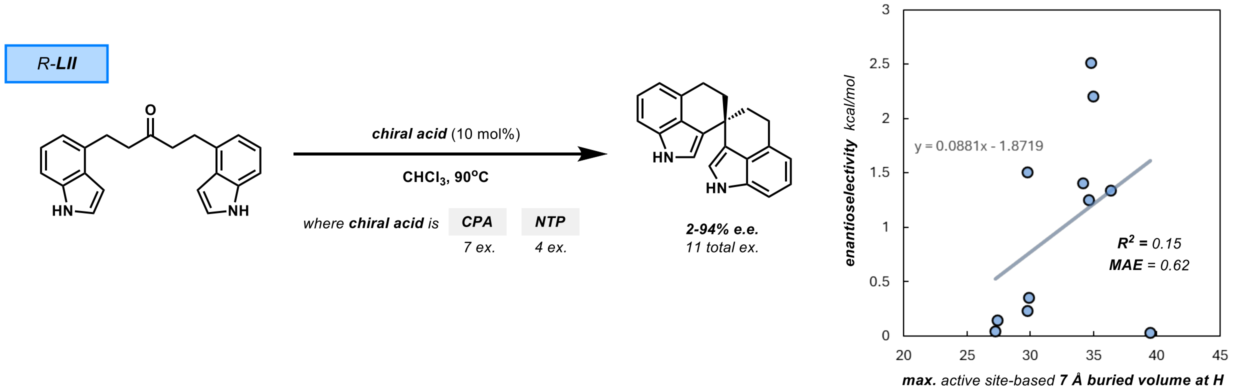

Supplementary Fig. 72. R-**LII** correlation drawn by *active site-based* buried volume.

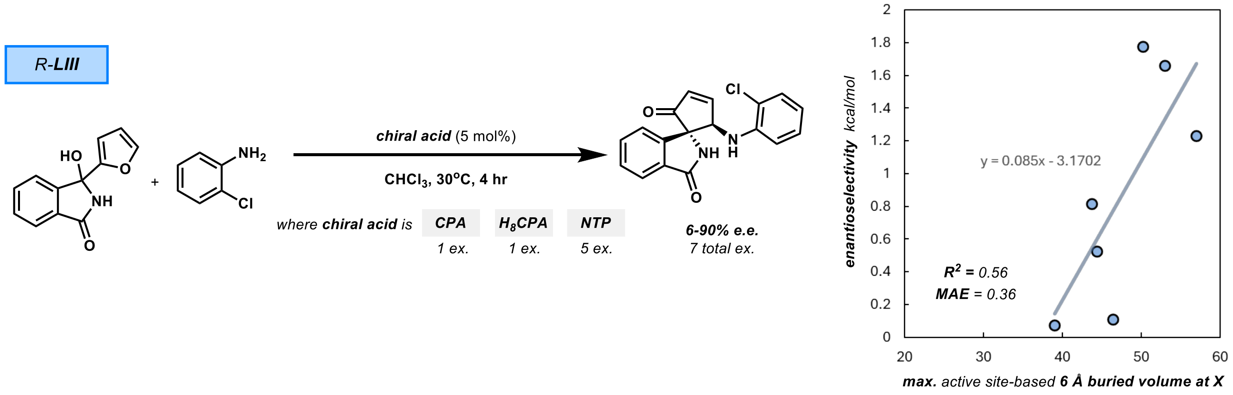

Supplementary Fig. 73. R-**LIII** correlation drawn by *active site-based* buried volume.

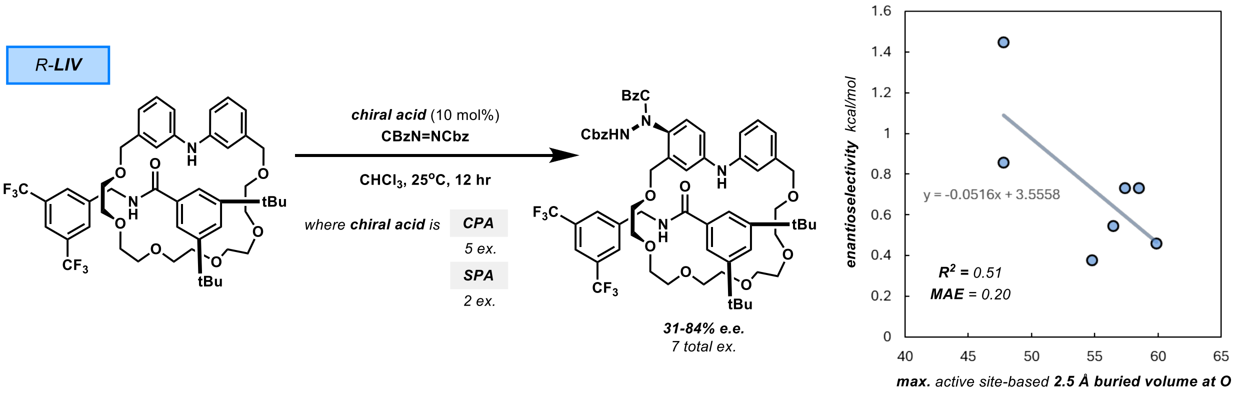

Supplementary Fig. 74. R-**LIV** correlation drawn by *active site-based* buried volume.

References

(1) Li, X.; Duan, M.; Yu, P.; Houk, K. N.; Sun, J. Organocatalytic Enantioselective Dearomatization of Thiophenes by 1,10-Conjugate Addition of Indole Imine Methides. *Nat Commun* **2021**, *12* (1), 4881.

(2) Wang, Y.; Wang, S.; Shan, W.; Shao, Z. Direct Asymmetric N-Propargylation of Indoles and Carbazoles Catalyzed by Lithium SPINOL Phosphate. *Nat Commun* **2020**, *11* (1), 226.

(3) Zhang, Y.-Q.; Chen, Y.-B.; Liu, J.-R.; Wu, S.-Q.; Fan, X.-Y.; Zhang, Z.-X.; Hong, X.; Ye, L.-W. Asymmetric Dearomatization Catalysed by Chiral Brønsted Acids via Activation of Ynamides. *Nat. Chem.* **2021**, *13* (11), 1093–1100.

(4) Xia, Z.-L.; Zheng, C.; Xu, R.-Q.; You, S.-L. Chiral Phosphoric Acid Catalyzed Aminative Dearomatization of α-Naphthols/Michael Addition Sequence. *Nat Commun* **2019**, *10* (1), 3150.

(5) Zheng, W.-F.; Chen, J.; Qi, X.; Huang, Z. Modular and Diverse Synthesis of Amino Acids via Asymmetric Decarboxylative Protonation of Aminomalonic Acids. *Nat. Chem.* **2023**, *15* (12), 1672–1682.

(6) Wei, Y.; Sun, F.; Li, G.; Xu, S.; Zhang, M.; Hong, L. Enantioselective Synthesis of N–N Amide–Pyrrole Atropisomers via Paal–Knorr Reaction. *Org. Lett.* **2024**, *26* (12), 2343–2348.

(7) Wu, F.; Zhang, Y.; Zhu, R.; Huang, Y. Discovery and Synthesis of Atropisomerically Chiral Acyl-Substituted Stable Vinyl Sulfoxonium Ylides. *Nat. Chem.* **2024**, *16* (1), 132–139.

(8) Wen, L.; Ding, J.; Duan, L.; Wang, S.; An, Q.; Wang, H.; Zuo, Z. Multiplicative Enhancement of Stereoenrichment by a Single Catalyst for Deracemization of Alcohols. *Science* **2023**, *382* (6669), 458–464.

(9) Liao, S.; Čorić, I.; Wang, Q.; List, B. Activation of H2O2 by Chiral Confined Brønsted Acids: A Highly Enantioselective Catalytic Sulfoxidation. *J. Am. Chem. Soc.* **2012**, *134* (26), 10765–10768.

(10) Kwon, Y.; Li, J.; Reid, J. P.; Crawford, J. M.; Jacob, R.; Sigman, M. S.; Toste, F. D.; Miller, S. J. Disparate Catalytic Scaffolds for Atroposelective Cyclodehydration. *J. Am. Chem. Soc.* **2019**, *141* (16), 6698–6705.

(11) Saito, K.; Moriya, Y.; Akiyama, T. Chiral Phosphoric Acid Catalyzed Asymmetric Synthesis of 2-Substituted 2,3-Dihydro-4-Quinolones by a Protecting-Group-Free Approach. *Org. Lett.* **2015**, *17* (13), 3202–3205.

(12) Yang, W.; Sun, J. Organocatalytic Enantioselective Synthesis of 1,4-Dioxanes and Other Oxa-Heterocycles by Oxetane Desymmetrization. *Angewandte Chemie International Edition* **2016**, *55* (5), 1868–1871.

(13) Gu, Z.; Zhang, L.; Li, H.; Cao, S.; Yin, Y.; Zhao, X.; Ban, X.; Jiang, Z. Deracemization through Sequential Photoredox-Neutral and Chiral Brønsted Acid Catalysis. *Angewandte Chemie International Edition* **2022**, *61* (49), e202211241.

(14) Song, X.; Zhang, Y.; Li, Y.; Zhao, X.; Yin, Y.; Ban, X.; Jiang, Z. Catalytic Asymmetric Synthesis of Azaarene-Functionalized Tertiary Amines and α-Amino Acid Derivatives from E/Z-Ketimine Mixtures via Enantioselective Radical Coupling. *ACS Catal.* **2023**, *13* (9), 6396–6402.

(15) Ding, W.-W.; He, Z.-Y.; Sayed, M.; Zhou, Y.; Han, Z.-Y.; Gong, L.-Z. Enantioselective Synthesis of β- and α-Amino Ketones through Reversible Alkane Carbonylation. *Nat. Synth* **2024**, *3* (4), 507–516.

(16) Subba, P.; Sahoo, S. R.; Khajuria, C.; Singh, V. K. Enantioselective Aminative Dearomatization of Indoles via Electrophilic 1,6-Addition of p-Quinone Diimides (p-QDIs). *Org. Lett.* **2024**, *26* (23), 4932–4937.

(17) Yu, H.; Hu, L.; Zhang, J.; Yang, Q.; Lu, G.; Xu, T. Organocatalytic Highly Enantioselective Formal [1,3] Sigmatropic Rearrangement of Indole Alkyl Ethers and Mechanistic Insight. *ACS Catal.* **2024**, 9207–9216.

(18) Wei, W.; Li, C.; Fan, Y.; Chen, X.; Zhao, X.; Qiao, B.; Jiang, Z. Catalytic Asymmetric Redox-Neutral [3+2] Photocycloadditions of Cyclopropyl Ketones with Vinylazaarenes Enabled by Consecutive Photoinduced Electron Transfer. *Angewandte Chemie International Edition* **2024**, *63* (30), e202406845.

(19) Liang, D.; Chen, J.-R.; Tan, L.-P.; He, Z.-W.; Xiao, W.-J. Catalytic Asymmetric Construction of Axially and Centrally Chiral Heterobiaryls by Minisci Reaction. *J. Am. Chem. Soc.* **2022**, *144* (13), 6040–6049.

(20) Yang, J.; Zhang, J.-W.; Bao, W.; Qiu, S.-Q.; Li, S.; Xiang, S.-H.; Song, J.; Zhang, J.; Tan, B. Chiral Phosphoric Acid-Catalyzed Remote Control of Axial Chirality at Boron–Carbon Bond. *J. Am. Chem. Soc.* **2021**, *143* (33), 12924–12929.

(21) Ye, Z.; Xie, W.; Wang, D.; Liu, H.; Yang, X. Atroposelective Synthesis of Diarylamines via Organocatalyzed Electrophilic Amination. *ACS Catal.* **2024**, *14* (7), 4958–4967.

(22) Yue, T.; Wang, M.-X.; Wang, D.-X.; Masson, G.; Zhu, J. Brønsted Acid Catalyzed Enantioselective Three-Component Reaction Involving the α Addition of Isocyanides to Imines. *Angewandte Chemie International Edition* **2009**, *48* (36), 6717–6721.

(23) Zahrt, A. F.; Henle, J. J.; Rose, B. T.; Wang, Y.; Darrow, W. T.; Denmark, S. E. Prediction of Higher-Selectivity Catalysts by Computer-Driven Workflow and Machine Learning. *Science* **2019**, *363* (6424), eaau5631.

(24) Rueping, M.; Antonchick, A. P.; Sugiono, E.; Grenader, K. Asymmetric Brønsted Acid Catalysis: Catalytic Enantioselective Synthesis of Highly Biologically Active Dihydroquinazolinones. *Angewandte Chemie International Edition* **2009**, *48* (5), 908–910.

(25) Shevchenko, G. A.; Oppelaar, B.; List, B. An Unexpected α-Oxidation of Cyclic Ketones with 1,4-Benzoquinone by Enol Catalysis. *Angewandte Chemie International Edition* **2018**, *57* (33), 10756–10759.

(26) Kötzner, L.; Webber, M. J.; Martínez, A.; De Fusco, C.; List, B. Asymmetric Catalysis on the Nanoscale: The Organocatalytic Approach to Helicenes. *Angewandte Chemie International Edition* **2014**, *53* (20), 5202–5205.

(27) Rueping, M.; Antonchick, A. P. Catalytic Asymmetric Aminoallylation of Aldehydes: A Catalytic Enantioselective Aza-Cope Rearrangement. *Angewandte Chemie International Edition* **2008**, *47* (52), 10090–10093.

(28) Zhao, Q.; Li, Y.; Zhang, Q.-X.; Cheng, J.-P.; Li, X. Catalytic Asymmetric Aza-Diels–Alder Reaction of Ketimines and Unactivated Dienes. *Angewandte Chemie International Edition* **2021**, *60* (32), 17608–17614.

(29) Biswas, S.; Kubota, K.; Orlandi, M.; Turberg, M.; Miles, D. H.; Sigman, M. S.; Toste, F. D. Enantioselective Synthesis of N,S-Acetals by an Oxidative Pummerer-Type Transformation Using Phase-Transfer Catalysis. *Angewandte Chemie International Edition* **2018**, *57* (2), 589–593.

(30) Terada, M.; Soga, K.; Momiyama, N. Enantioselective Activation of Aldehydes by Chiral Phosphoric Acid Catalysts in an Aza-Ene-Type Reaction between Glyoxylate and Enecarbamate. *Angewandte Chemie International Edition* **2008**, *47* (22), 4122–4125.

(31) Li, G.; Yao, Y.; Wang, Z.; Zhao, M.; Xu, J.; Huang, L.; Zhu, G.; Bao, G.; Sun, W.; Hong, L.; Wang, R. Switchable Skeletal Rearrangement of Dihydroisobenzofuran Acetals with Indoles. *Org. Lett.* **2019**, *21* (11), 4313–4317.

(32) Wang, Z.; Sheong, F. K.; Sung, H. H. Y.; Williams, I. D.; Lin, Z.; Sun, J. Catalytic Enantioselective Intermolecular Desymmetrization of Azetidines. *J. Am. Chem. Soc.* **2015**, *137* (18), 5895–5898.

(33) Mo, N.-F.; Zhang, Y.; Guan, Z.-H. Highly Enantioselective Three-Component Povarov Reaction for Direct Construction of Azaspirocycles. *Org. Lett.* **2022**, *24* (35), 6397–6401.

(34) He, L.; Bekkaye, M.; Retailleau, P.; Masson, G. Chiral Phosphoric Acid Catalyzed Inverse-Electron-Demand Aza-Diels–Alder Reaction of Isoeugenol Derivatives. *Org. Lett.* **2012**, *14* (12), 3158–3161.

(35) Li, C.; Shao, Y.-B.; Gao, X.; Ren, Z.; Guo, C.; Li, M.; Li, X. Enantioselective Synthesis of Chiral Quinohelicenes through Sequential Organocatalyzed Povarov Reaction and Oxidative Aromatization. *Nat Commun* **2023**, *14* (1), 3380.

(36) Hoffmann, S.; Seayad, A. M.; List, B. A Powerful Brønsted Acid Catalyst for the Organocatalytic Asymmetric Transfer Hydrogenation of Imines. *Angewandte Chemie International Edition* **2005**, *44* (45), 7424–7427.

(37) Rueping, M.; Sugiono, E.; Azap, C.; Theissmann, T.; Bolte, M. Enantioselective Brønsted Acid Catalyzed Transfer Hydrogenation: Organocatalytic Reduction of Imines. *Org. Lett.* **2005**, *7* (17), 3781–3783.

(38) Li, Y.-P.; Li, Z.-Q.; Zhou, B.; Li, M.-L.; Xue, X.-S.; Zhu, S.-F.; Zhou, Q.-L. Chiral Spiro Phosphoric Acid-Catalyzed Friedel–Crafts Conjugate Addition/Enantioselective Protonation Reactions. *ACS Catal.* **2019**, *9* (7), 6522–6529.

(39) Meng, S.-S.; Tang, W.-B.; Zheng, W.-H. Catalytically Enantioselective Synthesis of Acyclic α-Tertiary Amines through Desymmetrization of 2-Substituted 2-Nitro-1,3-Diols. *Org. Lett.* **2018**, *20* (3), 518–521.

(40) Yin, Y.; Dai, Y.; Jia, H.; Li, J.; Bu, L.; Qiao, B.; Zhao, X.; Jiang, Z. Conjugate Addition–Enantioselective Protonation of N-Aryl Glycines to α-Branched 2-Vinylazaarenes via

Cooperative Photoredox and Asymmetric Catalysis. *J. Am. Chem. Soc.* **2018**, *140* (19), 6083–6087.

(41) Cheng, X.; Vellalath, S.; Goddard, R.; List, B. Direct Catalytic Asymmetric Synthesis of Cyclic Aminals from Aldehydes. *J. Am. Chem. Soc.* **2008**, *130* (47), 15786–15787.

(42) Kim, J. H.; Čorić, I.; Vellalath, S.; List, B. The Catalytic Asymmetric Acetalization. *Angewandte Chemie International Edition* **2013**, *52* (16), 4474–4477.

(43) Mao, K.; Liu, C.; Wang, Y.; Gu, C.; Putziger, J. M.; Cemalovic, N. I.; Muniz, C.; Qi, Y.; Lin, S. Dynamic Kinetic Resolution of Phosphines with Chiral Supporting Electrolytes. *Nature* **2025**, *643* (8074), 1288–1296.

(44) Sanocki, M.; Russell, H. C.; Handjaya, J.; Reid, J. P. Relative Generality and Risk: Quantitative Measures for Broad Catalyst Success. *ACS Catal.* **2024**, *14* (22), 16849–16860.

(45) Moon, J.; Shin, E.; Kwon, Y. Enantioselective Desymmetrization of Biaryls via Cooperative Photoredox/Brønsted Acid Catalysis and Its Application to the Total Synthesis of Ancistrobrevolines. *J. Am. Chem. Soc.* **2025**, *147* (15), 12800–12810.

(46) Zhao, H.-W.; Jiang, F.; Chen, S.; Hu, J.; Xiang, S.-H.; Ding, W.-Y.; Lu, W.; Tan, B. Organocatalytic Asymmetric Construction and Application of Axially Chiral Spiro-Bisindoles. *Angewandte Chemie International Edition* **2025**, *64* (12), e202422951.

(47) Guo, M. C.; Miller, S. J. Catalyst–Substrate Pairings for Carbocyclic and Heterocyclic Systems in Atroposelective Quinazolinone Synthesis. *ACS Catal.* **2024**, *14* (23), 17226–17232.

(48) Yu, L.; Tang, Y.; Nie, X.; Cai, Y. Stereoselective Access to Spiro-Isoindolinone Scaffolds via Catalytic Asymmetric Aza-Piancatelli Rearrangement. *Org. Lett.* **2024**, *26* (36), 7667–7671.

(49) Wang, Y.; Song, R.-P.; Li, X.-Y.; Chen, W.-L.; Tian, Y.; Zhang, S.-H.; Shao, Y.-D.; Cheng, D.-J. Catalytic Asymmetric Reductive Amination for Axially Chiral Aryl Aldehydes via Desymmetrization/Kinetic Resolution Cascade. *Org. Lett.* **2024**, *26* (34), 7161–7165.

(50) Huang, D.; Li, X.; Xu, F.; Li, L.; Lin, X. Highly Enantioselective Synthesis of Dihydroquinazolinones Catalyzed by SPINOL-Phosphoric Acids. *ACS Catal.* **2013**, *3* (10), 2244–2247.

(51) Tang, M.; Zhou, J.; Xie, W.; Ren, J.; Ye, Z.; Gu, H.; Yang, X. Catalytic Enantioselective Synthesis of Mechanically Planar Chiral Rotaxanes by Organocatalyzed Desymmetrization. *Chem* **2025**, 102694.

(52) Bannwarth, C.; Ehlert, S.; Grimme, S. GFN2-xTB—An Accurate and Broadly Parametrized Self-Consistent Tight-Binding Quantum Chemical Method with Multipole Electrostatics and Density-Dependent Dispersion Contributions. *J. Chem. Theory Comput.* **2019**, *15* (3), 1652–1671.

(53) Pracht, P.; Grimme, S.; Bannwarth, C.; Bohle, F.; Ehlert, S.; Feldmann, G.; Gorges, J.; Müller, M.; Neudecker, T.; Plett, C.; Spicher, S.; Steinbach, P.; Wesołowski, P. A.; Zeller, F. CREST—A Program for the Exploration of Low-Energy Molecular Chemical Space. *The Journal of Chemical Physics* **2024**, *160* (11), 114110.

(54) Gaussian 16, Revision C.01, Frisch, M. J.; Trucks, G. W.; Schlegel, H. B.; Scuseria, G. E.; Robb, M. A.; Cheeseman, J. R.; Scalmani, G.; Barone, V.; Petersson, G. A.; Nakatsuji, H.; Li, X.; Caricato, M.; Marenich, A. V.; Bloino, J.; Janesko, B. G.; Gomperts, R.; Mennucci, B.; Hratchian, H. P.; Ortiz, J. V.; Izmaylov, A. F.; Sonnenberg, J. L.; Williams-Young, D.; Ding, F.; Lipparini, F.; Egidi, F.; Goings, J.; Peng, B.; Petrone, A.; Henderson, T.; Ranasinghe, D.; Zakrzewski, V. G.; Gao, J.; Rega, N.; Zheng, G.; Liang, W.; Hada, M.; Ehara, M.; Toyota, K.; Fukuda, R.; Hasegawa, J.; Ishida, M.; Nakajima, T.; Honda, Y.; Kitao, O.; Nakai, H.; Vreven, T.; Throssell, K.; Montgomery, J. A., Jr.; Peralta, J. E.; Ogliaro, F.; Bearpark, M. J.; Heyd, J. J.; Brothers, E. N.; Kudin, K. N.; Staroverov, V. N.; Keith, T. A.; Kobayashi, R.; Normand, J.; Raghavachari, K.; Rendell, A. P.; Burant, J. C.; Iyengar, S. S.; Tomasi, J.; Cossi, M.; Millam, J. M.; Klene, M.; Adamo, C.; Cammi, R.; Ochterski, J. W.; Martin, R. L.; Morokuma, K.; Farkas, O.; Foresman, J. B.; Fox, D. J. Gaussian, Inc., Wallingford CT, 2016.

(55) Greg Landrum; Paolo Tosco; Brian Kelley; Ricardo Rodriguez; David Cosgrove; Riccardo Vianello; sriniker; gedeck; Gareth Jones; NadineSchneider; Eisuke Kawashima; Dan Nealschneider; Andrew Dalke; Matt Swain; Brian Cole; Samo Turk; Aleksandr Savelev; Alain Vaucher; Maciej Wójcikowski; Ichiru Take; Vincent F. Scalfani; Rachel Walker; Kazuya Ujihara; Daniel Probst; guillaume godin; Axel Pahl; Juuso Lehtivarjo; Francois Berenger; jasondbiggs; strets123. Rdkit/Rdkit: 2024_03_1 (Q1 2024) Release, 2024.

(56) Falivene, L.; Cao, Z.; Petta, A.; Serra, L.; Poater, A.; Oliva, R.; Scarano, V.; Cavallo, L. Towards the Online Computer-Aided Design of Catalytic Pockets. *Nat. Chem.* **2019**, *11* (10), 872–879.

(57) Treacy, S. M.; Smith, A. L.; Bergman, R. G.; Raymond, K. N.; Toste, F. D. Supramolecular Catalyzed Cascade Reduction of Azaarenes Interrogated via Data Science. *J. Am. Chem. Soc.* **2024**, *146* (43), 29792–29800.

(58) Guilian Luchini; Patterson, T.; Paton, R. Patonlab/DBSTEP: Release 1.1.0, 2023.

(59) Kariofillis, S. K.; Jiang, S.; Żurański, A. M.; Gandhi, S. S.; Martinez Alvarado, J. I.; Doyle, A. G. Using Data Science To Guide Aryl Bromide Substrate Scope Analysis in a Ni/Photoredox-Catalyzed Cross-Coupling with Acetals as Alcohol-Derived Radical Sources. *J. Am. Chem. Soc.* **2022**, *144* (2), 1045–1055.

(60) Kütt, A.; Tshepelevitsh, S.; Saame, J.; Lõkov, M.; Kaljurand, I.; Selberg, S.; Leito, I. Strengths of Acids in Acetonitrile. *European Journal of Organic Chemistry* **2021**, *2021* (9), 1407–1419.
